# A data-driven analysis of patient selection for xenotransplant human clinical trials

**DOI:** 10.1371/journal.pone.0335767

**Published:** 2025-12-01

**Authors:** Baris Ata, Robert A. Montgomery, Yucel Naz Ozyoruk, Brendan Parent, Jesse D. Schold

**Affiliations:** 1 The University of Chicago Booth School of Business, Chicago, Illinois, United States of America; 2 NYU Langone Transplant Institute, NYU Langone Health, New York, New York, United States of America; 3 The Department of Population Health, Division of Medical Ethics, NYU Langone Health, New York, New York, United States of America; 4 Department of Surgery, University of Colorado Anschutz Medical Campus, Aurora, Colorado, United States of America; Fudan University, CHINA

## Abstract

The demand for transplant organs far exceeds the available supply. In the United States alone, more than 90,000 patients are currently on the kidney transplant waitlist, yet only about one third of them will ever receive a transplant. Xenotransplantation, organ transplants from gene edited pigs, offers a potential solution to this shortage. Successful investigational transplants of pig kidneys into brain-dead recipients and expanded access cases involving living human recipients have resulted in the green-lighting of the first human clinical trials. Using the benchmark of 2-year survival of non-human primates in pre-clinical studies, we developed a tool that can identify individual wait-listed patients predicted to have a shorter life expectancy than with a xenotransplant, utilizing Random Survival Forest, DeepSurv and Cox Proportional-Hazards models. We found that it is hard to identify patients that reach clinical equipoise unless the expected xenograft survival exceeds two years, with the Random Survival Forest model identifying less than 5% of such patients. Few patients would benefit based on survival alone and potential beneficiaries are spread across more than 200 transplant centers. Several incentives could allow more patients to reach equipoise. At the same benchmark of 2-year xenograft survival, keeping patients inactive on the waitlist while they have a functioning xeno-kidney increases the percentage achieving equipoise by up to 1.7% across cohorts. Granting patients with failed xenografts the same priority as prior living donors increases this by up to 17.9%, while assigning them the highest priority raises it by up to 28.5%. We are able, however, to identify phenotypes that have a high mortality and low transplant rates in the current allocation system that could serve as acceptable candidates; while not achieving equipoise, they would enjoy the benefits of being dialysis free.

## 1 Introduction

There are over 90,000 patients on the deceased-donor kidney transplant waitlist as of February 2025, see [1]. Only one third of them will ever receive an organ. The other two thirds will become too sick to benefit from transplantation or will die waiting. Only 4% of the patients with end-stage renal disease (ESRD) receive a kidney each year. Despite ongoing efforts for decades, organ shortage remains the greatest unmet need in transplantation. However, the recent advances in xenotransplantation presents a new opportunity for alleviating the organ shortage. Pigs are the most promising source of organs for humans in terms of size, availability, public perception, breeding characteristics, ease of cloning/gene editing and physiologic similarities to humans [2]. Genetic engineering introduces essential modifications to address inter-species incompatibilities and are essential for successful engraftment [3]. Xenotransplantation has several advantages over allotransplantation in terms of providing a sustainable supply of life-saving organs and potentially ending the organ shortage. Elective transplants can also be offered to patients pre-emptively, eliminating the need for dialysis [4].

Recent progress demonstrates that xenotransplantation is moving rapidly from concept to clinical reality. The first investigational transplant of a genetically engineered pig kidney into a brain-dead human was performed at NYU Langone Health in September 2021, with graft function maintained for 54 hours without hyperacute rejection [5,6]. A subsequent case at the University of Alabama at Birmingham confirmed feasibility [7], and in 2023 a porcine kidney at NYU Langone sustained function for two months [8]. In addition, Massachusetts General Hospital reported immediate function after transplanting a genetically modified pig kidney into a living patient in March 2024 [9], followed by a combined left ventricular assist device and alpha-Gal knockout thymokidney transplant at NYU Langone in April 2024 [10]. At the University of Maryland, two patients with end-stage heart failure received 10-gene–edited pig hearts, both surviving less than two months but providing critical insights into immune mechanisms of rejection [11,12]. Together, these developments illustrate both the feasibility and the remaining uncertainties of xenotransplantation.

While these milestones show remarkable scientific progress, they also highlight major uncertainties and challenges for national use. A central concern is the durability of xenografts. In non-human primates, kidney xenografts have survived for less than two years, and it remains uncertain whether these outcomes will translate to humans, since only a few procedures under FDA expanded access have been performed to date. Another unknown is what happens to patients after xenograft failure. Exposure to animal organs may increase sensitization and raise CPRA scores, which could make it harder to find a compatible donor later. Beyond medical uncertainties, there are practical and ethical challenges. Access to trials may depend on where patients are listed, only a limited number of centers may have the required expertise, and rules for re-listing patients after xenograft failure are not yet clear. These uncertainties further highlight the need for human clinical trials.

Xenotransplantation offers transformative opportunities. In the long run, it could enable timely, elective transplantation before dialysis, reducing the morbidity and diminished quality of life associated with long wait times. The potential to develop scalable pig organ production could further alleviate shortages across more than 200 transplant centers nationwide, creating a sustainable supply of life-saving organs. It may also reduce disparities by expanding access to transplantation for patients who currently face long waits or limited donor availability because of sensitization or geographic constraints. In this context, our study provides a data-driven framework to identify patient groups most likely to benefit from early xenotransplant trials and evaluates policy levers that could expand trial participation. These contributions can help guide the responsible design of early clinical trials while paving the way for creating a long-term vision for national implementation.

## 2 Literature review

Several studies highlight the importance of establishing clear and consistent patient selection criteria for xenotransplantation clinical trials.

Jagdale *et al*. [4] provide detailed exclusion criteria for initial human clinical trials, leveraging their extensive clinical expertise. They highlight that in some U.S. states, patients aged 55–65 years with blood group O may face wait times exceeding five years for a donor organ. During this period, more than 50% are likely to die or be removed from the waitlist due to ineligibility for transplantation. Based on this, they recommend that such patients might consider early pig kidney transplantation as an alternative. Our study complements their expert-driven framework by offering a quantitative analysis of patient selection. Similar to Jagdale *et al*. [4], we identify this patient group as having a high level of need. However, our study differs by emphasizing the role of diabetes status in patient selection. Additionally, while their work focuses on clinical criteria, we adopt a data-driven framework, incorporate life expectancy estimates and directly compare the survival benefits of the status quo versus receiving a xenotransplant. Beyond patient selection, we extend the framework by evaluating the role of incentives in achieving clinical equipoise.

Porrett and Locke [13] provide a viewpoint that outlines a high-level roadmap for human trials, highlighting key challenges that should be addressed to ensure recipient safety. They emphasize compatibility testing to detect preformed antibodies, caution against excessive immunosuppression, and highlight the need for pathogen-free donor animal facilities to reduce infection risks. They argue that careful patient selection is necessary, advocating for well-informed and engaged research participants. The paper further highlights that trial design must facilitate data interpretation to ensure meaningful clinical insights. The authors emphasize without careful design, confounding factors may obscure findings and hinder progress in xenotransplantation. This emphasis on interpretable, data-driven decision-making aligns with our study, which provides a rigorous framework for patient selection.

In their review, Carrier *et al*. [14] examine the development of suitable porcine xenografts, immunosuppression protocols for clinical trials, the functional and metabolic capacities of pig organs, recent cases conducted under the FDA’s expanded access provisions, and recipient selection criteria. Summarizing findings from previous studies, they list six broad categories of potential candidates for xenotransplantation: older age, sensitized patients, lack of dialysis access, cultural barriers, non-compliance, and short life expectancy with low quality of life. Our findings align with Carrier *et al*. in identifying older age and shorter life expectancy as key medical covariates for patient selection. While we incorporated socioeconomic variables, they were not among the top indicators of survival.

In their viewpoint, Pierson *et al*. [15] leverage their clinical expertise to explore strategies for selecting the initial recipients of pig-to-human heart transplants. They argue that, for xenotransplantation to be ethically justified, the risks associated with current state-of-the-art treatment options must exceed the anticipated risks of xenotransplantation. They note that renal xenograft clinical trials may progress more quickly than cardiac trials due to the availability of dialysis as a backup option in the case of graft failure. The insights gained from renal xenotransplantation could inform the design and implementation of initial heart xenotransplant trials. Building on their extensive clinical experience, they propose a set of patient characteristics that define potential candidates for cardiac xenotransplantation, such as patients with accelerated cardiac allograft vasculopathy. Our study aligns with their equipoise framework. Complying with their definition of equipoise, we compare life expectancies to identify patient groups for whom the anticipated benefits of xenotransplantation outweigh the risks. Moreover, as mentioned in their study, we consider the possibility of returning to waitlist upon the xenograft failure when estimating the life expectancy under the xenotransplant option. Our study differs from their work in two ways. First, they focus on cardiac xenotransplantation, while we study renal xenotransplantation. This leads to different patient selection criteria and covariates. Second, their approach is based on clinical expertise and observations. In contrast, we use a data-driven method to model patient outcomes and assess xenotransplant feasibility.

Schold *et al*. [16] examine patients’ prognosis on the waiting list and propose that certain patient populations could benefit from accepting donor organs classified as high-risk. They employ cumulative incidence models with competing risks methodology to evaluate mortality and deceased donor transplantation outcomes. Specifically, they estimate the time to equivalent risk (TiTER) for certain patient groups, where TiTER represents the number of months post-listing at which the risk of mortality and the probability of receiving a deceased donor transplant become equal. Using likelihoods derived from cumulative incidence models, they qualitatively conclude that certain demographic groups, such as older patients, those with diabetes, blood type B or O, and shorter pre-listing dialysis durations, face greater challenges on the transplant waitlist and have shorter TiTER. They further use TiTER to quantify the benefits of accepting relatively high-risk donor organs. While our quantitative results differ, our qualitative findings align in identifying these patient groups as high risk. Given these parallel qualitative insights, both studies complement and support each other. However, our study extends their approach in several ways. First, we extend their analysis by incorporating additional patient-specific covariates to determine whether an individual should receive a xenotransplant. While their study provides cohort-level insights, our approach follows a precision medicine framework, allowing for patient-specific recommendations. Second, while they focus on high-risk donor organs, we tailor our analysis exclusively to evaluate the viability of a potential xeno-organ. Third, from a methodological perspective, we take a different approach by directly estimating survival benefits (i.e. life expectancies) under the status quo (without the xenotransplant option) and comparing them with survival benefits with xenotransplantation. In contrast, their study primarily compares the likelihood of receiving a transplant to the likelihood of mortality. Lastly, our work evaluates the impact of potential incentives to achieve equipoise, an aspect not explored in their analysis.

The decision-theoretic literature in organ transplantation traces back to Ahn and Hornberger [17]. Since then, a substantial body of research has emerged at the intersection of operations research and organ transplantation, as summarized by Ata *et al*. [18] and Sekercioglu and Fu [19]. Interestingly, Ahn’s dissertation [20] noted that *"unless xenotransplant becomes possible, or artificial organs are proven to be an effective alternative to real human organs, ESRD patients can expect to wait even longer for a kidney donation."* What was once a theoretical idea is now becoming reality. This marks the beginning of a new era in organ transplantation. Our study contributes to this transition by addressing the patient selection problem. A more extensive review of the decision-theoretic literature initiated by Ahn and Hornberger [17] is beyond the scope of this paper.

Beyond patient selection frameworks, other studies address broader aspects of clinical trial implementation. Adams *et al*. [21] outline regulatory frameworks to ensure safety and compliance in clinical trial designs. Khush *et al*. [22] examine ethical issues, focusing on heart and lung xenotransplantation, including the challenges of informed consent. Bobier *et al*. [23] address fairness in patient selection, stressing the need for equitable access to these innovative trials. Together, these studies provide insights into patient selection, regulatory compliance, ethical considerations, and fairness in xenotransplantation clinical trials.

An antecedent of this paper, Ata *et al*. [24] provides insights using the analysis undertaken in this paper to identify patients who may benefit from xenotransplantation over waiting for an allograft. That study serves as an initial proposal to stimulate discussion within the transplant community while this paper provides the full analysis underlying those insights.

## 3 Methods

This study is a retrospective observational analysis conducted using de-identified data obtained from the Scientific Registry of Transplant Recipients (SRTR) and the United States Renal Data System (USRDS). These datasets include medical information on patients undergoing standard-of-care dialysis and kidney transplantation. No xenotransplantation procedures were performed or included, and no individuals were prospectively assigned to interventions.

This specific study was reviewed and approved by the Institutional Review Board of the Biological Sciences Division at the University of Chicago prior to data access and analysis (IRB21-1442). The IRB determined that the study met the criteria for exemption (secondary research for which consent is not required). As such, informed consent was not required and was not obtained. No direct contact with human subjects occurred, and all data were fully de-identified before access by the investigators. No minors were directly involved.

This study does not meet the criteria for a clinical trial as defined by the World Health Organization (WHO) or the International Committee of Medical Journal Editors (ICMJE), as it does not involve the prospective assignment of participants to interventions. Therefore, clinical trial registration is not applicable.

All analyses were conducted in accordance with the ethical principles. The study adhered to all institutional and federal guidelines governing the use of secondary, de-identified health data.

The data underlying the results presented in this study were obtained from the SRTR and USRDS. These data are not publicly available due to confidentiality and data use agreement restrictions. Researchers who meet the criteria for access may request them via:

- SRTR: https://www.srtr.org/requesting-srtr-data/data-requests/
- USRDS: https://www.niddk.nih.gov/about-niddk/strategic-plans-reports/usrds/

The data were accessed for research purposes between 01/01/2023 and 01/01/2025.

### 3.1 Data sources

This study used data from the Scientific Registry of Transplant Recipients (SRTR). The SRTR data system includes data on all donor, wait-listed candidates, and transplant recipients in the US, submitted by the members of the Organ Procurement and Transplantation Network (OPTN). The Health Resources and Services Administration (HRSA), U.S. Department of Health and Human Services provides oversight to the activities of the OPTN and SRTR contractors. SRTR data from October 1, 1987 to September 1, 2021 were utilized. A description of the CAND KIPA patient file is provided in S1 Appendix G. Deceased-donor organ offer data (match runs, patient rankings, and organ acceptance decisions) covering January 1, 2000 to December 31, 2020 were also used. Additionally, United States Renal Data System (USRDS) core data sets which have information on the outcomes and treatment of chronic kidney disease (CKD) and the ESRD population in the US was analyzed [25]. The Centers for Medicare and Medicaid Services (CMS) Medical Evidence Form (CMS-2728) comorbidity data was accessed to evaluate survival outcomes of ESRD patients. We also obtained the residence information of patients at the ZIP code level. To assess how the socioeconomic status of patients affects their health outcomes, we used the Social Deprivation Index (SDI) which is also available at the ZIP code level. A detailed data description is provided in S1 Appendix F and H.

### 3.2 Study variables

Table 1 shows the complete list of variables that we include from SRTR and USRDS data sets along with the data files. We provide descriptive statistics for the variables in S1 Appendix G.1. Waiting time is incorporated into the analysis because patients’ health conditions can deteriorate on the wait list and they may develop comorbidities, rendering them ineligible for a transplant.

**Table 1:** List of variables.

Category	Data Source	Data File	Variable
Demographics	SRTR	CAND_KIPA	Age at listing
			Education
			Ethnicity
			Race
			Gender
			Source of payment (insurance)
			Employment status
Health status	SRTR	CAND_KIPA	Blood type
			Body mass index (BMI)
			Diabetes (Type 1 and Type 2)
			Primary diagnosis
			Accept an HBC-positive donor?
			Accept an HCV-positive donor?
			Previous malignancy
			Total serum albumin
			Previous transplant indicator
Health status	SRTR	STATHIST_KIPA	cPRA score
Comorbidities	USRDS	MEDEVID	Alcohol dependence
			Drug dependence
			Tobacco use
			Congestive heart failure
			Other cardiac disease
			Atherosclerotic heart disease (ASHD)
			Ischemic heart disease (IHD)
			Myocardial infarction
			Cardiac dysrhythmia
			Pericarditis
			Cardiac arrest
			AIDS
			HIV positive status
			Malignant neoplasm
			Cancer
			Toxic nephropathy
			Non-renal congenital abnormality
Residence	SRTR	INSTITUTION	DSA
Residence	SRTR	CANZIP	ZIP code
Socioeconomic	Robert Graham	ACS2015_ZCTA	SDI score (at the ZIP code level)
status	Center		

Note that we exclude inactive patients from our analysis. Patients with medical conditions that preclude transplantation, such as active malignancies or severe ischemic heart disease, are either not listed on the waitlist or become inactive if such conditions develop after listing. As a result, these patients were not included in our study population. This approach ensures that the analysis reflects only patients who are clinically eligible for transplantation. Further details on how patient inactivity on the waitlist was defined and handled are provided in S1 Appendix H.2.

### 3.3 Analysis

#### 3.3.1 Survival analysis.

To look for patients who stand to benefit from xenotransplantation, we compared the life expectancy of a patient with or without a xeno-kidney. A tool was developed to identify patients at higher risk of death or becoming too sick for an allotransplant and being delisted. Patient characteristics were used to identify those at risk and most likely to benefit from xenotransplantation. The algorithm identified patients whose life expectancy waiting for an allotransplant was less than that associated with a xenotransplant.

Based on a review of contemporary studies in non-human primates, the survival of a life sustaining xeno-kidney was established [2]. It remains uncertain how well the NPH survival metrics will predict results of human xenotransplantation. The expected graft survival of a xeno-kidney in a human was assigned *n* years. The life expectancy with a xenotransplant is then *n* years plus re-listing probability times the life expectancy after re-joining the allotransplant waitlist upon failure of the xeno-kidney. Since *n* is unknown, we perform a sensitivity analysis, considering different values of *n* (n=0.5,1,1.5,2,3,4,5) In addition, the probability of re-joining the waitlist is also unknown. We use the probability of re-listing following the failure of an allograft as a proxy; see S1 Appendix C for details. The set of patients who are identified stand to benefit from xenotransplantation.

To apply this strategy, we estimate how long a patient will survive without a xenotransplant. Using survival curves, the probability of survival at each time point for each patient was calculated and the expected life years was computed. We implemented Random Survival Forest (RSF) and DeepSurv (a model that uses deep neural networks) as well as the traditional Cox Proportional-Hazards (Cox) model to estimate the patients’ life expectancy, [26,27] and [28]; see S1 Appendix I for implementation details.

To date, xenotransplantation has been tested in only a few living patients under FDA expanded access, with outcomes limited and little evidence on post-failure trajectories. In our analysis we assume the CPRA score remains unchanged after xenograft failure, though in reality exposure to animal organs could increase sensitization and reduce future transplant opportunities. Early clinical trials are essential to learn about these effects and to more accurately estimate life expectancy after re-listing.

#### 3.3.2 Performance measures.

The following two key performance measures were of greatest interest:

1. **Hit rate:** Given a certain period of analysis *τ*, it is the fraction of patients who die within *τ* years among those who are identified by the model as having life expectancy of *τ* years or fewer. This metric calculates the fraction of patients correctly labeled by our model.
2. **Capture rate:** This is the fraction of patients identified by the model among those who die within *τ* years. It measures the extent to which we can apriori identify patients who die within *τ* years.

Using the terminology that is standard in machine learning, hit rate and capture rate correspond to precision and recall, respectively; see S1 Appendix K.1 for further details. Although the algorithm tested yielded high hit rates, the capture rate is low when the graft survival is less than three years (see Sect 4). Therefore, we explored how these rates change across different subpopulations of patients. The patients were grouped into cohorts and each is studied separately.

#### 3.3.3 Cohort analysis.

The analysis for certain cohorts of patients was repeated, aiming for an improved capture rate. To determine these cohorts, we considered patient characteristics that affected survival probabilities and the likelihood of receiving a transplant. We obtained variable importance scores to determine the most important variables (see S1 Appendix A.1). We then took the union of the top three variables identified by RSF and Cox models for each analysis; see Table 2. Also see Tables S5 - S8 for further details. The DeepSurv model did not provide a way to identify important variables, so we did not include it in this step. We also tested Calculated Panel-Reactive Antibody (CPRA) and the characteristics of the listing center that contributed to transplant acceptance rates and time to transplant, but neither turned out to be as important as those variables listed in Table 2.

**Table 2:** The top 3 variables identified by RSF and Cox models that affect survival probabilities and the probability of receiving a transplant.

Survival Probability		Probability of Receiving a Transplant	
RSF	Cox	RSF	Cox
Age at listing	Diabetes	Waiting time	Waiting time
Diabetes	Age at listing	Blood type	Previous kidney transplant
Waiting time	Previous kidney transplant	Diabetes	Blood type

Our analysis rests on a tool to identify patients at higher risk of death or becoming too sick for an allotransplant and being delisted. Some of that risk is indeed attributable to center-level waitlist maintenance and organ acceptance practices. To account for this, we developed a measure of transplant center “aggressiveness” based on a patient’s likelihood of receiving a transplant; see S1 Appendix J. Specifically, we calculated two rates: (i) the acceptance rate, defined as the ratio of marginal organs accepted by a center to the total number of such organs offered to its patients, and (ii) the offer seen rate, defined for each patient as the fraction of marginal organs (offered nationally during the study period) that could potentially be seen given the patient’s initial opt-in status, averaged across all patients at the center. We then defined aggressiveness as the product of these two rates, capturing both the willingness of a center to transplant marginal organs and the extent to which patients are exposed to such offers. This measure allows us to incorporate center-level practice variation into the analysis, and we further examined how aggressiveness varies across different patient age groups. Although this variable was not among the most important variables for the RSF and Cox models, it was used in defining the highest-need patient groups.

**Fig 1:**
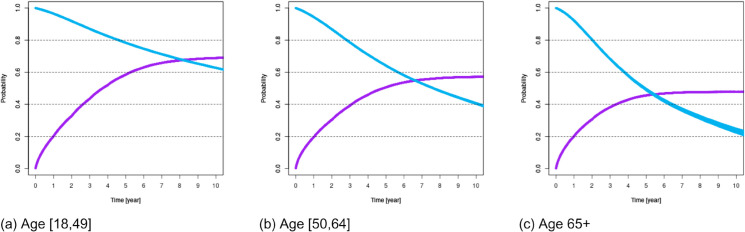
Survival and time-to-transplant curves for patients with different age groups. United Network of Organ Sharing (UNOS) reports the death rate for candidates of age 18-49, 50-64, and 65+ separately. We categorize patients with respect to their ages at registration accordingly.

**Fig 2:**
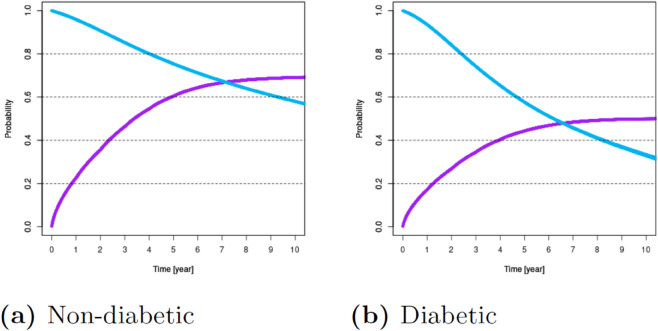
Survival and time-to-transplant curves for non-diabetic and diabetic patients.

**Fig 3:**
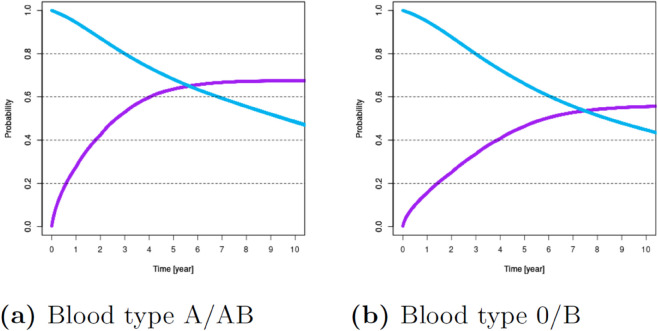
Survival and time-to-transplant curves for patients with blood type A/AB and 0/B.

**Fig 4:**
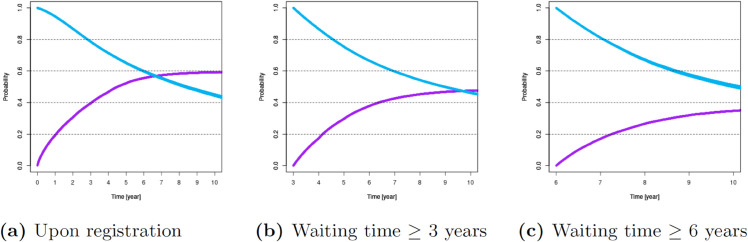
Survival and time-to-transplant curves for patients upon registration, conditional on waiting for 3 and 6 years.

**Fig 5:**
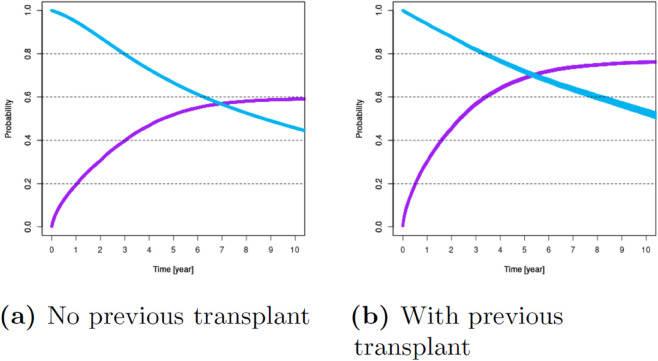
Survival and time-to-transplant curves for patients with and without a previous transplant.

**Fig 6:**
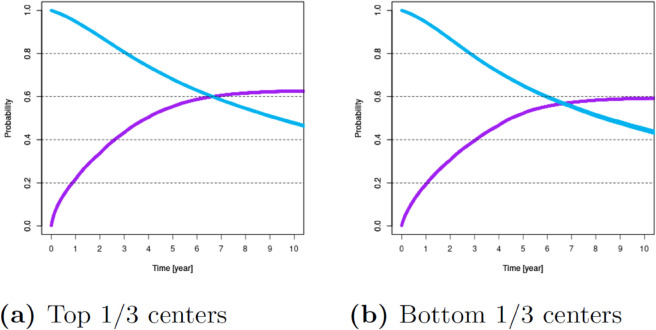
Survival and time-to-transplant curves for patients registered at top 1/3 and bottom 1/3 aggressive centers.

**Fig 7:**
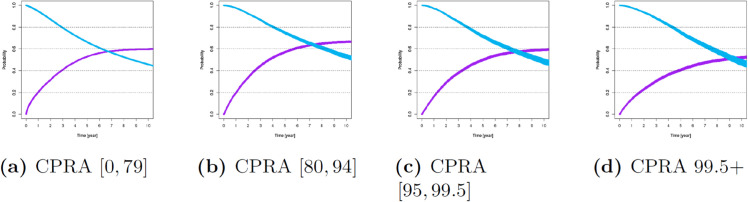
Survival and time-to-transplant curves for patients with different CPRA scores.

For these variables, Figs 1–7 show survival and time-to-transplant curves for different sets of patients where blue and purple curves represent survival probabilities and probability of receiving a transplant, respectively.

Diabetes was the most common variable impacting both survival and transplant probabilities; see Fig 2. Blood types B or O were associated with increased waiting times; see Fig 3. Additionally, age >50 was also associated with diminished survival and transplant rates. Patients ages 18-49 had better chances of survival; see Fig 1.

Patients were further divided into two age groups, 50-65 and 65+ years which are categories used in UNOS analytics. The combination of these three key variables- diabetes, blood type, and age was found to have maximum impact and any further cuts would lower the data size too much for accurate statistical analysis. Next, we brought in variables one at a time. Since waiting time came out as an important variable for both survival and transplant rates, we also considered its effect on the other 3 variables (<3 or ≥3 years of wait time) sequentially for diabetic patients with blood type O/B and aged 50+. Presence of a previous transplant was not common enough in the data set to consider it in the analysis.

Table 3 shows the proposed cohorts. Further details are provided in S1 Appendix A.

**Table 3:** List of cohorts.

Cohort	Explanation
1	Diabetics, Blood type B/O, Age [50,64)
2	Diabetics, Blood type B/O, Age 65+
3	Diabetics, Blood type B/O, Age [50,64), Waiting time ≥ 3 years
4	Diabetics, Blood type B/O, Age 65+, Waiting time ≥ 3 years
5	Diabetics, Blood type B/O, Age [50,64), Waiting time < 3 years
6	Diabetics, Blood type B/O, Age 65+, Waiting time < 3 years
7	Diabetics, Blood type B/O, Age [50,64), Bottom 1/3 aggressive centers
8	Diabetics, Blood type B/O, Age 65+, Bottom 1/3 aggressive centers
9	Diabetics, Blood type B/O, Age [50,64), Top 1/3 aggressive centers
10	Diabetics, Blood type B/O, Age 65+, Top 1/3 aggressive centers
11	CPRA 99.5 or higher

## 4 Results

The algorithm yields high hit rates whereas the capture rate is relatively low when the entire set of patients is considered (Tables S78 and S79 in S1 Appendix K.2). In other words, patients identified by the algorithm are viable subjects for clinical trials compared to waiting for an allograft, but relatively few candidates are identified (e.g., fewer than 5% with the RSF model when *n*<2). Recall that, a patient is considered viable if the predicted life expectancy with a xenotransplant exceeds the expected life expectancy without one. As a robustness check, we also performed an extensive classification analysis using state-of-the-art machine learning techniques (see K.4), but those methods were unreliable with a hit rate of around 50% (no different from flipping a coin).

Results improve as *n*, the expected graft survival of the xeno-kidney, increases. For *n*<2 years, the models are unable to identify many patients whose life expectancy waiting for an allograft in the current system is lower than accepting a xenotransplant. To be more specific, when *n*<2, the RSF model identifies fewer than 5% of such candidates. Hence, the data suggest that less than a 2-year xenograft survival is insufficient to support equipoise versus remaining on the waitlist.

Among the three methods, RSF has the highest hit rate. Cox and Deepsurv models have similar performance. However, the capture rate is lower for RSF. In that sense, it is a more selective approach and appears to make fewer yet more reliable calls for identifying potential subjects for clinical trials.

After concluding that achieving equipoise between xenotransplantation and other options available to ESRD patients is very difficult for xenograft survivals less than 2 years, we sought to identify wait-listed patient groups that would benefit the most from a xeno-kidney. In the cohort analysis, as discussed in Sect 3.3.3, we focused on cohorts for which the probability of survival and the likelihood of receiving an organ were both low. This strategy defined patients with the highest level of need; see Table 3. The number of patients who are identified are reported along with the resulting performance; see Table S81 in S1 Appendix K.2.

**Fig 8:**
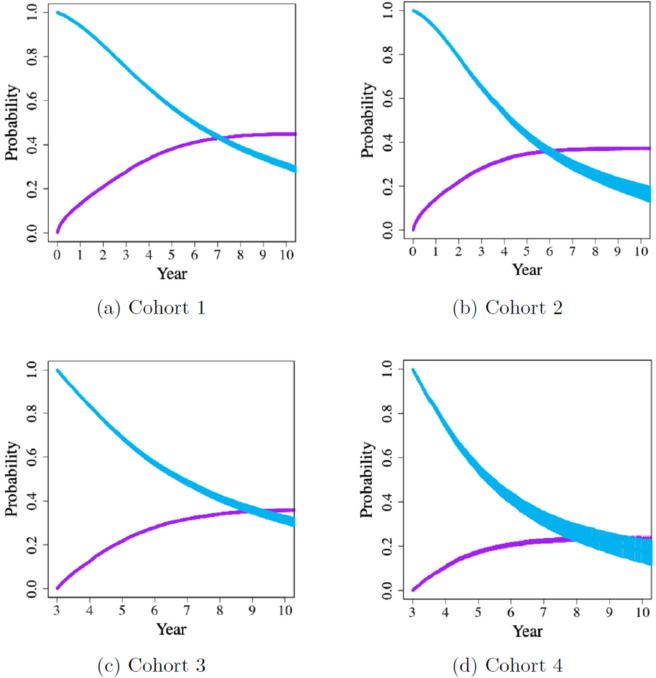
Survival and time-to-transplant curves for four main cohorts. Blue and purple curves represent survival and transplant probabilities, respectively, and the bands around them correspond to 95% confidence intervals. Cohort 1: B/O, diabetic, 50-65. Cohort 2: B/O, diabetic, ≥65. Cohort 3: B/O, diabetic, 50-65, 3 yrs wait time. Cohort 4: B/O, diabetic, ≥65, 3 yrs wait time (taken from Ata *et al*. [24]).

Here, we focus on four of the cohorts that show the greatest benefit from opting for a xenotransplant. Cohorts 1 and 2 consist of patients with blood type B or O who have diabetes and are either between 50 to 65 or older than 65 years, respectively. The remaining two cohorts are subsets of the first two, narrowing our focus to patients who have been on the waiting list for over three years (Cohorts 3 and 4). We provide survival and time-to-transplant curves for these groups of patients in Fig 8. Fig S1 in S1 Appendix B displays the sizes of each cohort across the eight transplant centers that might participate in xenotransplant clinical trials (Table S9 in S1 Appendix B).

**Fig 9:**
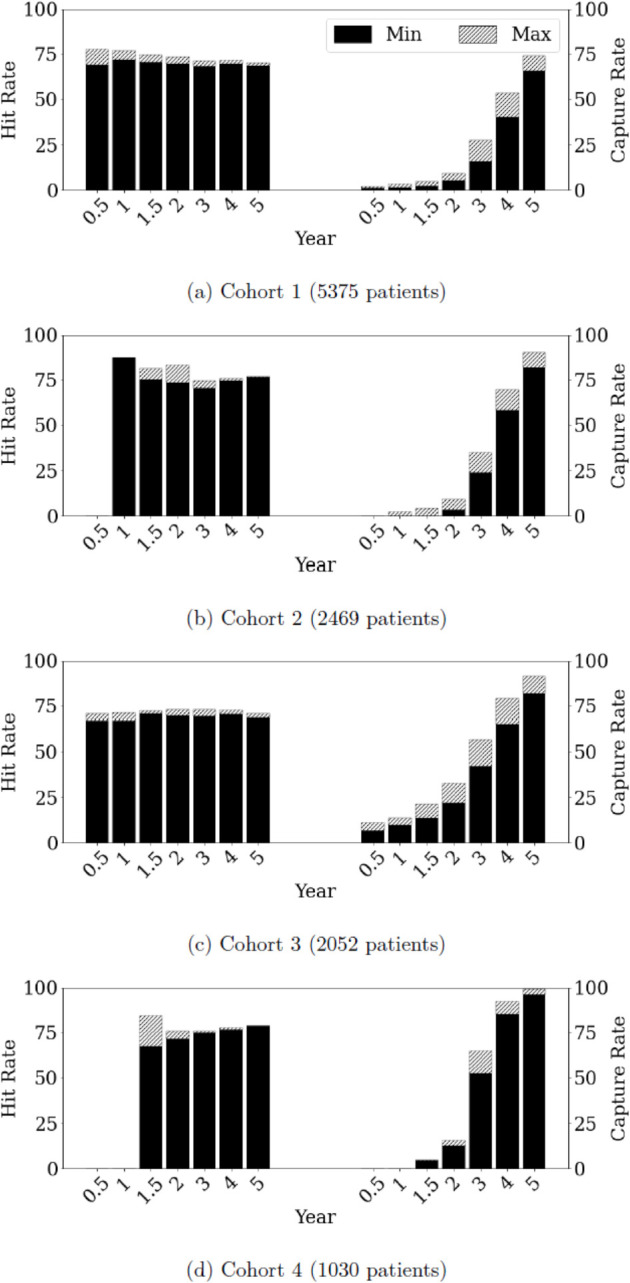
Summary of the sensitivity analysis results for each (cohort, n) pair. In this dual-axis bar chart, the bars positioned on the left depict the minimum and maximum hit rates (among the three models considered) for each year *n*. On the right, the bars display the minimum and maximum capture rates for each year *n*. Results not shown if the number of correctly identified patients is less than 15 for the purpose of statistical accuracy.

Fig 9 presents the sensitivity analysis over different values of *n*, showing the hit and capture rates for each *n* across the four cohorts. The hit rates are consistently high across cohorts (see S1 Appendix A and S1 Appendix K.3 for details). Despite some variation across cohorts, capture rates are low for *n*<2, but they increase as *n* increases. That is to say, it is difficult to identify sufficiently many viable candidates unless the expected graft survival of the xeno-kidney exceeds two years. Hence, our analysis shows again that it is hard to achieve equipoise unless a xeno-kidney provides 2 or more years of survival. It would require offering some incentives toward re-transplantation in the event of xenograft failure to make participation in clinical trials offer a survival benefit.

**Fig 10:**
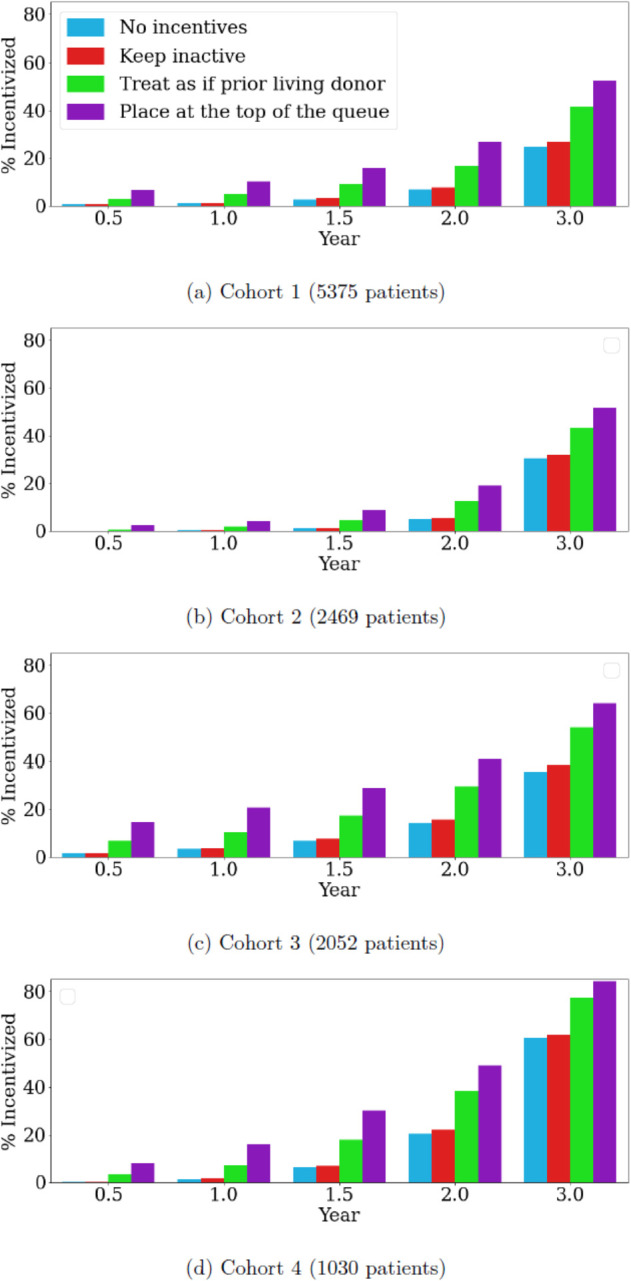
The fraction of incentivized patients under different scenarios for each cohort under the RSF model, with sensitivity analysis conducted over varying values of *n.*

The following incentives were modelled to determine the impact of each on equipoise, with sensitivity analysis conducted over different values of *n*: patient remains inactive on the waitlist during the life of the xenograft, the patient is given the same priority points as a former living donor, or the patient is given the highest priority for their blood group. Blue bars in Fig 10 show the fraction of patients reaching equipoise without any incentives; see S1 Appendix E.2 for details. This serves as the base case to assess the effect of potential incentives. Red bars show the fraction of patients incentivized to accept a xenotransplant by allowing them to remain on the waitlist inactive for each of the four cohorts. Improvement upon the benchmark case is limited inasmuch as there is only a slight increase in the percentage of patients reaching equipoise (2.4 percentage points or less); see S1 Appendix E.3 for detailed results. This observation holds true regardless of the patient cohort and the expected xenograft survival, denoted as *n*.

A counterfactual scenario was examined where a patient receives the same priority points as former living donors who develop ESRD when the xenograft fails (green bars in Fig 10); see S1 Appendix E.4 for detailed results. Lastly, an extreme scenario is considered in which the patient receives the highest priority once the xeno-kidney fails. The assumption is that the patient will receive an ideal organ without waiting. To model this, the pool of donors that the patient can potentially match with is identified. Then the post-transplant life expectancy is calculated when the patient gets matched with each potential donor. Finally, the distribution of the *q*^th^ percentile of post-transplant life expectancy is determined. Fig 10 shows the fraction of the incentivized patients when *q* is set to 75, depicted in purple. In the last scenario, clinical equipoise is reached for a considerable number of patients even when the xenograft survival is shorter than 2 years, resulting in a substantial improvement compared to the base case (see S1 Appendix E.5 for details). Overall, across all four cohorts and *n*, keeping patients inactive increases the fraction reaching equipoise by up to 2.4 percentage points, granting the same priority as prior living donors increases it by up to 18.4 percentage points, and assigning the highest priority raises it by up to 28.5 percentage points.

## 5 Discussion

The scarcity of donor organs continues to be a pressing issue in the field of transplantation. Currently, only a small fraction, 4%, of individuals with organ failure successfully undergo transplantation each year [1]. The majority of patients face either a decline in their health conditions that renders them ineligible for the transplantation or death while awaiting an organ. Despite long-standing efforts to address the organ supply, little progress has been made. Exploring alternative sources of organs is crucial to overcome this persistent challenge. One promising approach is xenotransplantation, which involves transplanting genetically engineered pig organs. Recent breakthroughs in this area [5,29–32] have created the opportunity to move forward with human clinical trials.

To date, only a few living patients have received kidney or heart xenografts under the FDA’s expanded access single-patient Investigational New Drug (IND) pathway. These patients were in end-stage organ failure with no other treatment options and near death. The results from these initial cases have been less favorable compared to the outcomes observed in nonhuman primate studies that formed the basis for the IND approvals [11]. This discrepancy may stem from the recipients’ severe medical conditions. Research involving decedent recipients has highlighted significant gaps in understanding the immunological and physiological responses of transplanted xeno-organs in humans [5,31]. Moving forward, Phase I/II clinical trials involving healthier participants will be necessary to fully evaluate the potential of xenotransplantation in addressing the organ shortage.

A critical question in xenotransplantation research is determining the viable patient population for participation in clinical trials. It has been proposed that participants should not be severely ill that the safety and efficacy of the procedure cannot be accurately evaluated, nor too healthy that the risks involved are disproportionately high. Many stakeholders, including regulatory authorities, recommend selecting patients based on equipoise, ensuring they have comparable alternative options for transplantation. This study explores whether advanced tools such as operations research, mathematical modeling, and state-of-the-art machine learning can identify individual patients or groups who are likely to achieve a survival benefit from xenotransplantation compared to continuing to wait for an allograft.

We present a data-driven approach that accounts for patient-specific covariates to establish selection criteria for xenotransplantation clinical trials. Our methodology identifies individuals on the UNOS waitlist who may achieve equipoise by accepting a xenograft instead of continuing to wait for an allotransplant.

Our findings suggest that achieving equipoise under the current assumptions about the expected longevity of xenografts in initial human trials is unlikely unless the xeno-kidney remains functional for at least two years. Specifically, when the expected xenograft survival (*n*) is less than two years, the model fails to identify sufficiently many patients who would derive greater benefit from a xenotransplant compared to waiting for an allotransplant. At *n* = 2, the capture rates improve, potentially aiding in pinpointing ideal candidates for initial human trials. As *n* increases beyond this point, the models can identify more viable candidates. However, identifying a sufficiently large pool of eligible patients remains critical. This is because other factors such as medical, logistical, and geographic constraints can further limit the number of candidates. For example, patients should be listed at transplant centers equipped to conduct xenotransplantation trials. Although equipoise is not achieved when *n* < 2, it is important to identify patients who might still benefit from participating in early xenotransplant trials. This is particularly relevant given that current expectations for xenograft survival remain below two years and initial trials will involve only small numbers of patients.

Thus, the next best approach is to look for the patients who stand to gain the most from xenotransplantation. To achieve this, we focus on identifying patient groups that are closest to clinical equipoise and examining the factors that define those with the greatest need. This involves evaluating their probability of survival while on the waitlist and their chances of receiving a transplant. Specifically, we analyze survival likelihoods based on their medical and socioeconomic profiles as well as the duration they have already spent waiting for an allotransplant.

Our analysis highlights four patient groups that could potentially benefit the most from xenotransplantation. The first two groups include patients with blood types B or O (both associated with prolonged wait times), who are also diabetic and either older than 65 or age between 50 and 65. The other two groups are subsets of the first two that further restrict attention to those patients who have already waited more than three years.

A trade-off exists in selecting suitable candidates for xenotransplantation trials. On the one hand, patients may die or develop comorbidities that make them ineligible for a transplant as they wait for one because of the prolonged wait times. Therefore, we are primarily looking for those patients who are unlikely to receive an allotransplant quickly. On the other hand, participants should be healthy enough to safely undergo surgery, maintain the xeno-kidney and endure the immunosuppressive regimen following the xenotransplantation. Among the identified groups, younger patients present a stronger case as they are more likely to survive longer, better tolerate complications, and have a higher chance of rejoining the waitlist if the xenograft fails. Notably, diabetic patients of blood type B or O, aged 50 to 65 who have been waiting for more than three years emerge as the most promising group. This group offers a reasonable balance between the potential survival benefit and the physical suitability required for successful xenotransplantation.

Even for these four groups, it is hard to reach equipoise unless the xeno-kidney offers more than two years of survival. Also, the pool of patients in these groups are distributed across more than 200 transplant centers. However, these four cohorts might serve as patient groups that have a combination of a high mortality on the waitlist and poor transplant rates and are common enough at any of the centers that might participate in early trials. They could fit the unusual niche of a being robust enough to withstand the increased immunologic and physiologic demands associated with xenotransplantation and sufficiently disadvantaged by the current allograft allocation system.

Achieving equipoise for xenotransplantation trials could involve offering incentives to patients willing to accept a xenograft, which would make allografts available to others. One potential incentive is allowing patients with a functioning xeno-kidney to remain inactive on the waitlist while still accruing waiting time, a key factor in organ allocation. This approach is easy to implement since it doesn’t require a policy change, but its impact is minimal, increasing the percentage of patients achieving equipoise by only 2.4 percentage points. As a result, it is insufficient to incentivize a significant number of patients across the four identified cohorts. An alternative approach would be to grant patients whose xenografts fail the same priority in the allocation system as former living donors who later develop ESRD. Additionally, we consider a counterfactual scenario where patients receive the highest possible allocation priority after a xeno-kidney failure, effectively placing them at the top of the list. Both strategies lead to a substantial increase in the number of patients achieving clinical equipoise compared to the base case. However, these proposals would require significant changes to existing allocation systems, making widespread acceptance from the transplant community challenging.

Equipoise is often defined in terms of patient survival, which serves as an objective measure of outcomes. However, survival alone does not capture the broader ethical considerations involved in selecting patients for clinical trials. More specifically, equipoise primarily refers to the uncertainty about whether an experimental treatment offers a better risk-benefit balance than the current standard of care [33]. This emphasizes measurable outcomes such as life expectancy but may overlook other factors valued by patients. For example, beyond living longer, some patients may prioritize pain relief, mental clarity, or maintaining physical independence [34]. These quality-of-life considerations should play a role in determining whether a treatment’s benefits justify its risks. For some ESRD patients, the certainty of a xenotransplant may outweigh the wait for an allotransplant, even with lower survival, leading to equipoise. Moreover, informed consent serves as evidence of equipoise by reflecting a patient’s informed decision to accept both the risks and benefits of an experimental treatment [35]. This decision acknowledges uncertainty while linking ethical considerations with a patient-centered approach. Therefore, ESRD patients considering a xenotransplant need to understand survival likelihood, potential complications, and quality-of-life trade-offs.

Beyond survival and traditional clinical outcomes, incorporating health-related quality of life (HRQOL) measures into patient selection frameworks may provide a more comprehensive assessment of trial suitability. Standardized instruments such as the KDQOL-36 or SF-36 can capture domains including symptom burden, functional status, and psychosocial well-being, which are highly relevant to ESRD patients. Integrating these measures into models could help identify patients for whom xenotransplantation offers not only survival gains but also meaningful improvements in daily life. Understanding how xenotransplantation may affect CPRA scores, and in turn opportunities for re-transplantation, will also be critical for accurately assessing long-term patient outcomes. Sensitization following exposure to animal organs could increase antibody levels, making it more difficult for patients to match with compatible human donors in the future. Early trials therefore need to carefully monitor immunological changes, both to guide patient counseling and to inform future allocation policies for those who may return to the waitlist. In parallel, patient advocacy groups may play an important role in trial design and candidate selection by ensuring that patient values, preferences, and lived experiences are adequately represented. Their involvement can help align trial criteria with outcomes that matter most to patients, enhance transparency in the selection process, and facilitate trust in xenotransplant research.

Getting informed consent for experimental xenotransplantation is difficult because of the unknown risks. In addition to the general challenges of explaining complex surgical and immunological risks, xenotransplant candidates face unique uncertainties such as the durability of graft function, the possibility of zoonotic infection, and lifelong requirements for surveillance and registry tracking, which go beyond what is typically addressed in standard models of informed consent [36,37]. Offering incentives, like higher priority for an allotransplant, makes it even harder. Patients with low chances of getting an allotransplant or who feel deprioritized may feel desperate and pressured [38]. The promise of priority might give them hope but could also prevent them from properly weighing the risks. Empirical work with kidney transplant candidates confirms these concerns: patients often express both enthusiasm for the opportunity and concerns about safety, durability, and long-term monitoring, with many noting challenges in weighing hope against risks [39,40]. These complexities are compounded by the fact that xenotransplantation trial participation may involve limitations on privacy [41,42]. Ethical analyses caution that even non-financial incentives, such as allocation priority, can put too much pressure on patients to opt-in, especially those who believe they have low chance at a standard transplant [43,44]. For this reason, many guidelines recommend robust safeguards, including the use of independent patient advocates who can help candidates review their options, ensure understanding, and consider long-term obligations beyond survival benefits [45]. An independent patient advocate could help by advising those overly influenced by incentives and guiding others to make informed choices beyond just survival benefits.

While equipoise is important, finding patients who meet this standard is not enough for a fair xenotransplantation trial. It’s also important to balance risks and benefits and ensure diverse participation [46]. However, given that xenotransplantation trials involve fewer patients than drug trials, achieving fair representation is a challenge. Avoiding a participant pool drawn predominantly from a single nonclinical demographic group can help in this regard. Also, they should reflect the diversity of patients who would ultimately hope to benefit from optimal ESRD treatment.

In summary, the scarcity of donor organs highlights the urgent need for alternative sources, with xenotransplantation emerging as the most viable solution currently available. Using machine learning models, we found that clinical equipoise is difficult to achieve unless xenografts provide survival of more than two years. Incentives could help more patients reach equipoise. Allowing patients with functioning xeno-kidneys to remain inactive on the waitlist while continuing to accrue waiting time has little impact on survival outcomes. On the other hand, granting patients with failed xenografts the same or higher priority as former living donors would significantly increase trial participation. However, it poses ethical and logistical challenges. In addition, we identified patient groups with high mortality on the waitlist and low transplant rates who could make well-informed decisions to pursue xenotransplantation immediately, particularly when prolonged dialysis is their only alternative.

## Supporting information

S1 Appendix Further details of our analysis are provided in S1 Appendix. We provide its table of contents and a brief description of each of its sections below.
The S1 Appendix consists of 12 sections. A brief overview of each section follows next:
S1 Appendix A provides the details of the cohort analysis, focusing on specific groups characterized by low probabilities of survival and receiving an organ. It details the construction of these cohorts and presents survival and time-to-transplant curves, offering a visual representation of the impact of key variables on patient outcomes.S1 Appendix B shows the distribution of patients across the eight transplant centers that might participate in initial xenotransplant clinical trials.S1 Appendix C details the estimation of re-listing probabilities following xeno-kidney failure at both individual and cohort levels. It uses the probability of re-listing after allograft failure as a proxy and discusses the underlying methodologies and assumptions guiding these estimates.S1 Appendix D evaluates the reliability of survival methods through the calculation of mean survival probabilities and mean survival times for all patients and cohorts.S1 Appendix E introduces a recursive approach to study additional incentives , detailing the sequence of events and the decision-making processes faced by patients on the waitlist for transplants upon the failure of the xeno-kidney.S1 Appendix F introduces the Social Deprivation Index (SDI), a tool in our analysis for assessing the impact of socioeconomic status on patient health outcomes. This section details the components of the SDI, which is available at the ZIP code level.S1 Appendix G offers a data description for the patient file, CAND_ KIPA. It explains the demographic and health status variables included, along with providing descriptive statistics for patient registrations and transplants over the years. It also presents descriptive statistics for variables used in our analysis along with data imputation methods.S1 Appendix H lays out the criteria for study population inclusion and exclusion, detailing the data cleaning steps taken. It describes the methodology for handling inactive patients, incorporation of the waiting time variable and the inclusion criteria for multi-listed and re-transplant patients.S1 Appendix I provides implementation details of survival analysis in Section 3.3.1, explaining the rationale behind the use of survival analysis, the preparation of the response variable, and the methodology for forming test and train sets, including sampling and encoding details.S1 Appendix J defines a notion of aggressiveness for transplant centers based on a patient’s likelihood of receiving a transplant. While not among the top three variables identified by RSF and Cox models, it is included in defining highest-need patient groups in Appendix A.S1 Appendix K provides detailed definitions of hit rate and capture rate. It also includes the results of the survival analysis discussed in Sect 3.3.1, which are presented in K.3. Moreover, as a robustness check for the analysis in Sect 3.3.1, we perform classification analysis and present the results in K.4. However, classification methods were uninformative, with a hit rate around 50%, comparable to pure noise.S1 Appendix L provides a robustness check for the analyses in S1 Appendix E which compares the the results obtained from using cohort-level time-to-transplant curves versus those from individual-level curves under the recursive approach for four main cohorts.
(PDF)
