# Supplementary material for: A data-driven analysis of patient selection for xenotransplant human clinical trials
Source: PLoS One. 2025 Dec 1;20(12):e0335767. doi: 10.1371/journal.pone.0335767 (PMC12668613; doi:10.1371/journal.pone.0335767)
Supplement: S1 Appendix — We provide its table of contents and a brief description of each of its sections below. The S1 Appendix consists of 12 sections. A brief overview of each section follows next: S1 Appendix A provides the details of the cohort analysis, focusing on specific groups characterized by low probabilities of survival and receiving an organ. It details the construction of these cohorts and presents survival and time-to-transplant curves, offering a visual representation of the impact of key variables on patient outcomes.S1 Appendix B shows the distribution of patients across the eight transplant centers that might participate in initial xenotransplant clinical trials.S1 Appendix C details the estimation of re-listing probabilities following xeno-kidney failure at both individual and cohort levels. It uses the probability of re-listing after allograft failure as a proxy and discusses the underlying methodologies and assumptions guiding these estimates.S1 Appendix D evaluates the reliability of survival methods through the calculation of mean survival probabilities and mean survival times for all patients and cohorts.S1 Appendix E introduces a recursive approach to study additional incentives , detailing the sequence of events and the decision-making processes faced by patients on the waitlist for transplants upon the failure of the xeno-kidney.S1 Appendix F introduces the Social Deprivation Index (SDI), a tool in our analysis for assessing the impact of socioeconomic status on patient health outcomes. This section details the components of the SDI, which is available at the ZIP code level.S1 Appendix G offers a data description for the patient file, CAND_ KIPA. It explains the demographic and health status variables included, along with providing descriptive statistics for patient registrations and transplants over the years. It also presents descriptive statistics for variables used in our analysis along with data imputation methods.S1 Appendix H lays out the criteria for stu [file pone.0335767.s001.pdf]

Supporting Information: Table of Contents and Summary

A Survival and Time-to-Transplant Curves **3**
A.1 Variable Importance Scores 5

B Distribution of Patients Across Transplant Centers **8**

C Estimation of Relisting Probability **8**
C.1 Individual-level Relisting Probabilities using Classification Model 9
C.2 Cohort-level Relisting Probabilities using Competing Risks Model 11

D Performance of the Survival Models **12**

E An Analysis of Incentives **19**
E.1 A Methodological Approach for Assessing the Incentives: A Recursive Approach to Calculate Patients' Life Expectancy 19
E.2 No Incentive Case (Base Case) 20
E.3 The Keep Inactive Case 21
E.4 Treating Xenotransplant Recipients As If They Were Former Donors . . 21
E.5 Giving Xenotransplant Recipients the Highest Priority on the Waitlist . 21

F The Social Deprivation Index **25**

G Data Description for the Patient File, CAND_KIPA **26**
G.1 Descriptive Statistics of Variables 28
G.2 Data Imputation 29

H Study Population and Exclusion Criteria **29**
H.1 Data Cleaning 29
H.2 Inactive Patients 30
H.3 Waiting Time 33
H.4 Multi-listed & Re-transplant Patients 33

I Implementation Details of Survival Analysis in Section 3.3.1 **36**

J Aggressiveness Analysis of Transplant Centers in Defining Cohorts **36**
J.1 Definition of Marginal Organs 38
J.2 Opt-in Status of Patients and Offer Seen Rate 39
J.3 Acceptance Rate 40

K Confusion Matrices and Evaluation Metrics for Cohort Analysis **41**
K.1 Formal Definitions of Hit Rate and Capture Rate 41
K.2 Summary Tables of Survival Analysis 42
K.3 Results of Survival Analysis 46
K.4 Results of Classification Analysis 46

L Results for Cohort-Level vs. Individual-Level Time-to-Transplant Curves for Re-Transplants: A Robustness Check for S1 Appendix E **71**
L.1 With Random Survival Forest Model 72
L.2 With DeepSurv Model 73
L.3 With Cox Model 74

The S1 Appendix consists of 12 sections. A brief overview of each section follows next:

- S1 Appendix A provides the details of the cohort analysis, focusing on specific groups characterized by low probabilities of survival and receiving an organ. It details the construction of these cohorts and presents survival and time-to-transplant curves, offering a visual representation of the impact of key variables on patient outcomes.
- S1 Appendix B shows the distribution of patients across the eight transplant centers that might participate in initial xenotransplant clinical trials.
- S1 Appendix C details the estimation of re-listing probabilities following xeno-kidney failure at both individual and cohort levels. It uses the probability of re-listing after allograft failure as a proxy and discusses the underlying methodologies and assumptions guiding these estimates.
- S1 Appendix D evaluates the reliability of survival methods through the calculation of mean survival probabilities and mean survival times for all patients and cohorts.
- S1 Appendix E introduces a recursive approach to study additional incentives , detailing the sequence of events and the decision-making processes faced by patients on the waitlist for transplants upon the failure of the xeno-kidney.
- S1 Appendix F introduces the Social Deprivation Index (SDI), a tool in our analysis for assessing the impact of socioeconomic status on patient health outcomes. This section details the components of the SDI, which is available at the ZIP code level.
- S1 Appendix G offers a data description for the patient file, CAND_ KIPA. It explains the demographic and health status variables included, along with providing descriptive statistics for patient registrations and transplants over the years. It also presents descriptive statistics for variables used in our analysis along with data imputation methods.
- S1 Appendix H lays out the criteria for study population inclusion and exclusion, detailing the data cleaning steps taken. It describes the methodology for handling inactive patients, incorporation of the waiting time variable and the inclusion criteria for multi-listed and re-transplant patients.
- S1 Appendix I provides implementation details of survival analysis in Section 3.3.1, explaining the rationale behind the use of survival analysis, the preparation of the response variable, and the methodology for forming test and train sets, including sampling and encoding details.
- S1 Appendix J defines a notion of aggressiveness for transplant centers based on a patient's likelihood of receiving a transplant. While not among the top three variables identified by RSF and Cox models, it is included in defining highest-need patient groups in Appendix A.
- S1 Appendix K provides detailed definitions of hit rate and capture rate. It also includes the results of the survival analysis discussed in Section 3.3.1, which are presented in K.3. Moreover, as a robustness check for the analysis in Section 3.3.1, we perform classification analysis and present the results in K.4. However, classification methods were uninformative, with a hit rate around 50%, comparable to pure noise.

- S1 Appendix L provides a robustness check for the analyses in S1 Appendix E which compares the the results obtained from using cohort-level time-to-transplant curves versus those from individual-level curves under the recursive approach for four main cohorts.

A Survival and Time-to-Transplant Curves

Our analysis first considers all patients. Then, we repeat the same analysis for certain subpopulations of patients, which we refer to as the cohort analysis. In order to determine these subpopulations, we consider how patient characteristics affect survival probabilities and the likelihood of receiving a transplant.

To that end, we obtain variable importance scores and identify the significant variables, see S1 Appendix A.1. We take the union of the sets of the top 3 variables identified by RSF and Cox models for each analysis, see Tables S5 - S8. Note that, DeepSurv model does not provide a way to identify the important variables. Moreover, we add the aggressiveness of the transplant centers and CPRA score, even though they do not appear as the top 3 variables, see S1 Appendix J. This is because a patient's likelihood of receiving a transplant can depend on the transplant center he is registered at. Moreover, high CPRA patients are notoriously difficult to match. In particular, we study the effect of the variables listed in Table S1 on survival probabilities and the probability of receiving a transplant. Below, we will discuss how we use these variables in our cohort analysis.

Table S1. List of patient characteristics that affect survival probabilities and the probability of receiving a transplant.

**Patient Characteristics**
Diabetes status
Blood type
Age at listing
Waiting time
Previous kidney transplant
The aggressiveness of the transplant center
CPRA score

We overlay survival and time-to-transplant curves to study the effect of these variables. We use traditional Kaplan-Meier analysis to generate survival curves, whereas we use the competing risks analysis to generate time-to-transplant curves. This is because being removed from the list after receiving a transplant and removal due to other reasons, such as death, are competing events. By definition, a competing risk is an event that either hinders the observation of the event of interest or modifies the chance that this event occurs. In our analysis, the event of interest is receiving a transplant. If a patient is removed from the list due to another reason, such as death, he can no longer receive a transplant. That is, removal for another reason rules out the possibility of a transplant. That's why receiving a transplant and removal due to other reasons are competing events.

Figs 1-7 show survival and time-to-transplant curves for different sets of patients where blue and purple curves represent survival probabilities and probability of receiving a transplant, respectively.

Fig 1 shows that patients with age 18-49 have higher survival probabilities and higher probabilities of receiving a transplant than older patients. This implies that such patients can be better off by waiting on the list without getting a xeno-organ. However, as age increases, both probabilities decrease significantly. Therefore, patients with age

50-64 or 65+ can benefit from participating in the first human clinical trials. We include them in our cohort analysis.

Fig 2 shows that the effect of diabetes on survival and time-to-transplant is stark. Since having diabetes significantly lowers both probabilities, diabetic patients are viable candidates for the initial xeno trials, and we focus on them for the subpopulations in the cohort analysis.

As seen from Fig 3, patients with blood type 0 or B are less likely to receive a transplant. In the cohort analysis, we only focus on patients with blood type 0 or B as it allows the xenotransplant clinical trial to reach clinical equipoise.

Fig 4 indicates that patients who did not receive a transplant in the first three years are less likely to receive a transplant in the upcoming years. Indeed, the likelihood of receiving a transplant further decreases as patients wait for more than six years. Therefore, we put the patients who wait for more than three years into one group and the rest in another.

Fig 5 shows that patients with a previous transplant are more likely to receive a transplant. We ultimately do not consider them in a separate cohort because of the data size limitation, see Table S3 for further details.

Patients registered at the bottom 1/3 aggressive centers have a slightly lower probability of receiving a transplant; see Fig 6 and S1 Appendix J for the definition and analysis of transplant center aggressiveness. Both sets of patients considered in Fig 6 can be viable candidates for clinical trials. Although there is only a slight difference in aggregate, we explore whether the aggressiveness of the transplant centers matters for certain subpopulations of patients (Cohorts 7-10) in our cohort analysis.

Lastly, Fig 7 shows that patients with CPRA scores over 99.5 are less likely to receive a transplant.

Now, we explain our cohort analysis building on the preceding discussion. When deciding the cohorts, we take into account the data size. One consideration is that in order to have sufficient statistical accuracy, we need to observe sufficiently many events (i.e. deaths) within n years. First, we focus on the patients with CPRA scores over 99.5 separately without further dividing because of the limited number of patients. Using the rest of the variables, we adopt a three-stage approach. In the first stage, we exclude certain subpopulations and focus only on diabetic patients with blood type 0 or B. In the second stage, we exclude patients younger than 50 and consider remaining patients under two age groups, 50-65 and 65+. So far, we have been adding cuts on top of each other and consider their intersection whereas, in the third stage, we bring in variables one at a time due to the data size limitation.

To be more specific, we proceed as follows:

- **Stage 1:** We start with diabetes status which is the most commonly occurring variable in Tables S5 - S8. As seen in Fig 2, there is a significant difference in the diabetic patients, so in our analysis, we focus on the diabetic patients which is a more promising group. Thereafter, we follow through with the groups according to their size as we want to make sure that the cohort size is sufficiently large. Table S2 provides descriptive statistics for the variables which can be used in the cohort analysis. Next, we restrict our attention to the patients with blood type 0 or B since they are less likely to receive a transplant, see Fig 3. Moreover, as age increases, survival probabilities and probability of receiving a transplant decrease considerably, see Fig 1. Patients with age 18-49 have higher survival probabilities and higher probabilities of receiving a transplant, meaning they are better off by waiting on the list instead of participating in clinical trials. Therefore, we only focus on patients with age 50+ and further evaluate them under two age groups as we will discus in the next stage.

- **Stage 2:** In this stage, we divide patients into two subpopulations which are patients with age 50-65 and 65+, following the categorization proposed by UNOS. Since these three variables (diabetes, blood type and age) yield a significant difference and they still form a large group, we focus on their intersection. Notice that after this point, any further cuts will lower the data size too much. Therefore, going forward, we will add one cut at a time, which we refer to as Stage 3. Stage 3.1 and Stage 3.2 represent the third phase for age groups 50-65 and 65+, respectively.
- **Stage 3:** So far, we have two cohorts of interest: (i) diabetic patients with blood type 0/B and age 50-65, and (ii) diabetic patients with blood type 0/B and age 65+. In Stage 3, we bring in additional cuts one at a time without narrowing the data too much. That is, for diabetic patients with blood type 0/B and age 50+, we add cuts based on the waiting time (<3 years or ≥ 3 years) or the aggressiveness of the transplant center (bottom 1/3 or top 1/3) one at a time. Notice that, we don't have sufficiently many patients who had a previous transplant. That's why we do not study them in a separate cohort.

Table S2. Descriptive statistics for variables to be used in cohort analysis.

**Variable**		%		%		%
Diabetes status	Yes	48.3	No	51.7		
Blood type	0/B	69.4	A/AB	30.6		
Age at listing	[18,49]	34.0	[50,64]	45.7	65+	20.3
Waiting time	<3 yrs	71.6	≥ 3 yrs	28.4		
Previous kidney transplant	Yes	1.1	No	98.9		
The aggressiveness of the transplant center	Bottom	19.6	Top	41.5		
CPRA score	[80,94]	3.7	[95,99.5]	2.9	99.5+	2.8

Table S3 provides the size of the data, i.e. the number of patients, and the fraction of patients who die in n years after each step. Tables S4 and 3 show the proposed cohorts.

It is worth noting in Table S2, for the aggressiveness of the transplant centers, "Top" includes the top third of the transplant centers, and it corresponds to 41.5% of the patients. Similarly, "Bottom" includes a third of the centers, and it corresponds to 19.6% of the patients.

Our analysis highlights four key groups likely to benefit from a xeno-kidney among the 11 shown in Table 3. The first group includes diabetic patients over the age of 65 with blood type B or O, while the second group comprises those aged 50 to 65 with the same blood types. Additionally, within these two groups, we further identify subsets of patients who have been on the waiting list for over three years. It appears that these patient groups may benefit from the acceptance of relatively high risk donor organs, highlighting the importance of considering xeno-kidney transplantation for these specific demographics to potentially improve outcomes, see [16].

A.1 Variable Importance Scores

For RSF method, we use the Breiman-Cutler permutation importance score to rank the variables in order of variable importance, see [26]. In the Cox model, we assess feature importance by examining the magnitude of the coefficient. (Recall that the covariates are normalized.) A larger value implies that the feature is more important for generating a prediction. Tables S5 and S6 show the top 10 variables to estimate the survival probabilities in RSF and Cox models, respectively. Similarly, Tables S7 and S8 provide the top-10 variables to estimate the probability of receiving a transplant in RSF and Cox models, respectively.

Table S3. The number of patients and the fraction of patients who die in n years after each step of the cohort analysis. For example, let us consider Stage 3.1. We have 26,888 diabetic patients with blood type 0/B and age 50-65. Then, we add further cuts one at a time. If we restrict our attention to such patients with waiting time less than three years, we are left with 19,119 patients. Instead, if we consider the ones with waiting time more than three years, then we have 7,769 such patients.

	Variable	# of patients			% of patients die in n yrs			
		Total	Train	Test	$n=2$	$n=3$	$n=4$	$n=5$
	- (All patients)	150199	120165	30034	23.5	32.7	39.9	46.3
Stage 1	Diabetic	72583	58076	14507	29.4	40.8	49.5	57.1
Stage 1	Diabetic + Blood type 0/B	49892	39923	9969	29.5	41.2	50.2	58.2
Stage 1	Diabetic + Blood type 0/B + Age 50+	39218	31374	7844	31.7	44.1	53.4	61.8
	Diabetic + Blood type 0/B + Age 50+	39218	31374	7844	31.7	44.1	53.4	61.8
Stage 2	→ Age 50-65	26888	21512	5376	28.1	40.0	49.0	57.7
Stage 2	→ Age 65+	12330	9861	2469	39.3	53.3	63.1	70.9
	Diabetic + Blood type 0/B + Age 50-65	26888	21512	5376	28.1	40.0	49.0	57.7
Stage 3.1	+ Waiting time $<$ 3 yrs	19119	15298	3821	25.1	36.9	46.3	55.3
Stage 3.1	+ Waiting time $\geq$ 3 yrs	7769	6216	1553	35.7	47.6	55.8	63.5
Stage 3.1	+ Top 1/3 tx centers	11154	8921	2233	28.1	39.9	48.8	57.5
Stage 3.1	+ Bottom 1/3 tx centers	5252	4197	1055	28.5	40.7	49.3	57.6
Stage 3.1	+ Previous kidney tx	362	290	72	11.8	16.5	23.1	27.8
	Diabetic + Blood type 0/B + Age 65+	12330	9861	2469	39.3	53.3	63.1	70.9
Stage 3.2	+ Waiting time $<$ 3 yrs	8243	6598	1645	34.5	48.7	59.2	68.0
Stage 3.2	+ Waiting time $\geq$ 3 yrs	4087	3271	816	49.1	62.5	71.0	76.8
Stage 3.2	+ Top 1/3 tx centers	4681	3747	934	40.1	54.6	63.9	71.7
Stage 3.2	+ Bottom 1/3 tx centers	2411	1929	482	40.2	53.1	63.4	70.6
Stage 3.2	+ Previous kidney tx	83	67	16	20.5	25.3	30.1	33.7

Table S4. Variables used in the cohort analysis.

Cohort	Age 50-65	Age 65+	Wait All	Wait $<$ 3 yrs	Wait $\geq$ 3 yrs	Tx center All	Tx center Bottom 1/3	Tx center Top 1/3
1	✓		✓			✓		
2		✓	✓			✓		
3	✓				✓	✓		
4		✓			✓	✓		
5	✓			✓		✓		
6		✓		✓		✓		
7	✓		✓				✓	
8		✓					✓	
9	✓		✓					✓
10		✓						✓

Table S5. Variable importance scores of top 10 variables in RSF to estimate survival probabilities.

Variable	Imp. Score
Age at listing	0.510
Diabetes	0.259
Waiting time	0.065
Albumin	0.060
Source of payment	0.037
Race	0.032
Angina	0.020
Previous kidney transplant	0.019
CPRA score	0.018
Congestive heart failure	0.018

Table S6. Coefficients of top 10 variables in Cox to estimate survival probabilities.

Variable	Coefficient
Diabetes	0.49
Age at listing	0.47
Previous kidney transplant	0.43
Race	0.41
Source of payment	0.23
Angina	0.23
Tobacco use	0.21
Waiting time	0.17
Congestive heart failure	0.15
Albumin	0.12

Table S7. Variable importance scores of top 10 variables in RSF to estimate the probability of receiving a transplant.

Variable	Imp. Score
Waiting time	0.212
Blood type	0.044
Diabetes	0.043
Previous kidney transplant	0.039
Age at listing	0.037
CPRA score	0.034
Aggressiveness of the tx center	0.029
SDI score	0.027
Source of payment	0.014
Albumin	0.013

Table S8. Coefficients of top 10 variables in Cox to estimate the probability of receiving a transplant.

Variable	Coefficient
Waiting time	2.15
Previous kidney transplant	0.56
Blood type	0.28
Diabetes	0.24
Age at listing	0.14
Source of payment	0.18
Aggressiveness of the tx center	0.13
CPRA score	0.10
Albumin	0.06
SDI score	0.06

B Distribution of Patients Across Transplant Centers

Fig S1 displays the sizes of each cohort across the eight transplant centers that might participate in xenotransplant clinical trials (Table S9).

Table S9. List of transplant centers that might participate in xenotransplant clinical trials.

ID	City	Name
7	Birmingham	University of Alabama Hospital
119	Miami	Jackson Memorial Hospital
190	Chicago	University of Chicago Medical Center
298	Boston	Massachusetts General Hospital
313	Baltimore	University of Maryland Medical System
350	Minneapolis	University of Minnesota Medical Center
382	Durham	Duke University Hospital
456	New York	NYU Langone Health

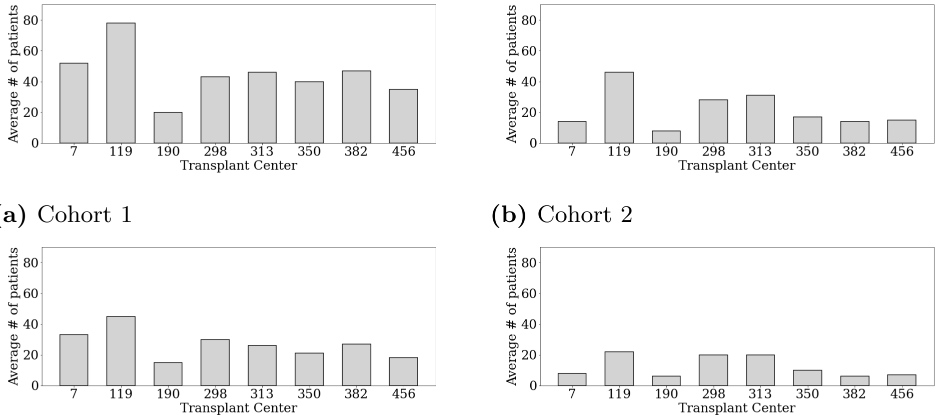

(a) Cohort 1

(b) Cohort 2

(c) Cohort 3

(d) Cohort 4

Fig S1. Average number of patients registered per year to transplant centers that might participate in xenotransplant clinical trials for each cohort.

C Estimation of Relisting Probability

A patient is identified as a candidate for a xenotransplant if her life expectancy is less than that with a xenotransplant. To be more specific, the life expectancy with a xenotransplant is the graft survival of n years plus re-listing probability times the life expectancy upon re-joining the waitlist after the failure of the xeno-kidney. We consider two alternatives to estimate the re-listing probability. Table S10 shows the methodology, input data set to be trained, and the type of the output which indicates whether the relisting probability is computed at the cohort level or on an individual basis for each alternative.

Recall that we calculate the life expectancy under status quo and the life expectancy upon re-joining the list at the individual level using patient characteristics. Similarly, we use the relisting probabilities calculated with Alternative 1 to capture individual variations effectively, excluding the censored observations. As stated in Table S10,

Table S10. Alternative ways to estimate the relisting probability.

Alternative	Methodology	Input data	Output
1	Classification	Patients with reported graft failure	Individual-level
2	Competing risks	Patients who received their first transplant	Cohort-level

Alternative 1 considers only patients with reported graft failure among those who received their first transplant, resulting in a smaller subset of first transplant recipients. Given the smaller dataset, we conduct a robustness check using a competing risks approach (Alternative 2), which retains a larger portion of the data by including all first transplant recipients. However, as a downside of the second approach, we can only obtain relisting probabilities at the cohort level. We observe that the median of the individual-level relisting probabilities are close to the probabilities estimated at the cohort level. We discuss Alternatives 1 and 2 in Sections C.1 and C.2, respectively.

C.1 Individual-level Relisting Probabilities using Classification Model

We estimate the probability of rejoining the waitlist following xenograft failure at the individual level using a classification framework. The outcome is binary: a patient either rejoins the waitlist or does not. If they do not rejoin, we assume they are part of the group that did not survive after xenograft failure. Since direct data on relisting after xenograft failure is unavailable, we approximate this probability using data from allograft failures. That is, we use the probability of re-listing following the failure of an allograft as a proxy. We track whether patients in this setting returned to the waitlist after failure. Patients may not immediately rejoin the waitlist upon graft failure. To account for this, we measure time-to-rejoin starting from the time of graft failure. We assume that if a patient intends to rejoin, they will do so within a time window of r years. To set r, we examine all observed cases of relisting and determine the distribution of time-to-rejoin upon graft failure, see Table S11. The 95th percentile of this distribution is 4.5 years. This means that among all patients who eventually rejoined, 95% did so within 4.5 years of graft failure. We use this as our threshold, assuming that if a patient does not rejoin within r years, they would not have done so even if observed for longer. Censoring arises when the study period ends before a patient's relisting status can be determined. To mitigate this, we restrict our analysis to graft failures that occurred early enough within the study period. Specifically, we only consider failures that happened at least r years before the last observation date (i.e. study end date). This ensures that patients had sufficient time to rejoin if they intended to.

Table S11. Descriptive statistics for time-to-rejoin upon graft failure [year] (uncensored observations).

	0%	5%	10%	20%	30%	40%	50%	60%	70%	80%	90%	95%	99%	100%
	-27.13	-1.87	-0.84	-0.11	0.05	0.22	0.42	0.68	1.03	1.61	2.86	4.53	9.24	23.89

As inputs of the classification model, we consider patient characteristics and the time until graft failure. We include the time until graft failure to the model as a continuous variable. When the xeno-kidney fails after x years, to assess what will happen to them we look at patients who had organ failure within x years. Including the time until graft failure as a continuous variable allows us to train one (common) model regardless of x. As an alternative, one can consider graft failures during the time interval of $[x - 0.5, x)$ and train a model for each $x \in \{0.5, 1, 1.5, 2, 3, 4, 5\}$ separately. Lastly, we set the binary response variable to 1 if the patient rejoined the list (within at most r years), 0 otherwise.

We implement three classification models, namely Gradient Boosting Machine (GBM), Random Forest (RF) and LogisticBoost (LB). As the output, the classification models yield a probability of rejoining the list for the each patient in the test set. In order to assess the reliability of the models, we pick a threshold which maximizes the area under the ROC curve to map probabilities to class labels. Since the input data is imbalanced, we do not set the threshold to 0.5 (the default setting).

Because GBM yields the highest area under the ROC curve, 0.732, we estimate the individual relisting probabilities using GBM and calculate the life expectancies with the xeno-kidney accordingly. We observe that the relisting probability does not depend on n, the expected graft survival, see Tables S12 - S15. Individual-level relisting probabilities increase the number of labeled patients in Cohort 1 and especially in Cohort 3.

Table S12. Distribution of the rejoining probability across n (Cohort 1).

n	0%	5%	10%	20%	30%	40%	50%	60%	70%	80%	90%	95%	99%	100%
0.5	0.09	0.18	0.20	0.23	0.25	0.27	0.30	0.32	0.35	0.38	0.43	0.47	0.54	0.70
1.0	0.09	0.17	0.19	0.22	0.25	0.27	0.29	0.32	0.35	0.38	0.42	0.46	0.54	0.69
1.5	0.09	0.17	0.19	0.22	0.25	0.27	0.29	0.32	0.35	0.38	0.42	0.46	0.54	0.69
2.0	0.09	0.18	0.20	0.23	0.25	0.28	0.30	0.33	0.35	0.39	0.43	0.47	0.55	0.70
3.0	0.10	0.18	0.20	0.23	0.26	0.28	0.31	0.33	0.36	0.39	0.44	0.48	0.55	0.69
4.0	0.09	0.18	0.20	0.24	0.26	0.28	0.31	0.34	0.37	0.40	0.44	0.48	0.56	0.70
5.0	0.09	0.18	0.20	0.23	0.26	0.28	0.31	0.33	0.36	0.40	0.44	0.48	0.56	0.70

Table S13. Distribution of the rejoining probability across n (Cohort 2).

n	0%	5%	10%	20%	30%	40%	50%	60%	70%	80%	90%	95%	99%	100%
0.5	0.07	0.10	0.11	0.12	0.13	0.15	0.16	0.18	0.19	0.21	0.25	0.28	0.34	0.46
1.0	0.07	0.10	0.11	0.12	0.13	0.14	0.16	0.17	0.19	0.21	0.24	0.28	0.34	0.45
1.5	0.07	0.10	0.11	0.12	0.13	0.14	0.16	0.17	0.19	0.21	0.24	0.28	0.34	0.45
2.0	0.07	0.10	0.11	0.12	0.13	0.15	0.16	0.18	0.19	0.21	0.25	0.28	0.35	0.46
3.0	0.07	0.10	0.11	0.13	0.14	0.15	0.16	0.18	0.20	0.22	0.26	0.29	0.35	0.47
4.0	0.07	0.10	0.11	0.13	0.14	0.15	0.17	0.18	0.20	0.22	0.26	0.29	0.35	0.47
5.0	0.07	0.10	0.11	0.12	0.13	0.14	0.16	0.17	0.19	0.21	0.25	0.28	0.34	0.46

Table S14. Distribution of the rejoining probability across n (Cohort 3).

n	0%	5%	10%	20%	30%	40%	50%	60%	70%	80%	90%	95%	99%	100%
0.5	0.12	0.19	0.22	0.24	0.27	0.29	0.32	0.34	0.37	0.40	0.44	0.48	0.55	0.72
1.0	0.12	0.19	0.21	0.24	0.27	0.29	0.31	0.33	0.36	0.39	0.44	0.48	0.55	0.72
1.5	0.12	0.19	0.21	0.24	0.27	0.29	0.31	0.33	0.36	0.39	0.44	0.48	0.55	0.72
2.0	0.12	0.19	0.22	0.25	0.27	0.30	0.32	0.34	0.37	0.40	0.45	0.49	0.56	0.72
3.0	0.12	0.20	0.22	0.25	0.28	0.30	0.32	0.35	0.38	0.41	0.45	0.49	0.57	0.73
4.0	0.12	0.20	0.22	0.25	0.28	0.31	0.33	0.35	0.38	0.41	0.46	0.50	0.57	0.74
5.0	0.12	0.20	0.22	0.25	0.28	0.31	0.33	0.35	0.38	0.41	0.46	0.50	0.57	0.74

Table S15. Distribution of the rejoining probability across n (Cohort 4).

n	0%	5%	10%	20%	30%	40%	50%	60%	70%	80%	90%	95%	99%	100%
0.5	0.07	0.11	0.12	0.13	0.15	0.16	0.18	0.19	0.21	0.23	0.27	0.31	0.36	0.40
1.0	0.07	0.10	0.11	0.13	0.14	0.16	0.17	0.19	0.20	0.23	0.27	0.30	0.36	0.40
1.5	0.07	0.10	0.11	0.13	0.14	0.16	0.17	0.19	0.20	0.23	0.27	0.30	0.36	0.40
2.0	0.07	0.11	0.12	0.13	0.15	0.16	0.18	0.19	0.21	0.23	0.28	0.31	0.37	0.41
3.0	0.08	0.11	0.12	0.14	0.15	0.17	0.18	0.20	0.21	0.24	0.28	0.31	0.37	0.42
4.0	0.08	0.11	0.12	0.14	0.15	0.17	0.18	0.20	0.21	0.24	0.28	0.31	0.37	0.42
5.0	0.07	0.10	0.11	0.13	0.14	0.16	0.17	0.19	0.21	0.23	0.27	0.31	0.37	0.40

C.2 Cohort-level Relisting Probabilities using Competing Risks Model

Table S16 provides a breakdown of patients based on their transplant status. Following the initial transplantation, patients may experience graft failure and sign up for another transplant. After receiving the first transplant, patients can encounter one of the six potential scenarios outlined in Table S17. Table S17 shows the number and fraction of patients in each scenario conditional on receiving the first transplant. By evaluating each scenario separately, we use competing risks analysis to estimate the probability of re-joining the waitlist for another transplant.

Table S16. Partition of patients with respect to their transplant status.

Case	#	%	#	%	#	%
	**All**		**Diabetics, B/O, [50,64)**		**Diabetics, B/O, 65+**	
Did not receive a tx	204,708	43.8	43,579	59.0	18,721	66.4
Received a tx but did not rejoin the list	221,513	47.4	26,227	35.5	8,498	30.2
Received a tx and later rejoined the list	41,586	8.8	4,026	5.5	964	3.42
**Among tx recipients, % rejoined**	-	15.8	-	13.3	-	10.2

Table S17. Possible cases after receiving the first transplant.

Case	Survival status	Graft failure reported?	Rejoined the list?	#	%	#	%	#	%
				**All**		**Diab, B/O, [50,64)**		**Diab, B/O, 65+**	
Case 1	Alive	Reported	No	11,598	4.41	1,245	4.12	302	3.19
Case 2	Alive	Not reported	No	107,437	40.83	11,599	38.34	3,605	38.10
Case 3	Dead	Reported	No	36,115	13.73	4,698	15.53	1,226	12.96
Case 4	Dead	Not reported	No	66,377	25.23	8,686	28.71	3,365	35.56
Case 5	Alive	Reported	Yes	29,636	11.26	3,513	11.61	871	9.21
Case 6	Alive	Not reported	Yes	11,950	4.54	513	1.70	93	0.98

By definition, a competing risk is an event that either hinders the observation of the event of interest or modifies the chance that this event occurs. In our setting, the event of interest is rejoining the waitlist. If a patient dies, he can no longer rejoin the waitlist. That is, death rules out the possibility of rejoining. That's why rejoining the waitlist and death are competing events. In the competing risks model, we eliminate outliers by removing observations that fall beyond the 99th percentile. We consider all times without imposing any restrictions on the registration period for patients. Lastly, the censoring event is being alive after the first transplant.

To be more specific, we define the time until re-joining the waitlist and the status for each case as follows:

- **Case 1 and 2:** Status = 0. Time = Last day of observation - transplantation

date.

- **Case 3 and 4:** Status = 2. Time = Death date - transplantation date.
- **Case 5 and 6:** Status = 1. Time = Rejoin date - transplantation date. Note that Status 1 represents the event of interest.

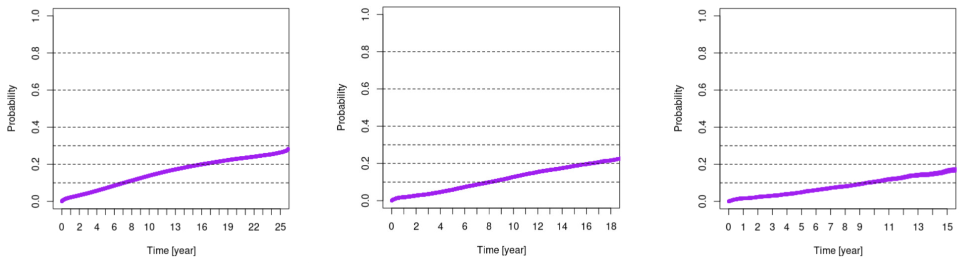

(a) All **(b)** Diabetics, B/O, [50,64) **(c)** Diabetics, B/O, 65+

Fig S2. Time-to-rejoin the waitlist after the first transplant.

In Tables S16, S17 and Fig S2, we provide results for all patients, diabetics with blood type B/O aged [50,65) and diabetics with blood type B/O aged 65+. As a result, we set the probability of joining the waitlist for another transplant to 0.28, 0.24 and 0.18 for all patients, aged [50,65) and 65+, respectively. Note that while estimating the probability of re-joining the waitlist, we do not further group patients with respect to their waiting time due to the data size limitation.

As mentioned earlier, a drawback of this approach is that we can estimate relisting probabilities only at the cohort level. We observe that the median relisting probabilities calculated at the individual level align closely with the probabilities calculated for cohorts.

D Performance of the Survival Models

In this analysis, as a robustness check, we aim to establish that survival methods are reliable. To that end, we check two measures: (i) mean survival probabilities, and (ii) mean survival times. We calculate these measures for all patients and for all cohorts. To be more specific, we apply the following:

1. **Mean survival probabilities:** Using individual survival curves, we obtain the probability of survival at n years for each patient. Then, we take the average across all patients within that cohort at time n. For each cohort, Table S21 shows the fraction of the patients who die or survive in n years, probability of survival using Kaplan-Meier analysis as well as the average survival probabilities under RSF, DeepSurv and Cox models for each n. We overlay the average survival probabilities asscoatiated with each model as well, see Fig S3.
2. **Mean survival times:** Using individual survival curves, we calculate the life expectancy of each patient. Then, we take the average across all patients within that cohort. We compare the mean survival time of the cohort with the life expectancy calculated using Kaplan-Meier survival curve. Table S18 shows the mean survival times for each cohort across all methods.

We observe that the mean survival probabilities and the mean life expectancies are consistent across all methods.

Table S18. Mean survival times in years.

Cohort	KM	RSF	DeepSurv	Cox
All patients	9.129	9.414	9.207	9.222
C1	6.395	6.889	6.624	6.595
C2	4.464	4.851	4.525	4.561
C3	5.823	6.680	6.053	6.109
C4	3.788	4.030	3.865	3.961
C5	6.838	7.110	6.975	6.998
C6	4.744	5.243	4.997	5.040
C7	6.504	6.636	6.365	6.491
C8	4.115	4.096	4.065	4.180
C9	6.543	6.874	6.626	6.633
C10	4.520	4.778	4.655	4.726
C11	7.681	8.251	7.950	8.235

The Kaplan-Meier estimator is a non-parametric method used to estimate the survival function by pooling all patients within a cohort to generate a single survival curve. In contrast, models such as RSF, DeepSurv, and Cox generate individual-level survival curves for each patient, capturing patient-specific risk factors and heterogeneity. To facilitate comparison, we compute cohort-level averages of survival probabilities and survival times. This cohort-level aggregation serves as an approximation of the Kaplan-Meier survival curve, as the Kaplan-Meier method does not account for patient-specific covariates but rather estimates probabilities based on observed event times for the entire cohort. By comparing the Kaplan-Meier estimates with the averaged values from RSF, DeepSurv, and Cox models, we assess the alignment between individual-level survival modeling and cohort-level survival trends, ensuring the robustness of the applied survival methods.

Table S19. Mean survival probabilities.

Cohort	n [year]	% death	% alive	KM	RSF	DeepSurv	Cox
All patients	0.5	6.40	93.60	93.60	93.31	93.62	93.59
	1.0	12.28	87.72	87.70	87.12	87.60	87.54
	1.5	18.19	81.81	81.81	81.13	81.72	81.66
	2.0	23.57	76.43	76.40	75.74	76.41	76.34
	2.5	28.50	71.50	71.50	70.90	71.60	71.53
	3.0	32.73	67.27	67.24	66.55	67.26	67.19
	3.5	36.69	63.31	63.31	62.67	63.39	63.31
	4.0	39.94	60.06	60.04	59.31	60.04	59.96
	4.5	43.29	56.71	56.71	56.10	56.84	56.76
	5.0	46.34	53.66	53.64	53.06	53.81	53.72
C1	0.5	7.33	92.67	92.67	92.27	92.52	92.51
	1.0	14.55	85.45	85.42	84.93	85.28	85.28
	1.5	21.86	78.14	78.14	77.86	78.25	78.25
	2.0	28.29	71.71	71.67	71.07	71.41	71.42
	2.5	34.95	65.05	65.05	65.05	65.34	65.37
	3.0	40.62	59.38	59.36	59.35	59.56	59.60
	3.5	45.65	54.35	54.35	54.55	54.67	54.72
	4.0	50.11	49.89	49.83	50.53	50.57	50.64
	4.5	54.04	45.96	45.96	45.93	45.93	46.01
	5.0	58.20	41.80	41.80	41.90	41.87	41.95
C2	0.5	11.10	88.90	88.90	88.33	88.84	88.84
	1.0	21.06	78.94	78.90	77.90	78.55	78.56
	1.5	31.02	68.98	68.89	68.27	68.93	68.95
	2.0	39.41	60.59	60.55	59.76	60.28	60.33
	2.5	47.19	52.81	52.73	52.58	52.96	53.02
	3.0	53.79	46.21	46.13	46.13	46.36	46.45
	3.5	59.25	40.75	40.75	40.79	40.90	41.01
	4.0	63.55	36.45	36.45	36.37	36.42	36.55
	4.5	67.60	32.40	32.40	32.39	32.37	32.51
	5.0	71.32	28.68	28.64	28.78	28.71	28.88
C3	0.5	10.04	89.96	89.96	90.11	90.46	90.51
	1.0	18.91	81.09	81.04	80.66	81.20	81.28
	1.5	26.85	73.15	73.15	72.71	73.27	73.37
	2.0	34.02	65.98	65.79	65.55	66.06	66.18
	2.5	40.89	59.11	59.11	59.20	59.71	59.83
	3.0	46.69	53.31	53.27	53.77	54.22	54.35
	3.5	51.61	48.39	48.39	49.40	49.82	49.96
	4.0	55.99	44.01	43.96	46.08	46.43	46.58
	4.5	59.36	40.64	40.64	41.89	42.17	42.34
	5.0	62.67	37.33	37.28	38.16	38.35	38.54

Table S20. Mean survival probabilities.

Cohort	n [year]	% death	% alive	KM	RSF	DeepSurv	Cox
C4	0.5	14.66	85.34	85.34	84.05	84.47	84.54
	1.0	26.12	73.88	73.79	70.85	71.30	71.44
	1.5	37.96	62.04	62.04	59.91	60.18	60.40
	2.0	48.16	51.84	51.84	51.59	51.69	51.95
	2.5	56.21	43.79	43.79	44.65	44.60	44.88
	3.0	62.14	37.86	37.77	39.07	38.94	39.23
	3.5	66.99	33.01	33.01	33.76	33.61	33.91
	4.0	69.90	30.10	30.10	30.12	29.92	30.23
	4.5	73.11	26.89	26.80	27.01	26.75	27.07
	5.0	75.92	24.08	24.08	24.47	24.19	24.51
C5	0.5	6.03	93.97	93.97	93.76	93.99	94.00
	1.0	11.95	88.05	88.05	87.62	88.04	88.05
	1.5	18.04	81.96	81.96	80.88	81.45	81.46
	2.0	24.77	75.23	75.20	74.50	75.13	75.15
	2.5	30.62	69.38	69.35	68.49	69.15	69.18
	3.0	36.30	63.70	63.58	62.65	63.30	63.34
	3.5	41.55	58.45	58.45	57.53	58.14	58.19
	4.0	46.26	53.74	53.65	53.08	53.68	53.74
	4.5	51.01	48.99	48.99	48.32	48.90	48.97
	5.0	55.36	44.64	44.61	44.05	44.58	44.66
C6	0.5	8.82	91.18	91.18	91.55	91.99	91.97
	1.0	16.86	83.14	83.07	82.85	83.58	83.54
	1.5	25.40	74.60	74.53	74.07	74.99	74.93
	2.0	33.24	66.76	66.76	65.55	66.45	66.39
	2.5	40.80	59.20	59.13	58.07	58.93	58.87
	3.0	48.50	51.50	51.43	51.15	51.94	51.89
	3.5	54.09	45.91	45.84	45.59	46.23	46.20
	4.0	59.55	40.45	40.38	40.74	41.25	41.26
	4.5	64.66	35.34	35.34	36.20	36.61	36.66
	5.0	69.00	31.00	31.00	31.78	32.09	32.18
C7	0.5	7.70	92.30	92.30	91.39	91.71	91.72
	1.0	14.27	85.73	85.63	83.51	84.10	84.11
	1.5	23.20	76.80	76.80	76.32	76.94	76.97
	2.0	29.67	70.33	70.33	69.45	70.09	70.12
	2.5	35.32	64.68	64.58	62.85	63.48	63.54
	3.0	41.79	58.21	58.01	57.18	57.65	57.72
	3.5	46.20	53.80	53.80	52.31	52.70	52.79
	4.0	49.69	50.31	50.21	48.37	48.65	48.76
	4.5	54.21	45.79	45.79	43.90	44.10	44.25
	5.0	57.80	42.20	42.20	40.20	40.34	40.54

Table S21. Mean survival probabilities.

Cohort	n [year]	% death	% alive	KM	RSF	DeepSurv	Cox
C8	0.5	13.37	86.63	86.63	87.18	87.72	87.80
	1.0	24.78	75.22	75.04	76.10	76.73	76.81
	1.5	34.94	65.06	65.06	66.13	66.63	66.72
	2.0	42.42	57.58	57.58	57.15	57.59	57.66
	2.5	48.31	51.69	51.52	50.34	50.60	50.64
	3.0	56.68	43.32	43.14	43.81	43.85	43.89
	3.5	61.85	38.15	37.97	37.47	37.43	37.51
	4.0	66.67	33.33	33.16	33.62	33.45	33.56
	4.5	70.59	29.41	29.41	29.70	29.42	29.57
	5.0	73.08	26.92	26.56	26.38	26.08	26.27
C9	0.5	7.28	92.72	92.72	92.75	92.97	92.97
	1.0	14.16	85.84	85.80	85.49	85.84	85.84
	1.5	21.24	78.76	78.64	78.76	79.19	79.20
	2.0	27.92	72.08	72.04	71.58	72.03	72.06
	2.5	34.33	65.67	65.59	65.41	65.80	65.86
	3.0	39.82	60.18	60.18	59.49	59.83	59.91
	3.5	45.11	54.89	54.85	54.46	54.76	54.86
	4.0	48.89	51.11	51.07	50.36	50.66	50.78
	4.5	53.66	46.34	46.34	45.82	46.07	46.21
	5.0	57.60	42.40	42.36	41.60	41.82	41.97
C10	0.5	11.67	88.33	88.21	88.23	88.73	88.63
	1.0	21.93	78.07	77.95	78.47	79.17	79.05
	1.5	31.13	68.87	68.63	68.70	69.37	69.24
	2.0	39.15	60.85	60.73	59.86	60.40	60.27
	2.5	46.23	53.77	53.77	52.50	52.91	52.78
	3.0	52.95	47.05	46.93	46.62	46.91	46.79
	3.5	58.02	41.98	41.86	41.87	42.07	41.98
	4.0	62.62	37.38	37.38	38.08	38.23	38.17
	4.5	66.39	33.61	33.61	34.13	34.20	34.17
	5.0	70.87	29.13	29.01	30.75	30.81	30.82
C11	0.5	6.03	93.97	93.97	94.23	94.47	94.53
	1.0	9.65	90.35	90.11	88.78	89.19	89.34
	1.5	14.84	85.16	85.04	83.54	83.87	84.14
	2.0	19.66	80.34	80.22	78.95	79.36	79.77
	2.5	24.73	75.27	75.27	74.26	74.65	75.23
	3.0	29.43	70.57	70.57	69.87	70.14	70.88
	3.5	34.98	65.02	64.90	65.46	65.55	66.45
	4.0	37.64	62.36	62.36	61.38	61.27	62.31
	4.5	42.94	57.06	57.06	57.24	56.99	58.18
	5.0	47.17	52.83	52.71	52.95	52.60	53.95

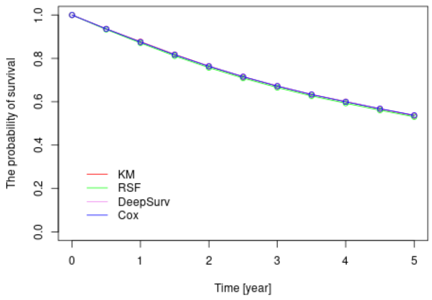

(a) All patients

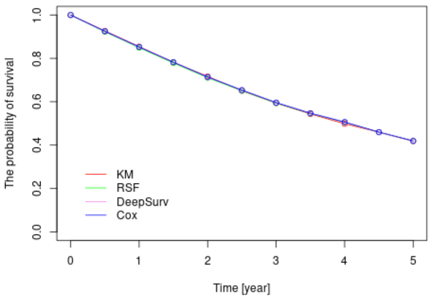

(b) Cohort 1

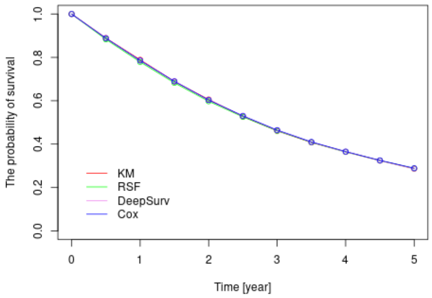

(c) Cohort 2

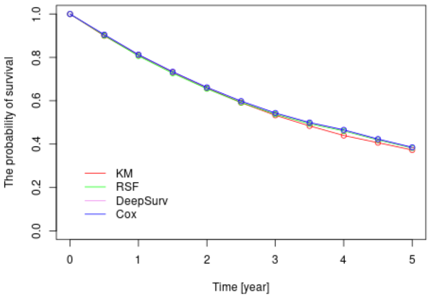

(d) Cohort 3

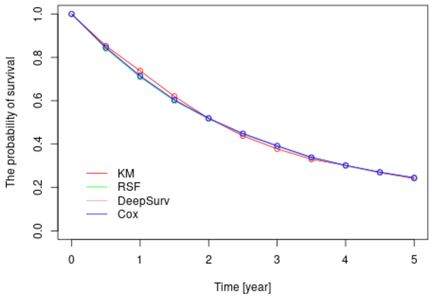

(e) Cohort 4

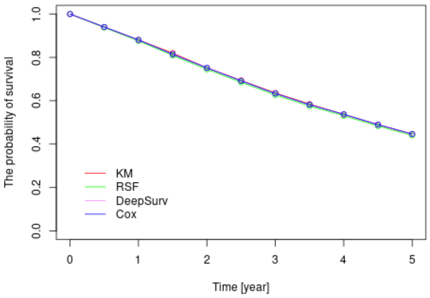

(f) Cohort 5

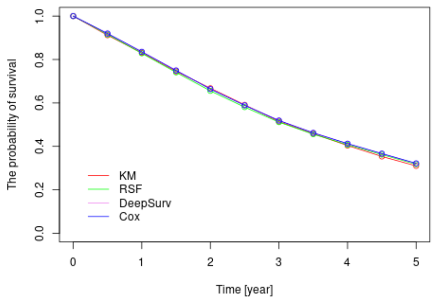

(g) Cohort 6

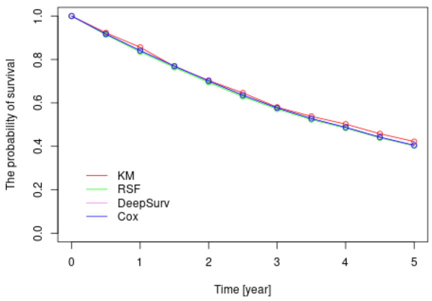

(h) Cohort 7

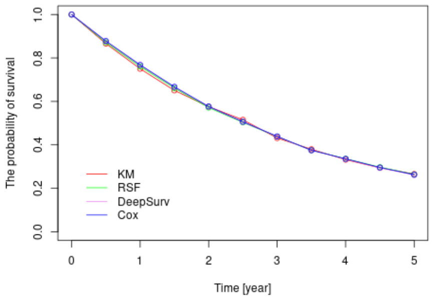

(i) Cohort 8

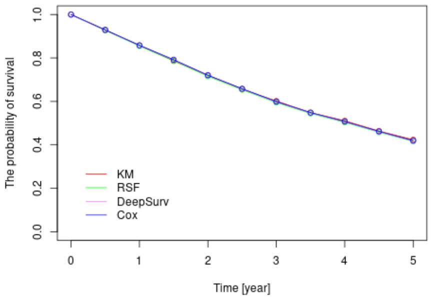

(j) Cohort 9

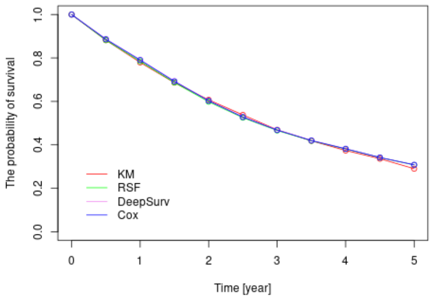

(k) Cohort 10

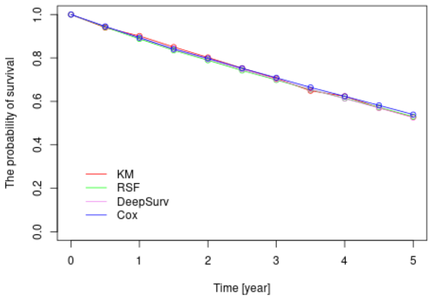

(l) Cohort 11

Fig S3. Mean survival probabilities across survival models.

E An Analysis of Incentives

E.1 A Methodological Approach for Assessing the Incentives: A Recursive Approach to Calculate Patients' Life Expectancy

We assume that xeno-kidney remains functional for n years. Upon the failure of the xeno-kidney, a patient may die or re-join the allotransplant waitlist. In this section, we compute the patient's life expectancy if they rejoin the allotransplant waitlist upon failure of the xeno-kidney using a recursive approach. Conditional on re-joining the waitlist after the xeno-kidney fails, the sequence of events in a period is as follows: At the beginning of period t, the patient receives an immediate utility from waiting/surviving during that period, which is Δt. Then, the patient may die and leave the system. Conditional on being alive at the end of period t, the patient either receives a transplant with some probability and proceeds to the absorbing post-transplant state or she does not receive a transplant and proceeds to the next period, $(t + \Delta t)$.

We assume that the patient can survive on the wait list up to T periods, after which she leaves the system and receives zero utility. In the recursive equation, we set the duration of each period, Δt, to 6 months. In order to calculate T, we restrict our attention to patients who re-joined the list. Conditional on not getting a second transplant, we calculate the time to death after re-joining the waitlist and set T to its 99th percentile. While selecting T, we consider the age groups separately and set T to 21 and 16 years for patients with age [50,65) and 65+, respectively. Two key inputs to our model are the probability of death at the end of each period and the probability of receiving a transplant in each period. We estimate the former at the level of granularity of individual patients using their covariates. However, we estimate the latter at the cohort level using competing risk analysis, see [47], because it allows us to account for the two different types of removal from the waitlist: receiving a transplant or death. In contrast, other survival models we are aware of would treat the death outcome the same as censoring due to other reasons such as reaching the end of the data collection period. Thus, those methods overestimate the probability of receiving a transplant. As a robustness check, we estimated the results both ways and observed that they are generally close to each other though the individual level estimates lead to higher life-expectancy estimates, see S1 Appendix L.

Let $l(t)$ denote the expected life years of the patient starting from period t. We have that

$$\begin{aligned} l(t) &= \Delta t \\ &+ P(\text{death at the end of period } t) \cdot 0 \\ &+ P(\text{still alive at the end of period } t) \cdot \\ &\quad \Big[P(\text{receiving a tx}) \cdot \text{Post-tx} + P(\text{not receiving a tx}) \cdot l(t+\Delta t)\Big] \end{aligned}$$

subject to the boundary condition that $l(T) = 0$.

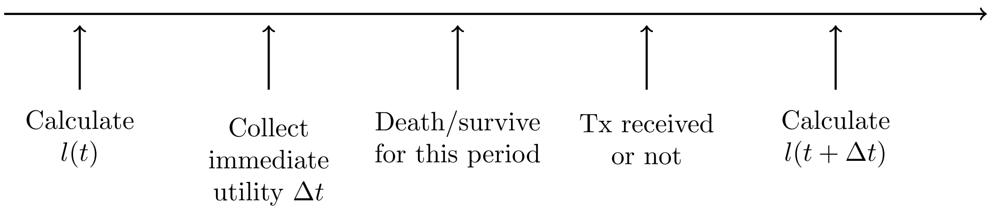

Fig S4. The sequence of events in a period.

Let A denote the life expectancy without the xenotransplant and C denote the life expectancy with the xenotransplant. If $C > A$, then a patient would be willing to accept the xeno-kidney. Using $l(t)$, C can be defined as follows:

$$C = n + P(\text{re-join the waitlist after xeno-kidney fails}) \cdot l(0).$$

E.2 No Incentive Case (Base Case)

Recall that in Section 4, we provide the fraction of the labeled patients under the RSF model in Fig 10 (or see Table S25). This serves as a benchmark to assess the effect of potential incentives. We estimate these fractions using the recursive approach for the purpose of consistency across the analysis for different incentive schemes. Tables S25 - S27 show the fractions derived from the recursive approach.

To be more specific, we define a recursive relationship because keeping patients inactive on the waitlist during the time they have a functioning xeno organ or treating them as if they were former living donors will affect the probability of receiving a second transplant. This is because they continue to accrue priority points. We use time-to-transplant curves to quantify the effect of giving these priority points. Since we do not use time-to-transplant curves in the direct method (see below), recursive approach is the only way to make a comparison between the benchmark case and these incentives. Although the numbers are slightly different as one would expect, we observe that our findings and insights are similar under both approaches.

A Direct Approach: When there are no incentives, there is another and arguably a more direct way to derive the fraction of the labeled patients. In this direct approach, we compare a patient's life expectancy with and without a xenotransplant without using the recursive framework. First, we estimate the patient's expected survival under the status quo, based on their covariates. Then, we compute their life expectancy with a xenotransplant, which consists of the xenograft survival period (n) plus the probability of rejoining the allotransplant waitlist upon xenograft failure, multiplied by the expected survival after rejoining. Unlike the recursive approach, which models period-by-period survival probabilities, the direct approach estimates survival upon re-joining by generating a survival curve based on patient covariates at the time of rejoining and calculating the area under the curve. Finally, a patient is labeled as a candidate if their total expected survival with the xenograft exceeds their expected survival without it. Tables S22 - S24 show the fractions obtained through the direct approach. This is a direct inference from the data, included as a robustness check since we can use both direct and recursive approaches for the no-incentive case. However, the direct approach does not generalize to other settings where we evaluate keeping patients inactive or treating them as former living donors, making the recursive approach necessary for those cases.

Table S22. The fraction of labeled patients under the RSF model (Direct Approach).

n	% Incentivized			
	C1	C2	C3	C4
0.5	0.5	0.0	5.1	0.0
1.0	0.8	0.0	7.8	0.4
1.5	1.5	0.4	10.9	3.1
2.0	3.5	2.2	18.6	10.1
3.0	12.9	19.7	37.6	50.2

Table S23. The fraction of labeled patients under the DeepSurv model (Direct Approach).

n	% Incentivized			
	C1	C2	C3	C4
0.5	0.8	0.0	6.8	0.0
1.0	1.5	0.4	10.7	0.3
1.5	3.0	1.7	17.7	3.0
2.0	6.8	6.2	28.4	12.4
3.0	23.4	31.0	52.9	60.8

Table S24. The fraction of labeled patients under the Cox model (Direct Approach).

n	% Incentivized			
	C1	C2	C3	C4
0.5	1.0	0.5	7.2	0.3
1.0	1.8	1.0	9.3	0.6
1.5	3.1	2.5	13.5	3.0
2.0	5.6	6.0	21.8	12.0
3.0	17.1	28.7	39.6	47.8

Table S25. (RSF) The fraction of the labeled patients - Base case (Recursive Approach).

n	% Labeled			
	C1	C2	C3	C4
0.5	0.7	0.0	1.7	0.2
1.0	1.3	0.2	3.4	1.4
1.5	2.8	1.2	7.0	6.4
2.0	7.0	5.0	14.3	20.4
3.0	24.7	30.5	35.7	60.6

Table S26. (DeepSurv) The fraction of the labeled patients - Base case (Recursive Approach).

n	% Labeled			
	C1	C2	C3	C4
0.5	1.9	0.1	4.4	0.0
1.0	3.3	0.8	7.6	1.0
1.5	6.3	3.0	12.9	5.9
2.0	13.3	9.2	22.5	18.8
3.0	35.3	37.2	46.2	66.5

Table S27. (Cox) The fraction of the labeled patients - Base case (Recursive Approach).

n	% Labeled			
	C1	C2	C3	C4
0.5	1.2	0.2	3.3	0.2
1.0	2.4	0.7	6.1	1.7
1.5	5.0	3.0	11.3	7.1
2.0	10.4	9.0	21.7	20.1
3.0	29.1	36.9	45.7	59.2

E.3 The Keep Inactive Case

We provide results for keeping patients inactive on the list in Tables S28 - S30.

Table S28. (RSF) The fraction of incentivized patients - Keep Inactive.

n	% Incentivized			
	C1	C2	C3	C4
0.5	0.7	0.0	1.7	0.2
1.0	1.4	0.2	3.8	1.7
1.5	3.3	1.2	7.6	6.9
2.0	7.8	5.4	15.7	22.1
3.0	26.8	31.7	38.1	61.7

Table S29. (DeepSurv) The fraction of incentivized patients - Keep Inactive.

n	% Incentivized			
	C1	C2	C3	C4
0.5	2.0	0.1	4.7	0.0
1.0	3.5	0.9	8.0	1.1
1.5	7.0	3.2	14.1	6.8
2.0	14.6	9.5	24.0	19.8
3.0	37.3	38.4	49.0	67.2

Table S30. (Cox) The fraction of incentivized patients - Keep Inactive.

n	% Incentivized			
	C1	C2	C3	C4
0.5	1.3	0.2	3.4	0.2
1.0	2.4	0.8	6.4	1.7
1.5	5.3	3.3	11.9	7.6
2.0	11.1	9.1	22.8	20.9
3.0	30.5	38.0	48.3	60.5

E.4 Treating Xenotransplant Recipients As If They Were Former Donors

We consider a counterfactual scenario in which a patient receives the same priority points as former living donors who develop ESRD upon xenograft failure. In this scenario, they are granted an additional 4 priority points, see Tables S31 - S33.

Table S31. (RSF) The fraction of the incentivized patients - Prior living donor.

n	% Incentivized			
	C1	C2	C3	C4
0.5	3.1	0.5	7.0	3.2
1.0	4.8	1.8	10.6	7.1
1.5	9.0	4.6	17.3	18.0
2.0	16.9	12.5	29.7	38.3
3.0	41.4	43.3	54.1	77.3

Table S32. (DeepSurv) The fraction of the incentivized patients - Prior living donor.

n	% Incentivized			
	C1	C2	C3	C4
0.5	6.2	1.2	12.0	3.0
1.0	10.0	2.9	18.5	7.8
1.5	16.1	7.6	26.1	16.7
2.0	25.4	16.0	38.5	34.5
3.0	49.3	48.9	64.0	78.6

Table S33. (Cox) The fraction of the incentivized patients - Prior living donor.

n	% Incentivized			
	C1	C2	C3	C4
0.5	3.6	0.6	9.2	1.5
1.0	6.0	1.8	13.2	4.7
1.5	10.3	5.2	21.6	12.2
2.0	18.3	13.1	34.2	29.4
3.0	42.0	44.5	59.6	71.5

E.5 Giving Xenotransplant Recipients the Highest Priority on the Waitlist

For this analysis, we take a sample of donors to represent the entire donor population. We do this to streamline our calculations of post-transplant life expectancy for all

potential (patient, donor) pairs, which would otherwise significantly increase the computational time. To be more specific, we consider donors during one month, March 2016.

We provide results in Tables S34 - S45. In these tables, the columns indicate the percentile of all available offers (in terms of quality) made available to returning xenotransplant patients. In the next subsection (E.5.1), we present a set of summary tables that are used in the main text and included here for convenience. These summary tables (S46 - S54) are subsets of the larger tables (S34 - S45) and focus only on three key columns: 50th, 75th, and 90th percentiles.

Table S34. (RSF) The fraction of the incentivized patients (Cohort 1).

n	Percentile of post-transplant life expectancy										
	50%	55%	60%	65%	70%	75%	80%	85%	90%	95%	100%
0.5	5.1	5.4	5.7	6.0	6.3	6.6	7.0	7.3	7.8	8.2	9.6
1.0	8.2	8.7	9.2	9.5	9.9	10.2	10.8	11.2	11.6	12.0	13.8
1.5	13.7	14.0	14.5	14.8	15.4	15.9	16.6	17.3	17.9	18.7	20.8
2.0	23.4	24.2	24.7	25.4	26.0	26.7	27.5	28.2	29.2	30.2	33.0
3.0	48.9	49.7	50.4	51.0	51.5	52.4	53.2	53.8	54.5	55.5	58.6

Table S35. (RSF) The fraction of the incentivized patients (Cohort 2).

n	Percentile of post-transplant life expectancy										
	50%	55%	60%	65%	70%	75%	80%	85%	90%	95%	100%
0.5	1.9	2.0	2.0	2.2	2.2	2.3	2.4	2.4	2.6	2.7	3.0
1.0	3.6	3.6	3.6	3.7	3.9	4.2	4.4	4.6	4.8	5.0	5.4
1.5	7.7	7.9	8.1	8.3	8.7	8.9	9.2	9.3	9.5	10.0	10.5
2.0	17.3	17.7	18.1	18.3	18.6	19.0	19.4	19.7	20.3	20.7	22.0
3.0	49.4	49.6	49.9	50.3	50.8	51.4	52.0	52.5	53.3	53.9	55.8

Table S36. (RSF) The fraction of the incentivized patients (Cohort 3).

n	Percentile of post-transplant life expectancy										
	50%	55%	60%	65%	70%	75%	80%	85%	90%	95%	100%
0.5	11.5	12.3	12.9	13.3	13.5	14.5	14.9	15.4	15.8	16.5	19.1
1.0	16.5	17.3	18.0	19.0	19.9	20.6	21.1	21.4	22.0	23.1	25.5
1.5	26.1	26.7	27.3	27.8	28.6	29.1	29.8	30.4	31.0	32.0	35.1
2.0	37.3	38.3	39.1	39.5	40.0	40.7	41.7	42.4	43.5	44.5	47.6
3.0	60.8	61.7	62.3	62.8	63.4	64.1	64.7	65.7	66.3	66.9	69.9

Table S37. (RSF) The fraction of the incentivized patients (Cohort 4).

n	Percentile of post-transplant life expectancy										
	50%	55%	60%	65%	70%	75%	80%	85%	90%	95%	100%
0.5	6.6	6.9	7.4	7.7	7.9	8.1	8.4	8.6	8.9	9.7	11.5
1.0	14.2	14.5	14.9	15.0	15.5	16.0	16.6	17.1	17.2	17.8	18.9
1.5	26.9	27.9	28.3	29.0	29.7	30.2	30.9	31.4	32.1	33.1	34.9
2.0	46.8	47.2	47.7	48.1	48.8	48.9	49.7	50.3	50.8	51.8	54.0
3.0	82.3	82.4	82.8	83.5	83.9	84.3	84.5	84.8	85.2	86.2	87.2

Table S38. (DeepSurv) The fraction of the incentivized patients (Cohort 1).

n	Percentile of post-transplant life expectancy										
	50%	55%	60%	65%	70%	75%	80%	85%	90%	95%	100%
0.5	9.9	10.7	11.7	12.7	13.5	14.4	16.1	17.9	20.3	23.5	42.0
1.0	14.0	15.2	16.0	17.1	18.7	20.3	21.9	23.9	26.4	29.9	49.6
1.5	19.9	21.3	22.5	24.1	26.1	27.7	29.8	32.2	35.0	39.0	59.2
2.0	30.0	32.0	33.6	35.5	37.7	39.8	42.2	44.6	47.7	51.6	70.1
3.0	52.9	54.7	56.3	58.1	60.0	61.8	64.0	66.3	68.6	71.9	84.9

Table S39. (DeepSurv) The fraction of the incentivized patients (Cohort 2).

n	Percentile of post-transplant life expectancy										
	50%	55%	60%	65%	70%	75%	80%	85%	90%	95%	100%
0.5	2.4	2.7	2.8	3.3	3.6	4.0	4.4	4.9	5.5	6.7	14.9
1.0	4.6	5.0	5.2	5.6	6.2	7.1	8.0	8.5	9.5	11.0	22.9
1.5	9.2	9.9	10.9	11.7	12.4	13.3	14.9	16.2	18.1	20.4	33.7
2.0	19.6	20.5	21.4	22.7	24.0	25.3	26.7	28.7	30.9	33.9	50.3
3.0	50.5	51.8	53.0	54.6	56.4	58.2	60.1	61.8	63.5	65.7	79.3

Table S40. (DeepSurv) The fraction of the incentivized patients (Cohort 3).

n	Percentile of post-transplant life expectancy										
	50%	55%	60%	65%	70%	75%	80%	85%	90%	95%	100%
0.5	18.7	19.9	21.7	23.5	26.1	27.6	29.4	31.5	34.1	38.9	59.9
1.0	25.9	27.4	29.7	31.3	33.6	34.9	36.6	38.8	42.3	46.5	68.0
1.5	34.5	36.2	38.1	40.2	41.9	43.9	46.5	49.4	52.6	57.1	76.6
2.0	47.1	49.0	51.2	52.9	54.9	57.2	59.6	62.5	66.4	70.1	85.1
3.0	70.3	71.8	73.3	74.7	76.3	78.1	80.1	81.9	83.9	86.8	94.1

Table S41. (DeepSurv) The fraction of the incentivized patients (Cohort 4).

n	Percentile of post-transplant life expectancy										
	50%	55%	60%	65%	70%	75%	80%	85%	90%	95%	100%
0.5	4.8	5.4	6.3	7.2	8.1	9.4	10.4	12.4	14.6	17.3	33.3
1.0	11.0	12.6	13.5	14.4	15.4	17.4	18.6	20.8	22.6	25.6	44.9
1.5	21.0	22.7	23.4	25.2	27.1	29.1	32.4	34.5	37.5	41.1	62.6
2.0	40.0	42.1	44.2	46.7	48.4	50.8	53.4	56.3	59.2	63.5	78.5
3.0	83.3	84.1	85.0	85.5	86.2	86.9	87.8	89.0	90.2	92.0	95.9

Table S42. (Cox) The fraction of the incentivized patients (Cohort 1).

n	Percentile of post-transplant life expectancy										
	50%	55%	60%	65%	70%	75%	80%	85%	90%	95%	100%
0.5	7.1	7.6	8.0	8.6	9.1	10.0	10.5	11.2	12.4	13.6	33.8
1.0	10.8	11.2	11.6	12.5	13.3	14.3	15.0	16.1	17.5	19.2	41.8
1.5	16.6	17.2	18.0	19.1	19.9	21.3	22.3	23.6	25.2	27.5	51.9
2.0	27.3	28.0	29.2	30.3	31.5	33.0	34.0	35.7	37.5	39.9	65.4
3.0	51.5	52.6	53.5	55.1	56.3	57.7	59.1	60.6	62.4	64.8	83.8

Table S43. (Cox) The fraction of the incentivized patients (Cohort 2).

n	Percentile of post-transplant life expectancy										
	50%	55%	60%	65%	70%	75%	80%	85%	90%	95%	100%
0.5	2.2	2.3	2.4	2.6	2.7	2.9	3.0	3.5	3.8	4.3	11.6
1.0	4.4	4.7	4.8	5.1	5.3	5.3	5.5	6.2	6.8	7.4	17.8
1.5	9.0	9.1	9.4	9.8	10.2	10.9	11.4	11.9	12.7	13.6	30.0
2.0	18.6	19.2	19.5	20.3	21.2	21.9	22.8	23.8	25.1	26.9	46.7
3.0	51.7	52.3	52.7	53.5	54.6	55.9	56.7	57.6	59.0	60.8	78.7

Table S44. (Cox) The fraction of the incentivized patients (Cohort 3).

n	Percentile of post-transplant life expectancy										
	50%	55%	60%	65%	70%	75%	80%	85%	90%	95%	100%
0.5	15.9	16.6	17.4	18.3	19.2	20.5	21.9	23.5	25.0	27.6	53.5
1.0	22.9	23.8	24.6	26.0	27.4	29.1	30.4	32.1	34.0	36.6	61.7
1.5	33.5	34.7	36.3	37.5	38.2	39.7	40.8	43.0	44.6	46.8	70.8
2.0	47.1	48.1	49.1	50.5	51.7	53.3	55.0	56.3	58.2	60.5	81.4
3.0	70.6	71.2	72.5	73.4	74.2	75.0	76.2	78.2	79.4	81.1	92.2

Table S45. (Cox) The fraction of the incentivized patients (Cohort 4).

n	Percentile of post-transplant life expectancy										
	50%	55%	60%	65%	70%	75%	80%	85%	90%	95%	100%
0.5	5.0	5.5	5.8	5.8	6.2	6.8	7.5	8.3	8.7	9.9	26.2
1.0	10.3	10.8	11.1	11.7	12.4	13.3	13.8	14.6	15.5	16.8	40.7
1.5	21.4	22.2	22.5	24.3	25.3	26.2	27.0	28.4	30.3	32.1	60.1
2.0	42.1	42.5	43.9	45.5	46.5	47.7	48.7	50.7	52.1	54.6	78.2
3.0	81.7	82.0	82.6	83.5	84.4	85.0	85.4	86.9	87.6	89.0	95.6

E.5.1 Summary Tables

We provide summary tables for 50th, 75th, and 90th percentiles in Tables S46 - S54. This is provided solely for convenience, as the complete information is contained in the full tables (S34 - S45).

Table S46. (RSF) The fraction of incentivized patients (50th percentile of post-transplant life expectancy).

n	C1	C2	C3	C4
0.5	5.1	1.9	11.5	6.6
1.0	8.2	3.6	16.5	14.2
1.5	13.7	7.7	26.1	26.9
2.0	23.4	17.3	37.3	46.8
3.0	48.9	49.4	60.8	82.3

Table S47. (RSF) The fraction of incentivized patients (75th percentile of post-transplant life expectancy).

n	C1	C2	C3	C4
0.5	6.6	2.3	14.5	8.1
1.0	10.2	4.2	20.6	16.0
1.5	15.9	8.9	29.1	30.2
2.0	26.7	19.0	40.7	48.9
3.0	52.4	51.4	64.1	84.3

Table S48. (RSF) The fraction of incentivized patients (90th percentile of post-transplant life expectancy).

n	C1	C2	C3	C4
0.5	7.8	2.6	15.8	8.9
1.0	11.6	4.8	22.0	17.2
1.5	17.9	9.5	31.0	32.1
2.0	29.2	20.3	43.5	50.8
3.0	54.5	53.3	66.3	85.2

Table S49. (DeepSurv) The fraction of incentivized patients (50th percentile of post-transplant life expectancy).

n	C1	C2	C3	C4
0.5	9.9	2.4	18.7	4.8
1.0	14.0	4.6	25.9	11.0
1.5	19.9	9.2	34.5	21.0
2.0	30.0	19.6	47.1	40.0
3.0	52.9	50.5	70.3	83.3

Table S50. (DeepSurv) The fraction of incentivized patients (75th percentile of post-transplant life expectancy).

n	C1	C2	C3	C4
0.5	14.4	4.0	27.6	9.4
1.0	20.3	7.1	34.9	17.4
1.5	27.7	13.3	43.9	29.1
2.0	39.8	25.3	57.2	50.8
3.0	61.8	58.2	78.1	86.9

Table S51. (DeepSurv) The fraction of incentivized patients (90th percentile of post-transplant life expectancy).

n	C1	C2	C3	C4
0.5	20.3	5.5	34.1	14.6
1.0	26.4	9.5	42.3	22.6
1.5	35.0	18.1	52.6	37.5
2.0	47.7	30.9	66.4	59.2
3.0	68.6	63.5	83.9	90.2

Table S52. (Cox) The fraction of incentivized patients (50th percentile of post-transplant life expectancy).

n	C1	C2	C3	C4
0.5	7.1	2.2	15.9	5.0
1.0	10.8	4.4	22.9	10.3
1.5	16.6	9.0	33.5	21.4
2.0	27.3	18.6	47.1	42.1
3.0	51.5	51.7	70.6	81.7

Table S53. (Cox) The fraction of incentivized patients (75th percentile of post-transplant life expectancy).

n	C1	C2	C3	C4
0.5	10.0	2.9	20.5	6.8
1.0	14.3	5.3	29.1	13.3
1.5	21.3	10.9	39.7	26.2
2.0	33.0	21.9	53.3	47.7
3.0	57.7	55.9	75.0	85.0

Table S54. (Cox) The fraction of incentivized patients (90th percentile of post-transplant life expectancy).

n	C1	C2	C3	C4
0.5	12.4	3.8	25.0	8.7
1.0	17.5	6.8	34.0	15.5
1.5	25.2	12.7	44.6	30.3
2.0	37.5	25.1	58.2	52.1
3.0	62.4	59.0	79.4	87.6

F The Social Deprivation Index

The Social Deprivation Index (SDI) was initially developed by Butler et al. (2012) and updated by the Robert Graham Center. The SDI is a composite measure of seven demographic characteristics collected in the American Community Survey (ACS). The demographic characteristics are provided in Table S55.

Table S55. List of characteristics used in the Social Deprivation Index.

**Domain**	**Variable**
Income	Percent population less than 100% FPL
Education	Percent population $\geq$ 25 years with $<$ 12 years of education
Employment	Percent non-employed
Housing	Percent population living in renter-occupied housing units
	Percent population living in crowded housing units
Household Characteristics	Percent single-parent households with dependents $<$ 18 years
Transportation	Percent population with no car

G Data Description for the Patient File, CAND_KIPA

The recipient file, CAND_KIPA, contains information that pertains to transplant candidates on the OPTN waitlist who were registered for kidney, pancreas, or kidney/pancreas. There are two identifiers, **PERS_ID** and **PX_ID**. The first one is unique for each person whereas the latter one is generated every time a patient is registered in a transplant center. **WL_ORG** indicates the waitlist to which the patient registered (KI=kidney, PA=pancreas, KP=kidney/pancreas).

Upon registration to a waitlist, each candidate is assigned a waitlist id and the listing date is recorded as **CAN_LISTING_DT**. However, a candidate might be active or inactive on the kidney transplant waiting list from time to time. Being active means that a candidate can be called in for transplantation at any time. **CAN_ACTIVATE_DT** denotes the date at which the patient is activated. If a patient is inactive, then it corresponds to a state at which the candidate is not eligible for a transplantation but keeps accumulating priority points. This may be due to a variety of reasons including health issues, financial problems or personal reasons. The recipient file includes the first and the last date of active/inactive status, **CAN_INIT_ACT_STAT_DT** and **CAN_LAST_ACT_STAT_DT**, whereas the STATHIST_KIPA file provides the intermediate activation and inactivation dates. In addition to these dates, **REC_TX_DT** shows the transplantation date and **DON_TY** corresponds to the donor type (C=cadaveric, L=living), if any. Lastly, there are four fields related to death date. In our analyses, we take the earliest among them:

- **CAN_DEATH_DT**: If removed due to Death, date of death
- **PERS_OPTN_DEATH_DT**: OPTN Death date by unique person
- **PERS_RESTRICT_DEATH_DT**: Restricted death date
- **PERS_SSA_DEATH_DT**: Death date determined from SSA database

Using these identifiers and dates, we calculate the number of new candidates and wait list registrations as well as deceased and living donor transplantations in each year. Table S56 highlights the imbalance in supply and demand of organs. Moreover, Table S57 shows the number of primary and repeat transplants for each donor type.

The recipient data has a number of fields that provide information on the dialysis status of patients. **CAN_ON_DIAL** indicates if a patient is currently on dialysis. If so, **CAN_DIAL_DT** shows the date candidate went on dialysis. The type of dialysis, Hemodialysis or Peritoneal Dialysis, can be learned from the variable **CAN_DIAL**. This can be important to assess the quality of life.

The data set includes the following demographic variables:

- **CAN_AGE_AT_LISTING**: Age at listing
- **CAN_EDUCATION**: Patient's educational status
- **CAN_ETHNICITY_SRTR**: Ethnicity (Latino, Non-Latino)
- **CAN_RACE**: Race (Asian, Black, Multiracial, Native American, Pacific Islander, White)
- **CAN_GENDER**: Gender
- **CAN_PRIMARY_PAY**: Source of payment (Private insurance, Public insurance - Medicaid/Medicare)

Table S56. The number of registrations and transplants in each year.

Year	# of New Candidates	# of New Registrations	# of Deceased Donor Txs	# of Living Donor Txs
2018	38,221	39,908	14,701	6,372
2017	35,010	36,553	14,002	5,747
2016	34,876	36,319	13,408	5,552
2015	34,453	35,969	12,234	5,550
2014	35,510	37,046	11,546	5,447
2013	35,765	37,354	11,135	5,646
2012	34,094	35,605	10,839	5,530
2011	32,890	34,244	11,014	5,667
2010	33,616	34,895	10,582	6,139
2009	32,774	34,089	10,385	6,243
2008	31,733	33,051	10,490	5,831
2007	31,576	32,859	10,526	5,911
2006	30,233	31,500	10,576	6,273
2005	28,146	29,139	9,831	6,408
2004	26,279	27,123	9,248	6,462
2003	23,705	24,409	8,564	6,309
2002	22,679	23,483	8,452	6,079
2001	21,573	22,333	8,147	5,850
2000	21,415	22,285	8,063	5,366
1999	20,227	21,000	8,051	4,701
1998	19,475	20,171	8,040	4,411
1997	18,403	19,049	7,789	3,929
1996	17,729	18,327	7,745	3,678
1995	17,261	17,884	7,691	3,388

- **CAN_WORK_INCOME**: Patient's employment status

The following variables define the health status of patients:

- **CAN_ABO**: Patient's blood type (A,B,AB,0)
- **CAN_BMI**: Body mass index (BMI)
- **CAN_DIAB**: Diabetes type (Type 1, Type 2)
- **CAN_DGN**: Primary diagnosis
- **CAN_ACPT_HBC_POS**: Accept an Hepatitis B Core Antibody Positive Donor? (Y,N)
- **CAN_ACPT_HCV_POS**: Accept an HCV Positive donor? (Y,N)
- **CAN_MALIG**: Previous malignancy
- **CAN_TOT_ALBUMIN**: Total serum albumin
- **CAN_PREV_XX**: Previous transplant where XX stands for the organ type
- **CAN_ANGINA_CAD**: Angina/Coronary artery disease
- **CAN_CEREB_VASC**: Symptomatic cerebrovascular disease
- **CAN_DRUG_TREAT_COPD**: Drug treated COPD

Table S57. Breakdown of transplants. D and L represent deceased and living donors, respectively.

Year	# of D Txs (Total)	# of D Txs (Primary)	# of D Txs (Repeat)	# of L Txs (Total)	# of L Txs (Primary)	# of L Txs (Repeat)
2018	14,701	13,081	1,620	6,372	5,754	618
2017	14,002	12,242	1,760	5,747	5,200	547
2016	13,408	11,579	1,829	5,552	4,979	573
2015	12,234	10,398	1,836	5,550	5,012	538
2014	11,546	10,112	1,434	5,447	4,849	598
2013	11,135	9,790	1,345	5,646	5,023	623
2012	10,839	9,404	1,435	5,530	4,945	585
2011	11,014	9,670	1,344	5,667	5,077	590
2010	10,582	9,272	1,310	6,139	5,495	644
2009	10,385	9,087	1,298	6,243	5,559	684
2008	10,490	9,299	1,191	5,831	5,228	603
2007	10,526	9,274	1,252	5,911	5,316	595
2006	10,576	9,220	1,356	6,273	5,610	663
2005	9,831	8,587	1,244	6,408	5,703	705
2004	9,248	8,005	1,243	6,462	5,784	678
2003	8,564	7,477	1,087	6,309	5,605	704
2002	8,452	7,352	1,100	6,079	5,467	612
2001	8,147	7,060	1,087	5,850	5,281	569
2000	8,063	6,911	1,152	5,366	4,902	464
1999	8,051	7,021	1,030	4,701	4,310	391
1998	8,040	6,969	1,071	4,411	4,051	360
1997	7,789	6,748	1,041	3,929	3,638	291
1996	7,745	6,730	1,015	3,678	3,405	273
1995	7,691	6,664	1,027	3,388	3,119	269

- **CAN_DRUG_TREAT_HYPERTEN**: Drug treated systemic hypertension
- **CAN_PERIPH_VASC**: Symptomatic peripheral vascular disease
- **CANHX_CPRA**: cPRA score
- **DSA**: Donation service Area (58 DSAs)
- **CAN_FUNCTN_STAT** Patient's functional status which is relevant to frailty.

G.1 Descriptive Statistics of Variables

Tables S58 and S59 provide descriptive statistics for numeric and binary variables, respectively. We discuss the imputation of missing variables in S1 Appendix G.2.

Table S58. Descriptive statistics for numeric variables.

Variable	Mean	Median
Age at listing	52.7	54
BMI	28.5	28
cPRA	0.2	0
SDI score	60.2	65
Total serum albumin	3.7	3.8

Table S59. Descriptive statistics for binary variables.

Variable	Yes (%)	No (%)
Accept an HBC positive donor?	61.8	38.2
Accept an HCV positive donor?	10.9	89.1
Angina	92.3	7.7
Candidate on dialysis	82.0	18.0
Diabetes	49.6	50.4
Drug treated COPD	3.4	96.6
Drug treated systemic hypertension	57.8	42.2
Employment status (Employed)	31.3	68.7
Ethnicity (Latino)	17.6	82.4
Gender (Female)	39.0	61.0
Previous malignancy	5.9	94.1
Previous transplant	3.2	96.8
Symptomatic cerebrovascular disease	6.0	94.0
Symptomatic peripheral vascular disease	12.3	87.7
AIDS	0.4	99.6
Alcohol dependence	1.2	98.8
Atherosclerotic heart disease (ASHD)	6.2	93.8
Cancer	2.9	97.1
Cardiac arrest	0.1	99.9
Cardiac dysrhythmia	0.7	99.3
Congestive heart failure	14.9	85.1
Drug dependence	0.7	99.3
HIV positive status	0.5	99.5
Ischemic heart disease (IHD)	3.5	96.5
Myocardial infarction	1.2	98.8
Non-renal congenital abnormality	0.3	99.7
Other cardiac disease	6.9	93.1
Pericarditis	0.3	99.7
Tobacco use	4.4	95.6
Toxic nephropathy	0.3	99.7

G.2 Data Imputation

For continuous variables, we replace all occurrences of missing values of a variable with the median value. For categorical clinical variables, we assume the best possible scenario if a value is missing. For example, if the diabetes status of a patient is unknown, then we assume that the patient does not have diabetes. For the rest of the categorical variables (education, ethnicity, race and the employment status), we define a new category for “unknown” values.

H Study Population and Exclusion Criteria

H.1 Data Cleaning

The SRTR data contains information on all patients from October 1987 until September 1, 2021. However, we restrict our attention to adult patients who are registered at the kidney transplant waitlist after 1995 since the data availability is limited before 1995. We exclude entries whose blood type or listing center information is missing. We also exclude living donor recipients, patients who received three or more transplants, and the ones who are listed at four or more different Donation Service Areas (DSAs). Table S60

shows the data cleaning steps, the number of patients, and waitlist registrations after each step. We discuss the inclusion criteria for the multi-listed and re-transplant patients in S1 Appendix H.4.

Table S60. Data cleaning steps.

Data Cleaning Steps	# of patients	# of entries
Entire set of patients	772,658	1,032,335
Eliminate patients who were registered before 1995	677,230<br>(-95,428)	877,190<br>(-155,145)
Eliminate pancreas and kidney-pancreas patients	635,629<br>(-41,601)	800,858<br>(-76,332)
Eliminate entries if the blood type is missing	635,612<br>(-17)	800,835<br>(-23)
Eliminate pediatric patients	616,321<br>(-19,291)	772,772<br>(-28,063)
Eliminate living donor organ recipients	478,146<br>(-138,175)	587,706<br>(-185,066)
Eliminate entries without a proper listing center	476,915<br>(-1,231)	585,798<br>(-1,908)
Eliminate patients who received 3 or more transplants	476,623<br>(-292)	584,685<br>(-1,113)
Eliminate patients who are listed at 4 or more different DSAs	475,526<br>(-1,097)	579,431<br>(-5,254)

H.2 Inactive Patients

After registration, patients' activation status on the waitlist might change over time. If a patient becomes inactive, then it corresponds to a state in which the candidate is not eligible for transplantation but keeps accumulating priority points. This may be due to a variety of reasons including health issues, financial problems, or personal reasons. There might be temporary changes in the status for short durations as well. For example, a patient might get the flu and become ineligible for a transplant for a week/month. We exclude patients (i) who never get activated, (ii) whose activation status change frequently, and (iii) who stay inactive on the list for the majority of the time; see below for details.

Patients' activation status might change over time as they wait on the list. We analyze how it changes and define a notion of essential inactivity to exclude such patients from our analysis. We use the STATHIST_KIPA file provided under the SRTR data set to check the active/inactive status of patients. This file records characteristics that may change as patients wait on the list, such as active/inactive status. Each record in this file is associated with a time at which the candidate becomes active or inactive.

When a patient becomes inactive, it can be due to a variety of reasons such as health issues, financial problems, or personal reasons. Table S61 lists possible reasons for being inactive, Fig S5 shows the frequency of these reasons.

Table S61. Reasons for inactive status.

Code	Description
1	Candidate cannot be contacted
2	Candidate choice
3	Candidate work-up incomplete
4	Insurance issues
5	Medical non-compliance
6	Inappropriate substance use
7	Temporarily too sick
8	Temporarily too well
9	Weight currently inappropriate for transplant
10	TX'ed - removal pending UNET data correction
11	Inactivation due to VAD implantation and/or VAD complication
12	TX Pending
13	Physician/Surgeon unavailable
14	Candidate for living donor transplant only
15	Administrative: Waiting time/ped-adult adjustment

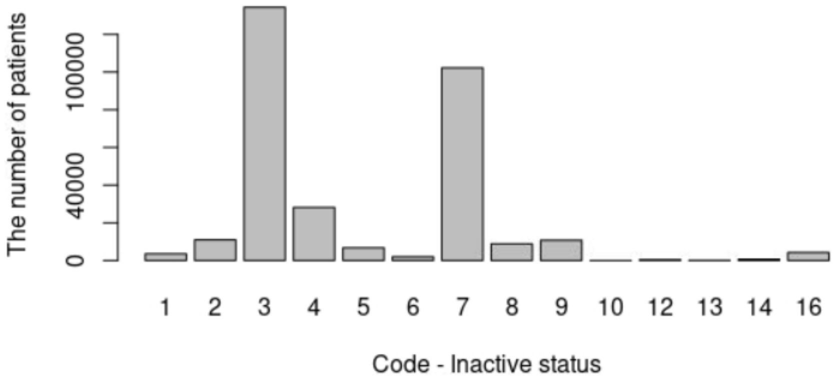

Fig S5. The frequency of the reasons for inactive status.

We classify the changes in patients' activation status into six categories as shown in Table S62. On the one hand, a patient can always be active which we refer to as Case 1. That is, the patient gets activated upon registration and deactivated once he is removed from the list. On the other hand, a patient can always be inactive, see Case 2. Moreover, there are patients that stay active without having any inactive periods in between. We further evaluate such patients under three subgroups as in Cases 3-5. Lastly, in Case 6, we consider patients who have multiple non-consecutive active periods. In other words, these patients' activation statuses follow a "zigzag pattern" with multiple active periods separated by inactive periods. Table S62 includes a brief explanation, status figure, and the percentage of patients for all six cases.

There might be temporary changes in the status for short periods of time. For example, a patient might get the flu and become ineligible for a transplant for a week/month. Therefore, we consider an analysis that ignores such events with short durations. Table S63 shows the percentage of patients in each category when we exclude the short events up to 1 week, 1 month, 3 months, and 6 months. Here, the column with "0" represents the base case where we do not exclude any entries.

Table S63 shows that as we increase the duration of the short events that we are excluding, the percentage of patients with the zigzag pattern decreases. In our analysis,

Table S62. Possible cases for active/inactive status of patients. Green and red blocks represent active and inactive periods, respectively. Green and red blocks are not scaled. In Case 6, it is possible to have more than two green blocks, i.e. active periods.

Case	Explanation	Status	% of patients
1	Always active		45.8
2	Always inactive		8.1
3	Active at listing, becomes and stays inactive		15.8
4	Inactive at listing, becomes and stays active		6.7
5	Inactive-Active-Inactive		2.7
6	Zigzag pattern (multiple changes)		20.9

Table S63. Possible cases for active/inactive status of patients when short events are excluded.

Case	Explanation	Status	% of patients				
			**0**	**1w**	**1m**	**3m**	**6m**
1	Always active		45.8	47.5	51.0	57.1	63.1
2	Always inactive		8.1	8.7	9.8	11.4	13.2
3	Active at listing, becomes & stays inactive		15.8	15.7	16.3	16.8	15.3
4	Inactive at listing, becomes & stays active		6.7	6.7	6.0	4.6	3.2
5	Inactive-Active-Inactive		2.7	2.7	2.6	2.0	1.3
6	Zigzag pattern (multiple changes)		20.9	18.6	14.4	8.1	3.8

we exclude short events up to a month.

To be specific, we exclude patients in Case 2 because they are never active. We also classify two subgroups of patients in Case 6 (of Table S63) as essentially inactive and exclude them from our analysis:

- **Subgroup 1:** Those patients in Case 6 who are activated more than 3 times.
- **Subgroup 2:** Those patients in Case 6 who are inactive more than 70% of their time on the list.

Figs S6 and S7 show the relative frequencies of the patients in these groups (among all patients in Case 6).

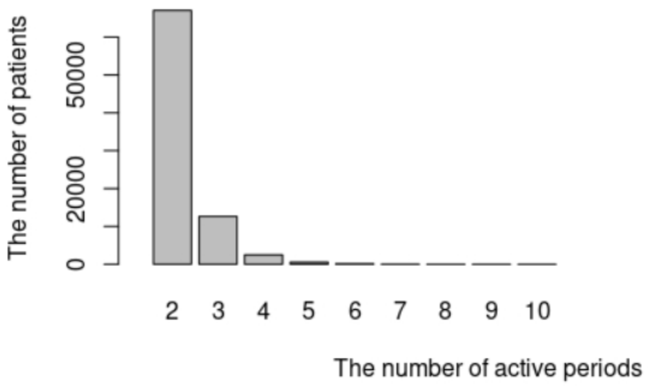

Fig S6. The number of active periods for patients in Case 6.

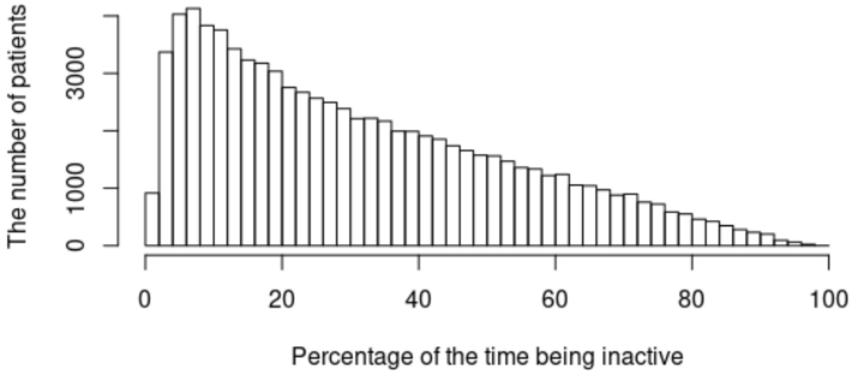

Fig S7. The percentage of time being inactive for patients in Case 6.

H.3 Waiting Time

In order to incorporate waiting time into the analysis, we pick a particular date, take a "snapshot" of the waitlist, and consider patients who are waiting on the list on that date. This allows us to include waiting time as a continuous variable. In our analyses, we consider multiple snapshot dates which are January 15th of 2000, 2005, 2010, and 2015 to increase the data size.

H.4 Multi-listed & Re-transplant Patients

We outline the data cleaning steps in S1 Appendix H.1. After applying these steps, there is a difference between the number of patients and the number of entries in the dataset due to multi-listed and re-transplant patients. In this section, we clarify the definitions of multi-listed and re-transplant patients and discuss the criteria for their inclusion in our analysis.

After data cleaning, we end up with 475,526 patients and 579,431 waitlist entries, indicating multiple waitlist entries associated with the same patient. The difference between the number of patients and waitlist entries are due to the following reasons:

1. A patient might be registered at two or more transplant centers at different DSAs. We call them *multi-listed* patients.

2. A patient might be listed in the same DSA multiple times.

3. A patient might have received two or more transplants after experiencing graft failure(s). We define them as *re-transplant* patients.

There is some overlap between these categories. For example, a re-transplant patient might also be multi-listed. First, we investigate the fraction of patients for each number of transplants received, see Table S64. We observe that re-transplant patients correspond to 1.8% of the entire population.

Table S64. The frequency in the number of transplants.

Number of Transplants	Number of Patients	Percentage of Patients	Cumulative Percentage
0	247,286	52.0	52.0
1	219,474	46.2	98.2
2	8,766	1.8	100.0

Table S65 shows the fraction of multi-listed patients with respect to the number of transplants received. For example, there are 8,766 patients who received two transplants, and %28.5 of them, 2,495 patients, are multi-listed. We observe higher multiple-listing percentages among the patients who receive multiple transplants. Multiple listing appears to increase the chance of receiving an organ offer.

Table S65. The fraction of multi-listed patients with respect to the number of transplants.

Number of Transplants	Number of Patients	Number of Multi-listed Patients	Percentage of Multi-listed Patients
0	247,286	15,147	6.1
1	219,474	31,465	14.3
2	8,766	2,495	28.5

Lastly, we examine how many listings each patient has, see Table S66. We see that 89.7% of patients are registered to only one DSA.

Table S66. The frequency in the number of listings at different DSAs.

Number of Listings at Different DSAs	Number of Patients	Percentage of Patients	Cumulative Percentage
1	426,419	89.7	89.7
2	43,829	9.2	98.9
3	5,278	1.1	100.0

We include multi-listed patients in our analysis as follows:

1. **Case 1:** If a patient received a transplant, we keep the entry at which the patient received a transplant.
2. **Case 2:** For the re-transplant patients, we treat each entry at which the patient received a transplant as a different patient, but incorporate the fact that they already received a transplant in our analysis.
3. **Case 3:** If a patient did not receive any transplant, then we keep the entry with the earliest listing date and for the DSA, we pick the one at which the patient has the highest chance of receiving a kidney transplant. For example, if a patient is listed both in California and Tennessee, then we consider Tennessee as the DSA. Note that, we compare the performance of DSAs using several metrics, see S1 Appendix H.4.1. To assess the likelihood of receiving a transplant in a particular DSA, we divide the number of transplants by the number of patients waiting at that DSA. Alternatively, one can consider median time-to-transplant while comparing DSAs, which gives similar results. Moreover, we eliminate such patients who get multi-listed at the second transplant center at least one year after the snapshot date which corresponds to 0.68% of the input data. Here, we exclude such patients to make sure the patient is listed in both DSAs for a substantial amount of time, otherwise, some inaccuracies may occur.

H.4.1 Donation Service Area (DSA) Evaluation Metrics

In this section, we compare the performance of DSAs using several key metrics. This analysis provides a comprehensive evaluation of how different DSAs perform, highlighting the disparities and outcomes across regions.

We compare the performance of DSAs using the following metrics:

1. Median time-to-transplant
2. Mean time-to-transplant
3. Transplant Rate: We divide the number of transplants to the number of patients waiting at that DSA. It shows the fraction of the patients who received a transplant.

Table S67 shows the number of transplants, evaluation metrics and rankings with respect to each metric. We observe that the rankings for mean and median time-to-transplant are consistent with each other. However, rankings for transplant rate can be different for some DSAs.

Table S67. DSA Evaluation Metrics.

DSA	Number of Transplants	Median Time-to-Tx	Mean Time-to-Tx	Tx Rate	Rank Median	Rank Mean	Rank Tx Rate
CADN	10,534	1,228	1,349	0.24	58	58	58
ALOB	3,579	979	1,173	0.3	57	57	56
CORS	2,988	914	964	0.4	56	51	42
TXSA	4,122	864	1073	0.25	55	56	57
NMOP	1,241	854	947	0.46	54	50	29
NYRT	10,293	854	1,051	0.32	54	55	54
NJTO	2,657	841	966	0.32	52	52	55
CASD	2,881	833	995	0.35	51	53	52
CAOP	12,860	785	1048	0.36	50	54	50
OHLB	3,355	776	880	0.44	49	47	31
CTOP	1,347	764	864	0.47	48	44	27
MAOB	6,724	755	883	0.39	47	48	43
MIOP	6,863	750	870	0.42	46	46	37
ILIP	8,514	743	926	0.38	45	49	48
NYFL	1,549	721	867	0.43	44	45	33
WALC	5,105	713	768	0.54	43	34	16
PADV	10,595	691	859	0.38	42	43	49
NCNC	6,085	672	858	0.43	41	42	34
HIOP	986	660	793	0.51	40	37	20
VATB	4,881	660	814	0.4	40	39	40
WIDN	1,837	648	768	0.36	38	34	51
DCTC	7,178	646	829	0.4	37	40	41
MOMA	3,918	643	707	0.54	36	30	17
GALL	6,325	642	839	0.35	35	41	53
PRLL	1,383	624	731	0.44	34	32	32
MNOP	4,788	621	810	0.39	33	38	44
LAOP	4,546	585	788	0.39	32	36	45
CAGS	3,417	549	716	0.43	31	31	35
NCCM	1,752	548	673	0.47	30	26	26
NYAP	1,302	527	641	0.55	29	22	14
TXGC	7,245	503	683	0.38	28	29	47
TNDS	2,757	501	736	0.41	27	33	38
AZOB	5,670	495	671	0.52	26	25	19
MDPC	2,428	494	657	0.38	25	24	46
INOP	3,592	480	673	0.48	24	26	24
PATF	5,362	467	630	0.48	23	20	25
OKOP	2,697	466	573	0.56	22	17	13
TNMS	3,703	453	679	0.47	21	28	28
FLMP	6,251	449	638	0.5	20	21	23
MWOB	4,385	439	512	0.63	19	12	6
FLUF	4,330	435	655	0.43	18	23	36

TXSB	7,692	428	617	0.41	17	19	39
SCOP	3,358	422	581	0.51	16	18	21
OHLP	2,710	419	550	0.56	15	14	11
FLWC	4,406	382	495	0.59	14	9	7
WIUW	3,409	380	556	0.56	13	15	10
FLFH	2,888	378	489	0.65	12	7	3
NYWN	1,642	376	570	0.55	11	16	15
UTOP	2,081	371	491	0.63	10	8	5
ORUO	2,523	369	503	0.65	9	10	2
MSOP	1,517	361	533	0.45	8	13	30
NVLV	1,183	354	448	0.56	7	4	9
IAOP	1,802	327	505	0.57	6	11	8
AROR	1,774	320	431	0.64	5	3	4
KYDA	2,666	304	485	0.53	4	6	18
NEOR	2,262	280	390	0.56	3	2	12
OHOV	1,534	254	479	0.51	2	5	22
OHLC	1,688	230	355	0.67	1	1	1

I Implementation Details of Survival Analysis in Section 3.3.1

We estimate how long a patient will survive without a xenotransplant. Some patients in the data are still alive at the end of the observation period or transplant centers are not able to contact some patients, resulting in censored observations. Survival analysis allows us to incorporate information from both censored and uncensored observations in estimating model parameters. In survival analysis, the response variable is composed of two parts: (i) the time elapsed between the snapshot date and the death date (last day of observation, if the patient is still alive) and (ii) survival status which records if the event of interest, death, occurred or not. Then, we fit a survival model and generate individual survival curves using the fitted model.

We first train the model on the training data and measure its performance on the test set to evaluate whether it yields an accurate outcome on data that the model has never seen before. We select 80% (train), 20% (test) for our train-test split to allow for enough data to robustly evaluate developed models. Similarly, for each cohort, we construct the test data from a particular group and train the model using the rest of the patients in that cohort. We apply stratified sampling while creating the test set to ensure that the test set is representative of the entire data set. We tune hyperparameters using repeated k-fold cross-validation (5-fold, 3 times).

Machine learning algorithms have different requirements for the input data set. Cox and RSF do not have any restrictions. However, the input of DeepSurv needs to be numeric so we apply one-hot encoding to all categorical variables. That is, we create numeric indicators of each factor in the categorical features. Moreover, we standardize each numeric feature to have a mean of zero and a standard deviation of one for all models. It allows for the comparison of multiple features in different units.

J Aggressiveness Analysis of Transplant Centers in Defining Cohorts

Recall that to define the highest-need patient groups, we take the union of the three most important demographic variables identified by the RSF and Cox models, see S1 Appendix A. Moreover, we incorporate the aggressiveness of transplant centers, even

though it is not among the top three variables. This is because a patient's likelihood of receiving a transplant is affected by the transplant center where they are registered. In this section, we define a measure of transplant center aggressiveness based on the likelihood of a patient receiving a transplant.

Transplant centers are evaluated based on their patient outcomes, such as patient and graft survival rates, waitlist mortality, and rates of complications. These outcomes are monitored and reported. Also, they need to comply with national standards for patient safety and quality of care. Therefore, they have incentives to minimize risks associated with transplant operations to provide the best possible outcomes for their patients.

As a result, transplant centers can be selective at different stages of the process. Fig S8 depicts the steps to kidney transplantation and decision makers at each stage. First, transplant centers may not prefer to register patients with adverse medical conditions. As the next stage, registration is followed by the opt-in/out decisions for different organ types which is a joint decision of patients and transplant centers. Medical professionals provide guidance and recommendations. As such, they influence patients' decisions. As ultimate decision makers, patients may not opt-in for less desirable organs since the survival probabilities are lower with such organs. Note that opt-in status affects the number of organ offers a patient sees. Lastly, as a joint decision of transplant center and the patient, a marginal organ may not be accepted. This can be observed more frequently for certain subpopulations such as patients with age 65+.

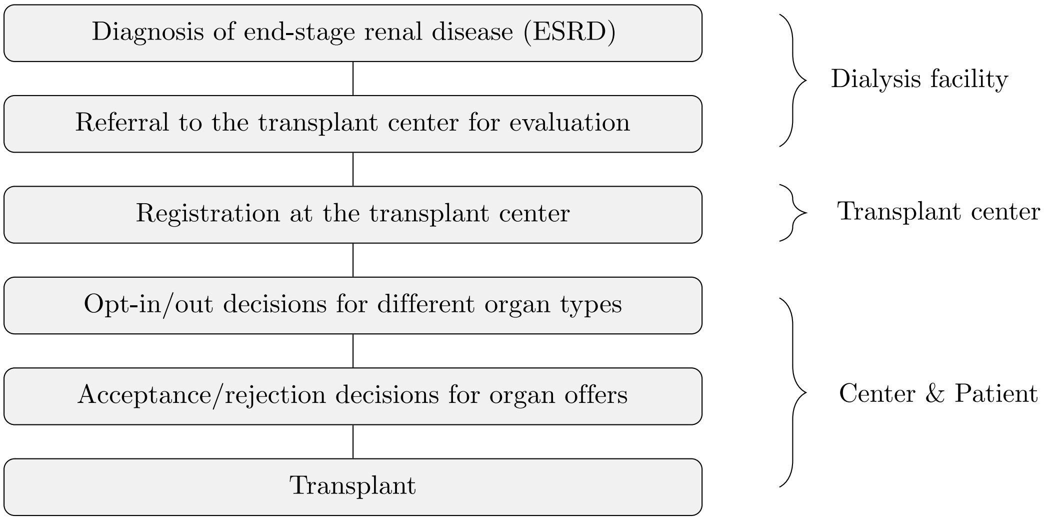

Fig S8. Steps to kidney transplantation and decision makers at each stage.

In our analysis, we define a notion of aggressiveness to assess the risk profile of transplant centers. To that end, we calculate the following rates:

- **Acceptance rate:** It is the ratio of marginal organs accepted by the transplant center to the total number of such organs seen by the patients registered at that center.

- **Offer seen rate:** For each patient, we calculate the fraction of marginal organs (offered in the entire US throughout the entire time horizon) he could potentially see based on his initial opt-in status. Then, we take the average across all patients registered at the transplant center, see Section J.2.

In other words, we define the aggressiveness criteria as the acceptance rate of marginal organs, corrected by the offer seen rate. To be more specific, we multiply the acceptance rate by the offer seen rate for each transplant center and use this as the measure of aggressiveness. Lastly, we address how this measure varies across different age groups.

The rest of this section is structured as follows. Section J.1 provides the definition of a marginal organ. Section J.2 includes the opt-in percentages of patients upon registration for marginal organs and introduces the offer seen rate. Lastly, Section J.3 provides the (corrected) acceptance rate for marginal organs for different age groups.

Note that we calculate the aggressiveness measure for all transplant centers. In this write-up, we only present the transplant centers that might participate in xenotransplant clinical trials. Therefore, we provide sample results for the centers listed in Table S68.

Table S68. List of transplant centers that might participate in xenotransplant clinical trials.

ID	City	Name
7	Birmingham	University of Alabama Hospital
119	Miami	Jackson Memorial Hospital
190	Chicago	University of Chicago Medical Center
298	Boston	Massachusetts General Hospital
313	Baltimore	University of Maryland Medical System
350	Minneapolis	University of Minnesota Medical Center
382	Durham	Duke University Hospital
456	New York	NYU Langone Health

J.1 Definition of Marginal Organs

There are observable characteristics that make organs less desirable. Those features are listed as follows: donor's cancer history, smoking status, substance use, blood or urine infection, protein in the urine, and whether or not a biopsy is done for the donor. Table S69 shows the number and the fraction of donors with less desirable donor characteristics, and Table S70 shows the frequency of such characteristics. In our analysis, we consider an organ as "scarred" if it has three or more of the features that make organs less desirable. As seen from Table S70, 29% of the organs are scarred.

Table S69. The number and fraction of donors with less desirable characteristics.

Feature	# donor	% donor
Cancer history	1,569	3.4
Smoking status	10,994	23.8
Substance use	11,362	24.6
Blood or urine infection	12,014	26.0
Protein in urine	23,527	51.0
Biopsy	28,523	61.8

In addition, there are other characteristics that make an organ less desirable. Transplant centers and patients can jointly decide on whether or not to opt in to receive offers of such organs. In particular, opt-in decisions are made if the donor satisfies any of the following conditions:

- Donor has KDPI score greater than or equal to 85 (KDPI$\geq$ 85).
- Donor is Hepatitis B Core (HBC) positive.
- Donor is Hepatitis C Virus (HCV) positive.
- The organ is donated after cardiac death (DCD).

Table S70. Frequency of less desirable donor characteristics. An organ is considered as scarred if it has three or more less desirable features. Therefore, 29% (=100–71) of the organs are scarred.

# less desirable features	# donors	% donors	% cumulative
0	4,668	10.1	10.1
1	12,683	27.5	37.6
2	15,397	33.4	**71.0**
3	9,650	20.9	91.9
4	3,247	7.0	98.9
5	486	1.1	99.9
6	24	0.1	100.0

Table S71 shows the number and fraction of donors with HBC, HCV, DCD, KDPI$\geq$ 85 or scarred organs. It also includes the fraction of such organs transplanted. In our analysis, we assume that an organ is "marginal" if it is HBC, HCV, DCD, KDPI$\geq$ 85 or scarred. As seen from Table S71, marginal organs corresponds to 59.5% of the organs.

Table S71. The number and fraction of donors with HBC, HCV, DCD, KDPI $\geq$ 85 or scarred organs and the fraction of such organs transplanted.

Feature	# donor	% donor	% transplant
HBC positive	2,586	5.6	56.5
HCV positive	4,785	10.4	61.2
DCD	10,691	23.2	75.9
KDPI $\geq$ 85	9,089	19.7	37.2
Scarred	13,407	29.0	65.0
**HBC, HCV, DCD, KDPI $\geq$ 85 or Scarred**	**27,446**	**59.5**	**65.2**

J.2 Opt-in Status of Patients and Offer Seen Rate

Patients have different preferences towards marginal organs. Table S72 shows the percentage of patients who opted-in for HBC, HCV, DCD, and KDPI $\geq$ 85 donor organs upon registration. Note that, if a patient has registered before 2014 and is still on the list as of 2016, we consider the following for her opt-in KDPI > 85 status: max(**1**opt-in for ECD, **1**opt-in for KDPI $\geq$ 85). It is important to mention that patients can not opt in or opt out for scarred organs.

Table S72. The percentage of patients who opted-in for HBC, HCV, DCD and KDPI $\geq$ 85.

ID	City	Name	HBC	HCV	DCD	KDPI $\geq$ 85
7	Birmingham	University of Alabama Hospital	30.6	7.5	15.6	25.6
119	Miami	Jackson Memorial Hospital	64.9	7.2	7.6	38.0
190	Chicago	UChicago Medical Center	44.2	5.5	24.7	38.8
298	Boston	Massachusetts General Hospital	99.1	61.1	39.1	31.2
313	Baltimore	Uni. of Maryland Medical System	81.5	14.7	18.2	75.7
350	Minneapolis	Uni. of Minnesota Medical Center	22.9	0.5	25.3	21.9
382	Durham	Duke University Hospital	56.3	60.4	19.9	31.6
456	New York	NYU Langone Health	99.9	19.8	22.2	78.7

We observe that opt-in rates for KDPI $\geq$ 85 donors are considerably high at NYU and Maryland compared to other centers. On the other hand, opt-in rates for KDPI $\geq$ 85 donors are considerably low at Alabama and Minnesota compared to others.

Moreover, NYU and Mass General have the highest fraction of patients who opted in for HBC.

Notice that opt-in status affects the number of organ offers a patient sees. For each patient, we calculate the fraction of marginal organs (HBC, HCV, DCD or KDPI ≥ 85) he can see based on his initial opt-in status. Here, we consider all organs offered in the entire US throughout the entire time horizon. In other words, we address the question of what fraction of the organs would they actually see if all such organs were available to them. This yields the “joint opt-in distribution” of patients which we refer to as the “offer seen rate”. Since patients cannot opt in/out for scarred organs, scarred organs do not affect the offer seen rate. Next, we take the average of offer seen rates of all patients registered at each transplant center. Table S73 shows the average offer seen rates at each transplant center.

Table S73. Offer seen rate, i.e. joint opt-in distribution, for transplant centers.

ID	City	Name	Offer Seen Rate [%]
7	Birmingham	University of Alabama Hospital	15.95
119	Miami	Jackson Memorial Hospital	17.64
190	Chicago	UChicago Medical Center	23.57
298	Boston	Massachusetts General Hospital	39.32
313	Baltimore	Uni. of Maryland Medical System	35.67
350	Minneapolis	Uni. of Minnesota Medical Center	16.82
382	Durham	Duke University Hospital	28.33
456	New York	NYU Langone Health	40.07

Note that a higher offer seen rate indicates a higher joint opt-in rate. Table S73 shows that NYU has the highest offer seen rate. That is, patients registered at NYU are more willing to receive less desirable organs compared to other centers.

J.3 Acceptance Rate

The acceptance rate corresponds to the ratio of marginal organs accepted by the transplant center to the total number of such organs seen by the patients registered at that center. It is important to note that we consider the acceptance rate (or rejection rate) at the transplant center level. For example, if a transplant center sees a donor and does not accept her organ(s) for its n patients, then we do not count it as n rejection, instead, we consider it as a single rejection.

The acceptance rate of marginal organs alone might not be sufficient to assess the risk profile of centers. This is because, the transplant centers might have been selective and patients registered at those centers might have already opt-out of low-quality organs, decreasing the number of organs seen by the center. To account for this potential selection bias, we multiply the acceptance rate of marginal organs by the offer seen rate introduced in Section J.2. That is, in our analysis, we consider the offer seen rate as the correction factor.

For marginal organs, Table S74 shows the number of organs accepted by that transplant center, the total number of organs seen by the center, the offer seen rate, and (un)corrected acceptance rate.

Lastly, we address how this measure varies across different age groups. Table S75 shows the offer seen rates, i.e. joint opt-in percentages, across three age groups. Moreover, Table S76 shows rankings among all 202 transplant centers with respect to the corrected acceptance rate across three age groups. Note that a lower ranking indicates a more aggressive transplant center.

Table S74. Acceptance rate of marginal organs. *# accept* and *# organ* show the number of organs accepted by that transplant center and the total number of organs seen by the center, respectively. *Acceptance rate* is the ratio of *# accept* to *# organ*. *Corrected acceptance rate* is the multiplication of the acceptance rate with the offer seen rate.

ID	City	Name	# accept	# organ	Acpt rate [%]	Offer seen rate	Corr. rate [%]
7	Birmingham	University of Alabama Hospital	298	6265	4.8	0.16	0.77
119	Miami	Jackson Memorial Hospital	919	13021	7.1	0.18	1.25
190	Chicago	UChicago Medical Center	151	6181	2.4	0.24	0.57
298	Boston	Massachusetts General Hospital	227	9432	2.4	0.39	0.94
313	Baltimore	Uni. of Maryland Medical System	391	18657	2.1	0.36	0.75
350	Minneapolis	Uni. of Minnesota Medical Center	179	8146	2.2	0.17	0.37
382	Durham	Duke University Hospital	341	10245	3.3	0.28	0.93
456	New York	NYU Langone Health	384	11931	3.2	0.40	1.28

Table S75. Offer seen rate, i.e. joint opt-in distribution, across age groups.

ID	City	Name	All patients	Age <50	Age [50,64)	Age 65+
7	Birmingham	University of Alabama Hospital	16.0	9.5	20.4	31.8
119	Miami	Jackson Memorial Hospital	17.6	11.4	20.0	24.5
190	Chicago	UChicago Medical Center	23.6	17.1	26.1	37.0
298	Boston	Massachusetts General Hospital	39.3	25.7	41.4	53.4
313	Baltimore	Uni. of Maryland Medical System	35.7	33.1	37.6	36.2
350	Minneapolis	Uni. of Minnesota Medical Center	16.8	11.9	18.5	25.8
382	Durham	Duke University Hospital	28.3	19.0	35.4	35.6
456	New York	NYU Langone Health	40.1	37.2	42.3	40.9

Table S76. Ranking with respect to the corrected acceptance rate across age groups. The rank columns indicate each transplant center's position among all 202 centers, with lower ranks reflecting more aggressive centers. The final column reports the simple average of the three preceding rank columns.

ID	City	Name	Rank (All)	Rank Age <50	Rank Age [50,64)	Rank Age 65+	Avg.
7	Birmingham	University of Alabama Hospital	92	82	65	94	80.3
119	Miami	Jackson Memorial Hospital	40	67	37	14	39.3
190	Chicago	UChicago Medical Center	121	116	111	98	108.3
298	Boston	Massachusetts General Hospital	62	62	73	62	65.7
313	Baltimore	Uni. of Maryland Medical System	96	118	86	114	106.0
350	Minneapolis	Uni. of Minnesota Medical Center	147	148	151	127	142.0
382	Durham	Duke University Hospital	63	47	46	103	65.3
456	New York	NYU Langone Health	39	46	35	53	44.7

K Confusion Matrices and Evaluation Metrics for Cohort Analysis

K.1 Formal Definitions of Hit Rate and Capture Rate

Hit rate and capture rate can be further explained using a confusion matrix, see Table S77. For a particular τ value, the patients who are correctly identified by the model are denoted as True Positive (TP) whereas the incorrectly labeled patients are represented as False Positive (FP). Hit rate corresponds to precision which can be expressed as TP/(TP + FP). On the other hand, capture rate corresponds to sensitivity (recall) which is calculated as TP/(TP + FN).

In addition to hit rate and capture rate, we define another metric called 'labeling rate' as the fraction of the patients identified by the model. To be more specific, it is calculated as (TP + FP)/(TP + FP + FN + TN). This metric will serve as a natural benchmark for potential incentive mechanisms.

Table S77. Confusion matrix format.

		True Class	
		**Positive (Dead)**	**Negative (Alive)**
**Hypothesis Output**	**Positive (Dead)**	True Positive (TP)	False Positive (FP)
	**Negative (Alive)**	False Negative (FN)	True Negative (TN)

K.2 Summary Tables of Survival Analysis

Table S78 shows the confusion matrices for all patients whereas Table S79 provides the performance metrics for different expected xenograft survivals, denoted by n, see Table S77 for an explanation of different entries for each case displayed in Table S78.

Table S78. Confusion matrices from survival analysis across n.

n	RSF		Deepsurv		Cox	
0.5	50	19	378	203	193	102
	11969	17996	11146	18307	10800	18939
1.0	209	69	769	355	420	208
	12678	17078	11719	17191	11538	17868
1.5	599	191	1414	580	901	410
	12944	16300	11897	16143	11892	16831
2.0	1482	474	2303	940	1607	645
	12716	15362	11682	15109	11887	15895
3.0	4208	1453	5040	2108	3548	1311
	10833	13540	10004	12882	11035	14140
4.0	7242	2884	8127	3579	5958	2275
	8329	11579	7468	10860	9326	12475
5.0	10023	4362	10803	5029	8266	3392
	6004	9645	5256	8946	7470	10906

Table S80 provides a summary of the results for all patients whereas Table S81 summarizes the results for each (cohort, n) pair. The results are robust to cohorting patients in terms of the hit rate but the cohort analysis improves the capture rate, see Table S81. For example, if we compare the results for all patients with those for Cohort 3 for 2-year graft survival in RSF, the hit rates are comparable (75% vs 73%). However, there is a significant difference in terms of the capture rate. The capture rate is 22.1% for Cohort 3 for 2-year graft survival in RSF whereas, it is 10.4% for 2-year graft survival in RSF when we consider all patients.

Table S79. Evaluation metrics across n.

Method	n	Hit Rate	Capture Rate
RSF	0.5	72.5	0.4
	1.0	75.2	1.6
	1.5	75.8	4.4
	2.0	75.8	10.4
	3.0	74.3	28.0
	4.0	71.5	46.5
	5.0	69.7	62.5
Deepsurv	0.5	65.1	3.3
	1.0	68.4	6.2
	1.5	70.9	10.6
	2.0	71.0	16.5
	3.0	70.5	33.5
	4.0	70.4	52.1
	5.0	70.2	67.3
Cox	0.5	65.4	1.8
	1.0	66.9	3.5
	1.5	68.7	7.0
	2.0	71.4	11.9
	3.0	73.0	34.3
	4.0	72.4	39.0
	5.0	70.9	52.5

Table S80. Summary of the results of each objective for all patients. It shows the method, hit rate, and capture rate for all three objectives across $n \in \{0.5, 1, 1.5, 2, 3, 4, 5\}$.

Cohort	Yr	Highest Hit Rate			Highest Capture Rate		
		Method	% Hit	% Captured	Method	% Hit	% Captured
All	0.5	RSF	72.5	0.4	Deep	65.1	3.3
	1.0	RSF	75.2	1.6	Deep	68.4	6.2
	1.5	RSF	75.8	4.4	Deep	70.9	10.6
	2.0	RSF	75.8	10.4	Deep	71.0	16.5
	3.0	RSF	74.3	28.0	Deep	70.5	33.5
	4.0	Cox	72.4	39.0	Deep	70.4	52.1
	5.0	Cox	70.9	52.5	Deep	70.2	67.3

Table S81. Summary of the results for each objective for each (cohort, n) pair. Wherever we mark with *, it corresponds to the case in which the maximum number of correctly identified patients is less than 15. As such, we will not consider them due to the very small number of patients.

Cohort	Yr	Highest Hit Rate			Highest Capture Rate		
		Method	% Hit	% Captured	Method	% Hit	% Captured
C1	0.5	RSF	77.8	1.1	Cox	69.1	2.0
	1.0	RSF	77.3	1.5	Cox	71.9	3.2
	1.5	RSF	74.4	2.3	Cox	70.9	4.8
	2.0	RSF	73.5	5.1	Deep	69.8	9.3
	3.0	RSF	71.2	15.9	Deep	68.3	27.6
	4.0	RSF	71.6	40.6	Deep	69.7	53.8
	5.0	RSF	70.2	66.4	Deep	70.0	74.1
C2	0.5	Deep	100.0	0.1*	Cox	92.3	1.7
	1.0	Deep	100.0	1.2*	Cox	87.5	2.3
	1.5	RSF	100.0	0.9*	Cox	75.4	4.1
	2.0	RSF	83.3	3.5	Cox	77.9	9.1
	3.0	RSF	74.7	23.8	Deep	70.5	35.0
	4.0	RSF	75.9	58.4	Deep	74.9	69.7
	5.0	RSF	77.3	81.9	Deep	76.8	90.3
C3	0.5	Cox	70.9	11.0	Cox	70.9	11.0
	1.0	Cox	71.1	12.9	Cox	71.1	12.9
	1.5	RSF	72.3	13.8	Deep	70.8	21.5
	2.0	RSF	73.0	22.1	Deep	71.1	32.9
	3.0	RSF	73.1	42.2	Deep	70.9	57.0
	4.0	RSF	72.9	65.4	Deep	69.8	79.5
	5.0	RSF	70.8	82.0	Deep	69.6	91.6
C4	0.5	Cox	33.3*	0.3	Cox	33.3*	0.3
	1.0	RSF	75.0*	0.6	Cox	66.7*	0.9
	1.5	RSF	84.4	4.7	Deep	83.9	4.5
	2.0	RSF	76.0	12.6	Deep	75.8	15.4
	3.0	RSF	75.2	54.3	Deep	74.8	65.1
	4.0	RSF	77.7	86.9	Deep	76.8	92.3
	5.0	Cox	79.1	97.7	Deep	79.1	99.0
C5	0.5	Deep	100.0*	0.2	Deep	100.0*	0.2
	1.0	Deep	100.0*	0.4	Deep	100.0*	0.4
	1.5	Cox	66.7	0.3	Deep	62.5	0.8
	2.0	Deep	67.2	2.9	Deep	67.2	2.9
	3.0	RSF	74.3	4.6	Deep	69.7	12.8
	4.0	RSF	71.7	25.2	Deep	69.3	33.6
	5.0	RSF	71.2	56.6	Deep	70.0	60.4

Cohort	Yr	Highest Hit Rate			Highest Capture Rate		
		Method	% Hit	% Captured	Method	% Hit	% Captured
C6	0.5	-	-	-	-	-	-
	1.0	-	-	-	-	-	-
	1.5	-	-	-	-	-	-
	2.0	Deep	100.0*	0.2	Cox	66.7*	0.9
	3.0	Cox	69.7	12.4	Cox	69.7	12.4
	4.0	Cox	74.9	47.0	Deep	72.8	51.1
	5.0	RSF	75.7	69.4	Deep	74.6	81.8
C7	0.5	Cox	66.7	3.6	Cox	66.7	3.6
	1.0	Cox	65.4	4.4	Cox	65.4	4.4
	1.5	RSF	65.0	2.8	Cox	57.5	5.2
	2.0	RSF	73.0	5.4	Cox	67.1	11.6
	3.0	Cox	70.4	24.4	Cox	70.4	24.4
	4.0	Cox	68.8	42.8	Deep	59.6	49.4
	5.0	Cox	68.6	64.8	Deep	62.7	84.4
C8	0.5	RSF	50.0*	0.6	Cox	40.0	1.2
	1.0	RSF	66.7*	0.9	Cox	50.0	1.4
	1.5	RSF	66.7	1.6	Cox	47.6	4.0
	2.0	Deep	78.6	3.7	Cox	65.6	14.2
	3.0	RSF	72.2	34.3	Cox	70.9	41.5
	4.0	Cox	74.9	70.8	Deep	71.4	87.7
	5.0	Cox	79.1	90.0	Deep	78.1	100.0
C9	0.5	Cox	72.2	1.5	Cox	72.2	1.5
	1.0	Deep	76.9	2.0	Cox	74.2	2.3
	1.5	Deep	74.6	4.3	Deep	74.6	4.3
	2.0	Cox	75.2	8.4	Deep	72.1	9.8
	3.0	Deep	71.2	32.0	Deep	71.2	32.0
	4.0	RSF	70.1	47.5	Deep	69.7	57.8
	5.0	Deep	69.3	76.3	Deep	69.3	76.3
C10	0.5	-	-	-	-	-	-
	1.0	-	-	-	-	-	-
	1.5	Deep	81.2	3.5	Cox	57.5	6.2
	2.0	RSF	75.0	2.2	Cox	68.2	13.4
	3.0	RSF	76.4	26.4	Cox	72.7	34.3
	4.0	RSF	75.5	58.0	Deep	74.3	63.4
	5.0	Cox	78.1	83.7	Deep	76.9	87.5
C11	0.5	Cox	100.0*	0.9	Cox	100.0*	0.9
	1.0	Cox	50.0*	0.7	Cox	50.0*	0.7
	1.5	Cox	33.3*	2.3	Cox	33.3*	2.3
	2.0	Deep	100.0*	0.4	Cox	44.4*	5.5
	3.0	Deep	62.1	6.1	Cox	60.0	17.3
	4.0	Deep	73.7	18.7	Cox	70.4	29.9
	5.0	Deep	80.3	38.8	Cox	77.8	44.1

K.3 Results of Survival Analysis

We compare the life expectancy of patients with and without a xeno-kidney. In the survival analysis, we generate individual survival curves and calculate the area under these curves to estimate patient life expectancies. This approach allow us to directly estimate survival benefits under the status quo (without xenotransplantation) and compare them with survival benefits when xenotransplantation is available, see Section 3.3.1.

Table S82 presents the formulas for the evaluation metrics. For each cohort, the corresponding confusion matrices and performance metrics for survival analysis are provided in Tables S83 - S106. In those tables, whenever there are too few observations (less than 15), i.e., when the estimates are too noisy, we left the corresponding entries of the table empty. This happens typically when n is low.

Table S82. Formulas of the evaluation metrics.

Performance Measure	Formula
Accuracy	$\frac{\text{TP + TN}}{\text{TP+FP+TN+FN}}$
Error rate	$1 - \text{Accuracy}$
Precision	$\frac{\text{TP}}{\text{TP + FP}}$
Recall	$\frac{\text{TP}}{\text{TP + FN}}$
Sensitivity (= Recall)	$\frac{\text{TP}}{\text{TP + FN}}$
Specificity	$\frac{\text{TN}}{\text{TN + FP}}$
F-measure	$\frac{(1+\beta)^2 \cdot \text{Recall} \cdot \text{Precision}}{\beta^2 \cdot \text{Recall} + \text{Precision}}$
Kappa	$\frac{2(TP \cdot TN - FN \cdot FP)}{(TP+FP)(FP+TN)+(TP+FN)(FN+TN)}$

K.4 Results of Classification Analysis

As a robustness check for survival analysis discussed in Section 3.3.1, we also perform a classification analysis using three classification models, namely Gradient Boosting Machine (GBM), Random Forest (RF) and LogisticBoost (LB). In this setting, we construct a binary variable indicating whether a patient died within n years after the snapshot date. However, those methods are uninformative, yielding a hit rate around 50% (no different from flipping a coin) as mentioned in Section 4. For each cohort, the corresponding confusion matrices and performance metrics for survival analysis are provided in Tables S107 - S130.

Table S83. Confusion matrices across n (Cohort 0) (Survival).

n	RSF		Deepsurv		Cox	
0.5	50	19	378	203	193	102
	11969	17996	11146	18307	10800	18939
1.0	209	69	769	355	420	208
	12678	17078	11719	17191	11538	17868
1.5	599	191	1414	580	901	410
	12944	16300	11897	16143	11892	16831
2.0	1482	474	2303	940	1607	645
	12716	15362	11682	15109	11887	15895
3.0	4208	1453	5040	2108	3548	1311
	10833	13540	10004	12882	11035	14140
4.0	7242	2884	8127	3579	5958	2275
	8329	11579	7468	10860	9326	12475
5.0	10023	4362	10803	5029	8266	3392
	6004	9645	5256	8946	7470	10906

Table S84. Evaluation metrics across n (Cohort 0) (Survival).

Method	n	Accuracy	Error Rate	Precision	Recall	Specificity	F1	Kappa
RSF	0.5	0.601	0.399	0.725	0.004	0.999	0.008	0.004
	1.0	0.576	0.424	0.752	0.016	0.996	0.032	0.014
	1.5	0.563	0.437	0.758	0.044	0.988	0.084	0.036
	2.0	0.561	0.439	0.758	0.104	0.970	0.183	0.078
	3.0	0.591	0.409	0.743	0.280	0.903	0.407	0.183
	4.0	0.627	0.373	0.715	0.465	0.801	0.564	0.262
	5.0	0.655	0.345	0.697	0.625	0.689	0.659	0.312
Deepsurv	0.5	0.622	0.378	0.651	0.033	0.989	0.062	0.027
	1.0	0.598	0.402	0.684	0.062	0.980	0.113	0.048
	1.5	0.585	0.415	0.709	0.106	0.965	0.185	0.078
	2.0	0.580	0.420	0.710	0.165	0.941	0.267	0.112
	3.0	0.597	0.403	0.705	0.335	0.859	0.454	0.194
	4.0	0.632	0.368	0.694	0.521	0.752	0.595	0.271
	5.0	0.658	0.342	0.682	0.673	0.640	0.677	0.313
Cox	0.5	0.637	0.363	0.654	0.018	0.995	0.034	0.015
	1.0	0.609	0.391	0.669	0.035	0.988	0.067	0.028
	1.5	0.590	0.410	0.687	0.070	0.976	0.128	0.053
	2.0	0.583	0.417	0.714	0.119	0.961	0.204	0.087
	3.0	0.589	0.411	0.730	0.243	0.915	0.365	0.161
	4.0	0.614	0.386	0.724	0.390	0.846	0.507	0.234
	5.0	0.638	0.362	0.709	0.525	0.763	0.603	0.284

Table S85. Confusion matrices across n (Cohort 1) (Survival).

n	RSF		Deepsurv		Cox	
0.5	21	6	30	13	38	17
	1926	3423	1867	3466	1865	3456
1.0	34	10	57	22	69	27
	2190	3142	2121	3176	2070	3210
1.5	58	20	115	48	117	48
	2418	2880	2356	2857	2301	2910
2.0	139	50	254	110	211	91
	2600	2587	2481	2531	2462	2612
3.0	493	199	859	398	640	280
	2614	2070	2254	1865	2410	2046
4.0	1366	541	1810	787	1416	613
	1995	1474	1556	1223	1910	1437
5.0	2303	978	2580	1108	2270	1030
	1167	928	902	786	1183	893

Table S86. Evaluation metrics across n (Cohort 1) (Survival).

Method	n	Accuracy	Error Rate	Precision	Recall	Specificity	F1	Kappa
RSF	0.5	0.641	0.359	0.778	0.011	0.998	0.021	0.011
	1.0	0.591	0.409	0.773	0.015	0.997	0.030	0.014
	1.5	0.547	0.453	0.744	0.023	0.993	0.045	0.018
	2.0	0.507	0.493	0.735	0.051	0.981	0.095	0.031
	3.0	0.477	0.523	0.712	0.159	0.912	0.260	0.062
	4.0	0.528	0.472	0.716	0.406	0.732	0.519	0.121
	5.0	0.601	0.399	0.702	0.664	0.487	0.682	0.147
Deepsurv	0.5	0.650	0.350	0.698	0.016	0.996	0.031	0.016
	1.0	0.601	0.399	0.722	0.026	0.993	0.051	0.023
	1.5	0.553	0.447	0.706	0.047	0.983	0.087	0.032
	2.0	0.518	0.482	0.698	0.093	0.958	0.164	0.050
	3.0	0.507	0.493	0.683	0.276	0.824	0.393	0.090
	4.0	0.564	0.436	0.697	0.538	0.608	0.607	0.136
	5.0	0.626	0.374	0.700	0.741	0.415	0.720	0.160
Cox	0.5	0.650	0.350	0.691	0.020	0.995	0.039	0.019
	1.0	0.610	0.390	0.719	0.032	0.992	0.062	0.029
	1.5	0.563	0.437	0.709	0.048	0.984	0.091	0.035
	2.0	0.525	0.475	0.699	0.079	0.966	0.142	0.045
	3.0	0.500	0.500	0.696	0.210	0.880	0.322	0.081
	4.0	0.531	0.469	0.698	0.426	0.701	0.529	0.113
	5.0	0.588	0.412	0.688	0.657	0.464	0.672	0.120

Table S87. Confusion matrices across n (Cohort 2) (Survival).

n	RSF		Deepsurv		Cox	
0.5	0	0	1	0	12	1
	681	1788	693	1775	690	1766
1.0	1	0	11	0	21	3
	889	1579	894	1564	884	1561
1.5	10	0	35	8	46	15
	1087	1372	1072	1354	1065	1343
2.0	45	9	113	41	116	33
	1230	1185	1167	1148	1154	1166
3.0	363	123	539	226	514	194
	1164	819	1002	702	1019	742
4.0	999	318	1209	405	1139	386
	712	440	526	329	568	376
5.0	1510	443	1668	503	1607	476
	333	183	180	118	229	157

Table S88. Evaluation metrics across n (Cohort 2) (Survival).

Method	n	Accuracy	Error Rate	Precision	Recall	Specificity	F1	Kappa
RSF	0.5	0.724	0.276		0.000	1.000		0.000
	1.0	0.640	0.360	1.000	0.001	1.000	0.002	0.001
	1.5	0.560	0.440	1.000	0.009	1.000	0.018	0.010
	2.0	0.498	0.502	0.833	0.035	0.992	0.068	0.027
	3.0	0.479	0.521	0.747	0.238	0.869	0.361	0.088
	4.0	0.583	0.417	0.759	0.584	0.580	0.660	0.144
	5.0	0.686	0.314	0.773	0.819	0.292	0.796	0.119
Deepsurv	0.5	0.719	0.281	1.000	0.001	1.000	0.003	0.002
	1.0	0.638	0.362	1.000	0.012	1.000	0.024	0.015
	1.5	0.563	0.437	0.814	0.032	0.994	0.061	0.028
	2.0	0.511	0.489	0.734	0.088	0.966	0.158	0.052
	3.0	0.503	0.497	0.705	0.350	0.756	0.467	0.091
	4.0	0.623	0.377	0.749	0.697	0.448	0.722	0.138
	5.0	0.723	0.277	0.768	0.903	0.190	0.830	0.112
Cox	0.5	0.720	0.280	0.923	0.017	0.999	0.034	0.023
	1.0	0.641	0.359	0.875	0.023	0.998	0.045	0.027
	1.5	0.563	0.437	0.754	0.041	0.989	0.078	0.033
	2.0	0.519	0.481	0.779	0.091	0.972	0.163	0.062
	3.0	0.509	0.491	0.726	0.335	0.793	0.459	0.109
	4.0	0.614	0.386	0.747	0.667	0.493	0.705	0.151
	5.0	0.714	0.286	0.771	0.875	0.248	0.820	0.141

Table S89. Confusion matrices across n (Cohort 3) (Survival).

n	RSF		Deepsurv		Cox	
0.5	70	34	95	44	105	43
	964	984	929	984	853	1051
1.0	108	53	154	66	135	55
	1013	878	963	869	911	951
1.5	162	62	257	106	196	82
	1013	815	936	753	929	845
2.0	279	103	414	168	308	140
	981	689	846	624	885	719
3.0	564	208	769	316	582	231
	773	507	580	387	717	522
4.0	902	335	1099	476	903	371
	477	338	283	194	462	316
5.0	1150	474	1285	560	1165	486
	253	175	118	89	229	172

Table S90. Evaluation metrics across n (Cohort 3) (Survival).

Method	n	Accuracy	Error Rate	Precision	Recall	Specificity	F1	Kappa
RSF	0.5	0.514	0.486	0.673	0.068	0.967	0.123	0.034
	1.0	0.481	0.519	0.671	0.096	0.943	0.168	0.036
	1.5	0.476	0.524	0.723	0.138	0.929	0.232	0.059
	2.0	0.472	0.528	0.730	0.221	0.870	0.340	0.076
	3.0	0.522	0.478	0.731	0.422	0.709	0.535	0.111
	4.0	0.604	0.396	0.729	0.654	0.502	0.690	0.148
	5.0	0.646	0.354	0.708	0.820	0.270	0.760	0.098
Deepsurv	0.5	0.526	0.474	0.683	0.093	0.957	0.163	0.050
	1.0	0.499	0.501	0.700	0.138	0.929	0.230	0.062
	1.5	0.492	0.508	0.708	0.215	0.877	0.330	0.081
	2.0	0.506	0.494	0.711	0.329	0.788	0.450	0.100
	3.0	0.563	0.437	0.709	0.570	0.550	0.632	0.111
	4.0	0.630	0.370	0.698	0.795	0.290	0.743	0.092
	5.0	0.670	0.330	0.696	0.916	0.137	0.791	0.065
Cox	0.5	0.563	0.437	0.709	0.110	0.961	0.190	0.074
	1.0	0.529	0.471	0.711	0.129	0.945	0.218	0.073
	1.5	0.507	0.493	0.705	0.174	0.912	0.279	0.079
	2.0	0.500	0.500	0.688	0.258	0.837	0.375	0.085
	3.0	0.538	0.462	0.716	0.448	0.693	0.551	0.124
	4.0	0.594	0.406	0.709	0.662	0.460	0.684	0.118
	5.0	0.652	0.348	0.706	0.836	0.261	0.765	0.108

Table S91. Confusion matrices across n (Cohort 4) (Survival).

n	RSF		Deepsurv		Cox	
0.5	0	0	0	0	1	2
	388	642	391	639	359	668
1.0	3	1	2	1	4	2
	471	555	483	544	441	583
1.5	27	5	26	5	21	10
	543	455	547	452	530	469
2.0	79	25	97	31	89	35
	548	378	533	369	521	385
3.0	389	128	468	158	375	117
	327	186	251	153	337	201
4.0	674	193	727	220	658	191
	102	61	61	22	113	68
5.0	783	211	807	213	791	209
	31	5	8	2	19	11

Table S92. Evaluation metrics across n (Cohort 4) (Survival).

Method	n	Accuracy	Error Rate	Precision	Recall	Specificity	F1	Kappa
RSF	0.5	0.623	0.377		0.000	1.000		0.000
	1.0	0.542	0.458	0.750	0.006	0.998	0.013	0.005
	1.5	0.468	0.532	0.844	0.047	0.989	0.090	0.033
	2.0	0.444	0.556	0.760	0.126	0.938	0.216	0.052
	3.0	0.558	0.442	0.752	0.543	0.592	0.631	0.115
	4.0	0.714	0.286	0.777	0.869	0.240	0.820	0.124
	5.0	0.765	0.235	0.788	0.962	0.023	0.866	0.022
Deepsurv	0.5	0.620	0.380		0.000	1.000		0.000
	1.0	0.530	0.470	0.667	0.004	0.998	0.008	0.002
	1.5	0.464	0.536	0.839	0.045	0.989	0.086	0.031
	2.0	0.452	0.548	0.758	0.154	0.922	0.256	0.062
	3.0	0.603	0.397	0.748	0.651	0.492	0.696	0.132
	4.0	0.727	0.273	0.768	0.923	0.091	0.838	0.017
	5.0	0.785	0.215	0.791	0.990	0.009	0.880	0.001
Cox	0.5	0.650	0.350	0.333	0.003	0.997	0.006	0.000
	1.0	0.570	0.430	0.667	0.009	0.997	0.018	0.006
	1.5	0.476	0.524	0.677	0.038	0.979	0.072	0.016
	2.0	0.460	0.540	0.718	0.146	0.917	0.243	0.053
	3.0	0.559	0.441	0.762	0.527	0.632	0.623	0.133
	4.0	0.705	0.295	0.775	0.853	0.263	0.812	0.129
	5.0	0.779	0.221	0.791	0.977	0.050	0.874	0.039

Table S93. Confusion matrices across n (Cohort 5) (Survival).

n	RSF		Deepsurv		Cox	
0.5	0	0	2	0	2	0
	932	2399	935	2394	908	2421
1.0	0	0	4	0	2	0
	1113	2218	1129	2198	1077	2252
1.5	0	1	10	6	4	2
	1318	2012	1318	1997	1281	2044
2.0	1	4	43	21	14	9
	1488	1838	1464	1803	1441	1867
3.0	81	28	230	100	104	59
	1694	1528	1564	1437	1625	1543
4.0	496	196	666	295	408	189
	1476	1163	1315	1055	1526	1208
5.0	1166	472	1255	539	1009	454
	895	798	822	715	1031	837

Table S94. Evaluation metrics across n (Cohort 5) (Survival).

Method	n	Accuracy	Error Rate	Precision	Recall	Specificity	F1	Kappa
RSF	0.5	0.720	0.280		0.000	1.000		0.000
	1.0	0.666	0.334		0.000	1.000		0.000
	1.5	0.604	0.396	0.000	0.000	1.000		0.001
	2.0	0.552	0.448	0.200	0.001	0.998	0.001	0.002
	3.0	0.483	0.517	0.743	0.046	0.982	0.086	0.026
	4.0	0.498	0.502	0.717	0.252	0.856	0.372	0.094
	5.0	0.590	0.410	0.712	0.566	0.628	0.630	0.182
Deepsurv	0.5	0.719	0.281	1.000	0.002	1.000	0.004	0.003
	1.0	0.661	0.339	1.000	0.004	1.000	0.007	0.005
	1.5	0.603	0.397	0.625	0.008	0.997	0.015	0.005
	2.0	0.554	0.446	0.672	0.029	0.988	0.055	0.019
	3.0	0.500	0.500	0.697	0.128	0.935	0.217	0.059
	4.0	0.517	0.483	0.693	0.336	0.781	0.453	0.105
	5.0	0.591	0.409	0.700	0.604	0.570	0.648	0.167
Cox	0.5	0.727	0.273	1.000	0.002	1.000	0.004	0.003
	1.0	0.677	0.323	1.000	0.002	1.000	0.004	0.003
	1.5	0.615	0.385	0.667	0.003	0.999	0.006	0.003
	2.0	0.565	0.435	0.609	0.010	0.995	0.019	0.005
	3.0	0.494	0.506	0.638	0.060	0.963	0.110	0.023
	4.0	0.485	0.515	0.683	0.211	0.865	0.322	0.067
	5.0	0.554	0.446	0.690	0.495	0.648	0.576	0.132

Table S95. Confusion matrices across n (Cohort 6) (Survival).

n	RSF		Deepsurv		Cox	
0.5	0	0	0	0	1	1
	284	1145	296	1133	290	1137
1.0	0	0	0	0	1	1
	410	1019	413	1016	404	1023
1.5	0	0	0	0	1	3
	528	901	541	888	535	890
2.0	1	1	1	0	6	3
	631	796	642	786	632	788
3.0	58	29	91	44	101	44
	747	595	721	573	712	572
4.0	360	122	492	184	445	149
	590	357	471	282	502	333
5.0	715	229	847	288	802	261
	316	169	189	105	228	138

Table S96. Evaluation metrics across n (Cohort 6) (Survival).

Method	n	Accuracy	Error Rate	Precision	Recall	Specificity	F1	Kappa
RSF	0.5	0.801	0.199		0.000	1.000		0.000
	1.0	0.713	0.287		0.000	1.000		0.000
	1.5	0.631	0.369		0.000	1.000		0.000
	2.0	0.558	0.442	0.500	0.002	0.999	0.003	0.000
	3.0	0.457	0.543	0.667	0.072	0.954	0.130	0.023
	4.0	0.502	0.498	0.747	0.379	0.745	0.503	0.100
	5.0	0.619	0.381	0.757	0.694	0.425	0.724	0.111
Deepsurv	0.5	0.793	0.207		0.000	1.000		0.000
	1.0	0.711	0.289		0.000	1.000		0.000
	1.5	0.621	0.379		0.000	1.000		0.000
	2.0	0.551	0.449	1.000	0.002	1.000	0.003	0.002
	3.0	0.465	0.535	0.674	0.112	0.929	0.192	0.036
	4.0	0.542	0.458	0.728	0.511	0.605	0.600	0.100
	5.0	0.666	0.334	0.746	0.818	0.267	0.780	0.092
Cox	0.5	0.796	0.204	0.500	0.003	0.999	0.007	0.004
	1.0	0.717	0.283	0.500	0.002	0.999	0.005	0.002
	1.5	0.624	0.376	0.250	0.002	0.997	0.004	0.002
	2.0	0.556	0.444	0.667	0.009	0.996	0.019	0.006
	3.0	0.471	0.529	0.697	0.124	0.929	0.211	0.047
	4.0	0.544	0.456	0.749	0.470	0.691	0.578	0.136
	5.0	0.658	0.342	0.754	0.779	0.346	0.766	0.128

Table S97. Confusion matrices across n (Cohort 7) (Survival).

n	RSF		Deepsurv		Cox	
0.5	4	4	0	0	12	6
	349	617	362	612	319	637
1.0	6	5	0	1	17	9
	403	560	420	553	370	578
1.5	13	7	5	6	23	17
	444	510	457	506	420	514
2.0	27	10	10	11	55	27
	469	468	492	461	421	471
3.0	86	37	86	55	131	55
	473	378	480	353	405	383
4.0	236	121	295	200	251	114
	358	259	302	177	335	274
5.0	439	224	520	309	397	182
	176	135	96	49	216	179

Table S98. Evaluation metrics across n (Cohort 7) (Survival).

Method	n	Accuracy	Error Rate	Precision	Recall	Specificity	F1	Kappa
RSF	0.5	0.638	0.362	0.500	0.011	0.994	0.022	0.006
	1.0	0.581	0.419	0.545	0.015	0.991	0.029	0.007
	1.5	0.537	0.463	0.650	0.028	0.986	0.055	0.016
	2.0	0.508	0.492	0.730	0.054	0.979	0.101	0.033
	3.0	0.476	0.524	0.699	0.154	0.911	0.252	0.057
	4.0	0.508	0.492	0.661	0.397	0.682	0.496	0.071
	5.0	0.589	0.411	0.662	0.714	0.376	0.687	0.092
Deepsurv	0.5	0.628	0.372		0.000	1.000		0.000
	1.0	0.568	0.432	0.000	0.000	0.998		0.002
	1.5	0.525	0.475	0.455	0.011	0.988	0.021	0.001
	2.0	0.484	0.516	0.476	0.020	0.977	0.038	0.003
	3.0	0.451	0.549	0.610	0.152	0.865	0.243	0.015
	4.0	0.485	0.515	0.596	0.494	0.469	0.540	0.035
	5.0	0.584	0.416	0.627	0.844	0.137	0.720	0.022
Cox	0.5	0.666	0.334	0.667	0.036	0.991	0.069	0.035
	1.0	0.611	0.389	0.654	0.044	0.985	0.082	0.034
	1.5	0.551	0.449	0.575	0.052	0.968	0.095	0.022
	2.0	0.540	0.460	0.671	0.116	0.946	0.197	0.062
	3.0	0.528	0.472	0.704	0.244	0.874	0.363	0.111
	4.0	0.539	0.461	0.688	0.428	0.706	0.528	0.123
	5.0	0.591	0.409	0.686	0.648	0.496	0.666	0.141

Table S99. Confusion matrices across n (Cohort 8) (Survival).

n	RSF		Deepsurv		Cox	
0.5	1	1	0	0	2	3
	173	386	176	385	166	390
1.0	2	1	0	0	3	3
	210	348	215	346	208	347
1.5	4	2	1	1	10	11
	251	304	259	300	239	301
2.0	17	7	11	3	40	21
	274	263	284	263	242	258
3.0	122	47	123	61	146	60
	234	158	237	140	206	149
4.0	339	125	355	142	283	95
	62	35	50	14	117	66
5.0	433	124	438	123	389	103
	3	1	0	0	43	26

Table S100. Evaluation metrics across n (Cohort 8) (Survival).

Method	n	Accuracy	Error Rate	Precision	Recall	Specificity	F1	Kappa
RSF	0.5	0.690	0.310	0.500	0.006	0.997	0.011	0.004
	1.0	0.624	0.376	0.667	0.009	0.997	0.019	0.008
	1.5	0.549	0.451	0.667	0.016	0.993	0.031	0.010
	2.0	0.499	0.501	0.708	0.058	0.974	0.108	0.031
	3.0	0.499	0.501	0.722	0.343	0.771	0.465	0.095
	4.0	0.667	0.333	0.731	0.845	0.219	0.784	0.073
	5.0	0.774	0.226	0.777	0.993	0.008	0.872	0.002
Deepsurv	0.5	0.686	0.314		0.000	1.000		0.000
	1.0	0.617	0.383		0.000	1.000		0.000
	1.5	0.537	0.463	0.500	0.004	0.997	0.008	0.001
	2.0	0.488	0.512	0.786	0.037	0.989	0.071	0.025
	3.0	0.469	0.531	0.668	0.342	0.697	0.452	0.032
	4.0	0.658	0.342	0.714	0.877	0.090	0.787	0.041
	5.0	0.781	0.219	0.781	1.000	0.000	0.877	0.000
Cox	0.5	0.699	0.301	0.400	0.012	0.992	0.023	0.006
	1.0	0.624	0.376	0.500	0.014	0.991	0.028	0.007
	1.5	0.554	0.446	0.476	0.040	0.965	0.074	0.005
	2.0	0.531	0.469	0.656	0.142	0.925	0.233	0.066
	3.0	0.526	0.474	0.709	0.415	0.713	0.523	0.112
	4.0	0.622	0.378	0.749	0.708	0.410	0.728	0.113
	5.0	0.740	0.260	0.791	0.900	0.202	0.842	0.122

Table S101. Confusion matrices across n (Cohort 9) (Survival).

n	RSF		Deepsurv		Cox	
0.5	4	5	7	3	13	5
	899	1606	889	1615	844	1652
1.0	8	6	20	6	23	8
	1033	1467	998	1490	965	1518
1.5	20	11	50	17	42	17
	1137	1346	1114	1333	1077	1378
2.0	66	28	124	48	103	34
	1197	1223	1140	1202	1123	1254
3.0	276	116	464	188	295	125
	1179	943	987	875	1113	981
4.0	737	314	894	388	703	303
	813	650	654	578	830	678
5.0	1156	543	1219	541	1125	512
	440	375	378	376	463	414

Table S102. Evaluation metrics across n (Cohort 9) (Survival).

Method	n	Accuracy	Error Rate	Precision	Recall	Specificity	F1	Kappa
RSF	0.5	0.640	0.360	0.444	0.004	0.997	0.009	0.002
	1.0	0.587	0.413	0.571	0.008	0.996	0.015	0.004
	1.5	0.543	0.457	0.645	0.017	0.992	0.034	0.010
	2.0	0.513	0.487	0.702	0.052	0.978	0.097	0.030
	3.0	0.485	0.515	0.704	0.190	0.890	0.299	0.071
	4.0	0.552	0.448	0.701	0.475	0.674	0.567	0.136
	5.0	0.609	0.391	0.680	0.724	0.408	0.702	0.136
Deepsurv	0.5	0.645	0.355	0.700	0.008	0.998	0.015	0.008
	1.0	0.601	0.399	0.769	0.020	0.996	0.038	0.019
	1.5	0.550	0.450	0.746	0.043	0.987	0.081	0.032
	2.0	0.527	0.473	0.721	0.098	0.962	0.173	0.059
	3.0	0.533	0.467	0.712	0.320	0.823	0.441	0.130
	4.0	0.586	0.414	0.697	0.578	0.598	0.632	0.167
	5.0	0.634	0.366	0.693	0.763	0.410	0.726	0.180
Cox	0.5	0.662	0.338	0.722	0.015	0.997	0.030	0.016
	1.0	0.613	0.387	0.742	0.023	0.995	0.045	0.022
	1.5	0.565	0.435	0.712	0.038	0.988	0.071	0.028
	2.0	0.540	0.460	0.752	0.084	0.974	0.151	0.059
	3.0	0.508	0.492	0.702	0.210	0.887	0.323	0.088
	4.0	0.549	0.451	0.699	0.459	0.691	0.554	0.137
	5.0	0.612	0.388	0.687	0.708	0.447	0.698	0.157

Table S103. Confusion matrices across n (Cohort 10) (Survival).

n	RSF		Deepsurv		Cox	
0.5	0	0	0	2	3	7
	229	619	240	606	237	601
1.0	0	0	6	2	8	11
	298	550	299	541	294	535
1.5	1	1	13	3	23	17
	358	488	361	471	350	458
2.0	9	3	33	13	58	27
	407	429	391	411	375	388
3.0	136	42	160	54	178	67
	380	290	361	273	341	262
4.0	335	109	372	129	371	137
	243	161	215	132	219	121
5.0	524	153	553	166	528	148
	106	65	79	50	103	69

Table S104. Evaluation metrics across n (Cohort 10) (Survival).

Method	n	Accuracy	Error Rate	Precision	Recall	Specificity	F1	Kappa
RSF	0.5	0.730	0.270		0.000	1.000		0.000
	1.0	0.649	0.351		0.000	1.000		0.000
	1.5	0.577	0.423	0.500	0.003	0.998	0.006	0.001
	2.0	0.517	0.483	0.750	0.022	0.993	0.042	0.015
	3.0	0.502	0.498	0.764	0.264	0.873	0.392	0.116
	4.0	0.585	0.415	0.755	0.580	0.596	0.656	0.155
	5.0	0.695	0.305	0.774	0.832	0.298	0.802	0.140
Deepsurv	0.5	0.715	0.285	0.000	0.000	0.997		0.005
	1.0	0.645	0.355	0.750	0.020	0.996	0.038	0.020
	1.5	0.571	0.429	0.812	0.035	0.994	0.067	0.032
	2.0	0.524	0.476	0.717	0.078	0.969	0.140	0.047
	3.0	0.511	0.489	0.748	0.307	0.835	0.435	0.121
	4.0	0.594	0.406	0.743	0.634	0.506	0.684	0.128
	5.0	0.711	0.289	0.769	0.875	0.231	0.819	0.123
Cox	0.5	0.712	0.288	0.300	0.012	0.988	0.024	0.001
	1.0	0.640	0.360	0.421	0.026	0.980	0.050	0.008
	1.5	0.567	0.433	0.575	0.062	0.964	0.111	0.029
	2.0	0.526	0.474	0.682	0.134	0.935	0.224	0.068
	3.0	0.519	0.481	0.727	0.343	0.796	0.466	0.121
	4.0	0.580	0.420	0.730	0.629	0.469	0.676	0.090
	5.0	0.704	0.296	0.781	0.837	0.318	0.808	0.166

Table S105. Confusion matrices across n (Cohort 11) (Survival).

n	RSF		Deepsurv		Cox	
0.5	0	0	0	0	1	0
	113	716	108	721	107	721
1.0	0	0	0	0	1	1
	156	673	143	686	140	687
1.5	0	0	0	0	4	8
	192	637	188	641	173	644
2.0	0	0	1	0	12	15
	236	593	222	606	207	595
3.0	9	5	18	11	51	34
	297	518	279	521	244	500
4.0	70	17	70	25	112	47
	308	434	305	429	263	407
5.0	154	40	167	41	189	54
	279	356	263	358	240	346

Table S106. Evaluation metrics across n (Cohort 11) (Survival).

Method	n	Accuracy	Error Rate	Precision	Recall	Specificity	F1	Kappa
RSF	0.5	0.864	0.136		0.000	1.000		0.000
	1.0	0.812	0.188		0.000	1.000		0.000
	1.5	0.768	0.232		0.000	1.000		0.000
	2.0	0.715	0.285		0.000	1.000		0.000
	3.0	0.636	0.364	0.643	0.029	0.990	0.056	0.025
	4.0	0.608	0.392	0.805	0.185	0.962	0.301	0.157
	5.0	0.615	0.385	0.794	0.356	0.899	0.491	0.248
Deepsurv	0.5	0.870	0.130		0.000	1.000		0.000
	1.0	0.828	0.172		0.000	1.000		0.000
	1.5	0.773	0.227		0.000	1.000		0.000
	2.0	0.732	0.268	1.000	0.004	1.000	0.009	0.007
	3.0	0.650	0.350	0.621	0.061	0.979	0.110	0.050
	4.0	0.602	0.398	0.737	0.187	0.945	0.298	0.141
	5.0	0.633	0.367	0.803	0.388	0.897	0.524	0.280
Cox	0.5	0.871	0.129	1.000	0.009	1.000	0.018	0.016
	1.0	0.830	0.170	0.500	0.007	0.999	0.014	0.009
	1.5	0.782	0.218	0.333	0.023	0.988	0.042	0.016
	2.0	0.732	0.268	0.444	0.055	0.975	0.098	0.042
	3.0	0.665	0.335	0.600	0.173	0.936	0.268	0.130
	4.0	0.626	0.374	0.704	0.299	0.896	0.419	0.205
	5.0	0.645	0.355	0.778	0.441	0.865	0.562	0.301

Table S107. Confusion matrices across n (Cohort 0) (Classification).

n	GBM		RF		LB	
0.5	8169	7458	8293	7868	10420	14785
	3214	11193	3090	10783	963	3866
1.0	8482	6432	8448	6615	7427	7154
	3886	11234	3920	11051	4941	10512
1.5	9307	6312	9224	6373	8384	6721
	3914	10501	3997	10440	4837	10092
2.0	10280	6261	9817	5845	8414	6167
	3811	9682	4274	10098	5677	9776
3.0	10800	5691	10866	5865	8914	5667
	4156	9387	4090	9213	6042	9411
4.0	11098	5510	10445	4923	9135	5446
	4267	9159	4920	9746	6230	9223
5.0	11087	4914	11153	5092	9361	4925
	4728	9305	4662	9127	6454	9294

Table S108. Evaluation metrics across n (Cohort 0) (Classification).

Method	n	Accuracy	Error Rate	Precision	Recall	Specificity	F1	Kappa
GBM	0.5	0.645	0.355	0.523	0.718	0.600	0.605	0.645
	1.0	0.656	0.344	0.569	0.686	0.636	0.622	0.656
	1.5	0.660	0.340	0.596	0.704	0.625	0.645	0.660
	2.0	0.665	0.335	0.621	0.730	0.607	0.671	0.665
	3.0	0.672	0.328	0.655	0.722	0.623	0.687	0.672
	4.0	0.674	0.326	0.668	0.722	0.624	0.694	0.674
	5.0	0.679	0.321	0.693	0.701	0.654	0.697	0.679
RF	0.5	0.635	0.365	0.513	0.729	0.578	0.602	0.635
	1.0	0.649	0.351	0.561	0.683	0.626	0.616	0.649
	1.5	0.655	0.345	0.591	0.698	0.621	0.640	0.655
	2.0	0.663	0.337	0.627	0.697	0.633	0.660	0.663
	3.0	0.669	0.331	0.649	0.727	0.611	0.686	0.669
	4.0	0.672	0.328	0.680	0.680	0.664	0.680	0.672
	5.0	0.675	0.325	0.687	0.705	0.642	0.696	0.675
LB	0.5	0.476	0.524	0.413	0.915	0.207	0.570	0.476
	1.0	0.597	0.403	0.509	0.601	0.595	0.551	0.597
	1.5	0.615	0.385	0.555	0.634	0.600	0.592	0.615
	2.0	0.606	0.394	0.577	0.597	0.613	0.587	0.606
	3.0	0.610	0.390	0.611	0.596	0.624	0.604	0.610
	4.0	0.611	0.389	0.627	0.595	0.629	0.610	0.611
	5.0	0.621	0.379	0.655	0.592	0.654	0.622	0.621

Table S109. Confusion matrices across n (Cohort 1) (Classification).

n	GBM		RF		LB	
0.5	1412	1555	1275	1461	228	171
	613	1796	750	1890	1797	3180
1.0	1656	1509	1603	1542	458	371
	647	1564	700	1531	1845	2702
1.5	1707	1295	1818	1461	1367	1328
	829	1545	718	1379	1169	1512
2.0	1571	895	1966	1323	1782	1403
	1232	1678	837	1250	1021	1170
3.0	1929	823	1922	899	3028	1933
	1276	1348	1283	1272	177	238
4.0	1925	689	2149	876	3216	1765
	1470	1292	1246	1105	179	216
5.0	2373	854	1970	676	3296	1677
	1114	1035	1517	1213	191	212

Table S110. Evaluation metrics across n (Cohort 1) (Classification).

Method	n	Accuracy	Error Rate	Precision	Recall	Specificity	F1	Kappa
GBM	0.5	0.597	0.403	0.476	0.697	0.536	0.566	0.597
	1.0	0.599	0.401	0.523	0.719	0.509	0.606	0.599
	1.5	0.605	0.395	0.569	0.673	0.544	0.616	0.605
	2.0	0.604	0.396	0.637	0.560	0.652	0.596	0.604
	3.0	0.610	0.390	0.701	0.602	0.621	0.648	0.610
	4.0	0.598	0.402	0.736	0.567	0.652	0.641	0.598
	5.0	0.634	0.366	0.735	0.681	0.548	0.707	0.634
RF	0.5	0.589	0.411	0.466	0.630	0.564	0.536	0.589
	1.0	0.583	0.417	0.510	0.696	0.498	0.588	0.583
	1.5	0.595	0.405	0.554	0.717	0.486	0.625	0.595
	2.0	0.598	0.402	0.598	0.701	0.486	0.645	0.598
	3.0	0.594	0.406	0.681	0.600	0.586	0.638	0.594
	4.0	0.605	0.395	0.710	0.633	0.558	0.669	0.605
	5.0	0.592	0.408	0.745	0.565	0.642	0.642	0.592
LB	0.5	0.634	0.366	0.571	0.113	0.949	0.188	0.634
	1.0	0.588	0.412	0.552	0.199	0.879	0.292	0.588
	1.5	0.536	0.464	0.507	0.539	0.532	0.523	0.536
	2.0	0.549	0.451	0.559	0.636	0.455	0.595	0.549
	3.0	0.608	0.392	0.610	0.945	0.110	0.742	0.608
	4.0	0.638	0.362	0.646	0.947	0.109	0.768	0.638
	5.0	0.653	0.347	0.663	0.945	0.112	0.779	0.653

Table S111. Confusion matrices across n (Cohort 2) (Classification).

n	GBM		RF		LB	
0.5	392	522	486	826	604	1209
	327	1228	233	924	115	541
1.0	661	731	579	664	121	114
	258	819	340	886	798	1436
1.5	812	664	770	652	389	288
	310	683	352	695	733	1059
2.0	962	609	994	670	726	487
	333	565	301	504	569	687
3.0	1003	397	985	448	1484	858
	541	528	559	477	60	67
4.0	877	233	790	219	340	81
	860	499	947	513	1397	651
5.0	1122	258	972	232	666	178
	729	360	879	386	1185	440

Table S112. Evaluation metrics across n (Cohort 2) (Classification).

Method	n	Accuracy	Error Rate	Precision	Recall	Specificity	F1	Kappa
GBM	0.5	0.656	0.344	0.429	0.545	0.702	0.480	0.656
	1.0	0.599	0.401	0.475	0.719	0.528	0.572	0.599
	1.5	0.606	0.394	0.550	0.724	0.507	0.625	0.606
	2.0	0.618	0.382	0.612	0.743	0.481	0.671	0.618
	3.0	0.620	0.380	0.716	0.650	0.571	0.681	0.620
	4.0	0.557	0.443	0.790	0.505	0.682	0.616	0.557
	5.0	0.600	0.400	0.813	0.606	0.583	0.695	0.600
RF	0.5	0.571	0.429	0.370	0.676	0.528	0.479	0.571
	1.0	0.593	0.407	0.466	0.630	0.572	0.536	0.593
	1.5	0.593	0.407	0.541	0.686	0.516	0.605	0.593
	2.0	0.607	0.393	0.597	0.768	0.429	0.672	0.607
	3.0	0.592	0.408	0.687	0.638	0.516	0.662	0.592
	4.0	0.528	0.472	0.783	0.455	0.701	0.575	0.528
	5.0	0.550	0.450	0.807	0.525	0.625	0.636	0.550
LB	0.5	0.464	0.536	0.333	0.840	0.309	0.477	0.464
	1.0	0.631	0.369	0.515	0.132	0.926	0.210	0.631
	1.5	0.586	0.414	0.575	0.347	0.786	0.432	0.586
	2.0	0.572	0.428	0.599	0.561	0.585	0.579	0.572
	3.0	0.628	0.372	0.634	0.961	0.072	0.764	0.628
	4.0	0.401	0.599	0.808	0.196	0.889	0.315	0.401
	5.0	0.448	0.552	0.789	0.360	0.712	0.494	0.448

Table S113. Confusion matrices across n (Cohort 3) (Classification).

n	GBM		RF		LB	
0.5	685	419	605	339	547	412
	393	555	473	635	531	562
1.0	788	434	690	338	1017	762
	362	468	460	564	133	140
1.5	703	294	711	323	1123	701
	514	541	506	512	94	134
2.0	739	266	786	307	104	23
	548	499	501	458	1183	742
3.0	826	264	663	205	147	40
	525	437	688	496	1204	661
4.0	875	261	907	291	277	76
	504	412	472	382	1102	597
5.0	1014	300	800	218	344	105
	387	351	601	433	1057	546

Table S114. Evaluation metrics across n (Cohort 3) (Classification).

Method	n	Accuracy	Error Rate	Precision	Recall	Specificity	F1	Kappa
GBM	0.5	0.604	0.396	0.620	0.635	0.570	0.628	0.604
	1.0	0.612	0.388	0.645	0.685	0.519	0.664	0.612
	1.5	0.606	0.394	0.705	0.578	0.648	0.635	0.606
	2.0	0.603	0.397	0.735	0.574	0.652	0.645	0.603
	3.0	0.615	0.385	0.758	0.611	0.623	0.677	0.615
	4.0	0.627	0.373	0.770	0.635	0.612	0.696	0.627
	5.0	0.665	0.335	0.772	0.724	0.539	0.747	0.665
RF	0.5	0.604	0.396	0.641	0.561	0.652	0.598	0.604
	1.0	0.611	0.389	0.671	0.600	0.625	0.634	0.611
	1.5	0.596	0.404	0.688	0.584	0.613	0.632	0.596
	2.0	0.606	0.394	0.719	0.611	0.599	0.661	0.606
	3.0	0.565	0.435	0.764	0.491	0.708	0.598	0.565
	4.0	0.628	0.372	0.757	0.658	0.568	0.704	0.628
	5.0	0.601	0.399	0.786	0.571	0.665	0.661	0.601
LB	0.5	0.540	0.460	0.570	0.507	0.577	0.537	0.540
	1.0	0.564	0.436	0.572	0.884	0.155	0.694	0.564
	1.5	0.613	0.387	0.616	0.923	0.160	0.739	0.613
	2.0	0.412	0.588	0.819	0.081	0.970	0.147	0.412
	3.0	0.394	0.606	0.786	0.109	0.943	0.191	0.394
	4.0	0.426	0.574	0.785	0.201	0.887	0.320	0.426
	5.0	0.434	0.566	0.766	0.246	0.839	0.372	0.434

Table S115. Confusion matrices across n (Cohort 4) (Classification).

n	GBM		RF		LB	
0.5	204	239	250	315	372	631
	177	410	131	334	9	18
1.0	268	210	281	200	99	94
	211	341	198	351	380	457
1.5	358	191	306	145	543	416
	216	265	268	311	31	40
2.0	356	150	348	155	600	356
	280	244	288	239	36	38
3.0	287	66	391	125	15	3
	428	249	324	190	700	312
4.0	482	108	362	87	637	198
	297	143	417	164	142	53
5.0	460	87	419	81	34	2
	355	128	396	134	781	213

Table S116. Evaluation metrics across n (Cohort 4) (Classification).

Method	n	Accuracy	Error Rate	Precision	Recall	Specificity	F1	Kappa
GBM	0.5	0.596	0.404	0.460	0.535	0.632	0.495	0.596
	1.0	0.591	0.409	0.561	0.559	0.619	0.560	0.591
	1.5	0.605	0.395	0.652	0.624	0.581	0.638	0.605
	2.0	0.583	0.417	0.704	0.560	0.619	0.623	0.583
	3.0	0.520	0.480	0.813	0.401	0.790	0.537	0.520
	4.0	0.607	0.393	0.817	0.619	0.570	0.704	0.607
	5.0	0.571	0.429	0.841	0.564	0.595	0.675	0.571
RF	0.5	0.567	0.433	0.442	0.656	0.515	0.529	0.567
	1.0	0.614	0.386	0.584	0.587	0.637	0.585	0.614
	1.5	0.599	0.401	0.678	0.533	0.682	0.597	0.599
	2.0	0.570	0.430	0.692	0.547	0.607	0.611	0.570
	3.0	0.564	0.436	0.758	0.547	0.603	0.635	0.564
	4.0	0.511	0.489	0.806	0.465	0.653	0.590	0.511
	5.0	0.537	0.463	0.838	0.514	0.623	0.637	0.537
LB	0.5	0.379	0.621	0.371	0.976	0.028	0.538	0.379
	1.0	0.540	0.460	0.513	0.207	0.829	0.295	0.540
	1.5	0.566	0.434	0.566	0.946	0.088	0.708	0.566
	2.0	0.619	0.381	0.628	0.943	0.096	0.754	0.619
	3.0	0.317	0.683	0.833	0.021	0.990	0.041	0.317
	4.0	0.670	0.330	0.763	0.818	0.211	0.789	0.670
	5.0	0.240	0.760	0.944	0.042	0.991	0.080	0.240

Table S117. Confusion matrices across n (Cohort 5) (Classification).

n	GBM		RF		LB	
0.5	757	1294	610	1029	811	1688
	228	1052	375	1317	174	658
1.0	739	846	688	818	1121	1994
	437	1309	488	1337	55	161
1.5	857	787	857	874	186	172
	475	1212	475	1125	1146	1827
2.0	1035	831	959	772	296	245
	474	991	550	1050	1213	1577
3.0	1322	770	1228	696	1388	955
	498	741	592	815	432	556
4.0	1199	492	1310	601	1845	1144
	805	835	694	726	159	183
5.0	1424	547	1295	505	1969	1114
	658	702	787	744	113	135

Table S118. Evaluation metrics across n (Cohort 5) (Classification).

Method	n	Accuracy	Error Rate	Precision	Recall	Specificity	F1	Kappa
GBM	0.5	0.543	0.457	0.369	0.769	0.448	0.499	0.543
	1.0	0.615	0.385	0.466	0.628	0.607	0.535	0.615
	1.5	0.621	0.379	0.521	0.643	0.606	0.576	0.621
	2.0	0.608	0.392	0.555	0.686	0.544	0.613	0.608
	3.0	0.619	0.381	0.632	0.726	0.490	0.676	0.619
	4.0	0.611	0.389	0.709	0.598	0.629	0.649	0.611
	5.0	0.638	0.362	0.722	0.684	0.562	0.703	0.638
RF	0.5	0.579	0.421	0.372	0.619	0.561	0.465	0.579
	1.0	0.608	0.392	0.457	0.585	0.620	0.513	0.608
	1.5	0.595	0.405	0.495	0.643	0.563	0.560	0.595
	2.0	0.603	0.397	0.554	0.636	0.576	0.592	0.603
	3.0	0.613	0.387	0.638	0.675	0.539	0.656	0.613
	4.0	0.611	0.389	0.686	0.654	0.547	0.669	0.611
	5.0	0.612	0.388	0.719	0.622	0.596	0.667	0.612
LB	0.5	0.441	0.559	0.325	0.823	0.280	0.466	0.441
	1.0	0.385	0.615	0.360	0.953	0.075	0.522	0.385
	1.5	0.604	0.396	0.520	0.140	0.914	0.220	0.604
	2.0	0.562	0.438	0.547	0.196	0.866	0.289	0.562
	3.0	0.584	0.416	0.592	0.763	0.368	0.667	0.584
	4.0	0.609	0.391	0.617	0.921	0.138	0.739	0.609
	5.0	0.632	0.368	0.639	0.946	0.108	0.762	0.632

Table S119. Confusion matrices across n (Cohort 6) (Classification).

n	GBM		RF		LB	
0.5	165	343	166	418	288	959
	146	775	145	700	23	159
1.0	248	380	210	342	342	699
	176	625	214	663	82	306
1.5	339	377	335	368	86	73
	199	514	203	523	452	818
2.0	379	333	407	346	225	185
	262	455	234	442	416	603
3.0	615	376	586	344	746	530
	193	245	222	277	62	91
4.0	476	153	687	273	924	429
	476	324	265	204	28	48
5.0	619	152	632	182	1002	349
	419	239	406	209	36	42

Table S120. Evaluation metrics across n (Cohort 6) (Classification).

Method	n	Accuracy	Error Rate	Precision	Recall	Specificity	F1	Kappa
GBM	0.5	0.658	0.342	0.325	0.531	0.693	0.403	0.658
	1.0	0.611	0.389	0.395	0.585	0.622	0.471	0.611
	1.5	0.597	0.403	0.473	0.630	0.577	0.541	0.597
	2.0	0.584	0.416	0.532	0.591	0.577	0.560	0.584
	3.0	0.602	0.398	0.621	0.761	0.395	0.684	0.602
	4.0	0.560	0.440	0.757	0.500	0.679	0.602	0.560
	5.0	0.600	0.400	0.803	0.596	0.611	0.684	0.600
RF	0.5	0.606	0.394	0.284	0.534	0.626	0.371	0.606
	1.0	0.611	0.389	0.380	0.495	0.660	0.430	0.611
	1.5	0.600	0.400	0.477	0.623	0.587	0.540	0.600
	2.0	0.594	0.406	0.541	0.635	0.561	0.584	0.594
	3.0	0.604	0.396	0.630	0.725	0.446	0.674	0.604
	4.0	0.624	0.376	0.716	0.722	0.428	0.719	0.624
	5.0	0.589	0.411	0.776	0.609	0.535	0.683	0.589
LB	0.5	0.313	0.687	0.231	0.926	0.142	0.370	0.313
	1.0	0.453	0.547	0.329	0.807	0.304	0.467	0.453
	1.5	0.633	0.367	0.541	0.160	0.918	0.247	0.633
	2.0	0.579	0.421	0.549	0.351	0.765	0.428	0.579
	3.0	0.586	0.414	0.585	0.923	0.147	0.716	0.586
	4.0	0.680	0.320	0.683	0.971	0.101	0.802	0.680
	5.0	0.731	0.269	0.742	0.965	0.107	0.839	0.731

Table S121. Confusion matrices across n (Cohort 7) (Classification).

n	GBM		RF		LB	
0.5	279	325	180	191	64	55
	120	331	219	465	335	601
1.0	291	269	218	188	100	98
	164	331	237	412	355	502
1.5	268	197	248	203	137	122
	222	368	242	362	353	443
2.0	279	159	307	217	332	288
	255	362	227	304	202	233
3.0	312	147	540	325	553	365
	300	296	72	118	59	78
4.0	359	158	477	250	639	399
	285	253	167	161	5	12
5.0	575	270	386	160	49	14
	91	119	280	229	617	375

Table S122. Evaluation metrics across n (Cohort 7) (Classification).

Method	n	Accuracy	Error Rate	Precision	Recall	Specificity	F1	Kappa
GBM	0.5	0.578	0.422	0.462	0.699	0.505	0.556	0.578
	1.0	0.590	0.410	0.520	0.640	0.552	0.573	0.590
	1.5	0.603	0.397	0.576	0.547	0.651	0.561	0.603
	2.0	0.608	0.392	0.637	0.522	0.695	0.574	0.608
	3.0	0.576	0.424	0.680	0.510	0.668	0.583	0.576
	4.0	0.580	0.420	0.694	0.557	0.616	0.618	0.580
	5.0	0.658	0.342	0.680	0.863	0.306	0.761	0.658
RF	0.5	0.611	0.389	0.485	0.451	0.709	0.468	0.611
	1.0	0.597	0.403	0.537	0.479	0.687	0.506	0.597
	1.5	0.578	0.422	0.550	0.506	0.641	0.527	0.578
	2.0	0.579	0.421	0.586	0.575	0.583	0.580	0.579
	3.0	0.624	0.376	0.624	0.882	0.266	0.731	0.624
	4.0	0.605	0.395	0.656	0.741	0.392	0.696	0.605
	5.0	0.583	0.417	0.707	0.580	0.589	0.637	0.583
LB	0.5	0.630	0.370	0.538	0.160	0.916	0.247	0.630
	1.0	0.571	0.429	0.505	0.220	0.837	0.306	0.571
	1.5	0.550	0.450	0.529	0.280	0.784	0.366	0.550
	2.0	0.536	0.464	0.535	0.622	0.447	0.575	0.536
	3.0	0.598	0.402	0.602	0.904	0.176	0.723	0.598
	4.0	0.617	0.383	0.616	0.992	0.029	0.760	0.617
	5.0	0.402	0.598	0.778	0.074	0.964	0.134	0.402

Table S123. Confusion matrices across n (Cohort 8) (Classification).

n	GBM		RF		LB	
0.5	93	143	117	218	55	71
	83	254	59	179	121	326
1.0	170	191	180	231	34	39
	53	159	43	119	189	311
1.5	224	186	180	161	61	58
	39	124	83	149	202	252
2.0	221	151	188	124	203	156
	70	131	103	158	88	126
3.0	230	98	201	85	78	35
	130	115	159	128	282	178
4.0	170	40	130	25	125	29
	253	110	293	125	298	121
5.0	255	53	198	40	108	22
	191	74	248	87	338	105

Table S124. Evaluation metrics across n (Cohort 8) (Classification).

Method	n	Accuracy	Error Rate	Precision	Recall	Specificity	F1	Kappa
GBM	0.5	0.606	0.394	0.394	0.528	0.640	0.451	0.606
	1.0	0.574	0.426	0.471	0.762	0.454	0.582	0.574
	1.5	0.607	0.393	0.546	0.852	0.400	0.666	0.607
	2.0	0.614	0.386	0.594	0.759	0.465	0.667	0.614
	3.0	0.602	0.398	0.701	0.639	0.540	0.669	0.602
	4.0	0.489	0.511	0.810	0.402	0.733	0.537	0.489
	5.0	0.574	0.426	0.828	0.572	0.583	0.676	0.574
RF	0.5	0.517	0.483	0.349	0.665	0.451	0.458	0.517
	1.0	0.522	0.478	0.438	0.807	0.340	0.568	0.522
	1.5	0.574	0.426	0.528	0.684	0.481	0.596	0.574
	2.0	0.604	0.396	0.603	0.646	0.560	0.624	0.604
	3.0	0.574	0.426	0.703	0.558	0.601	0.622	0.574
	4.0	0.445	0.555	0.839	0.307	0.833	0.450	0.445
	5.0	0.497	0.503	0.832	0.444	0.685	0.579	0.497
LB	0.5	0.665	0.335	0.437	0.312	0.821	0.364	0.665
	1.0	0.602	0.398	0.466	0.152	0.889	0.230	0.602
	1.5	0.546	0.454	0.513	0.232	0.813	0.319	0.546
	2.0	0.574	0.426	0.565	0.698	0.447	0.625	0.574
	3.0	0.447	0.553	0.690	0.217	0.836	0.330	0.447
	4.0	0.429	0.571	0.812	0.296	0.807	0.433	0.429
	5.0	0.372	0.628	0.831	0.242	0.827	0.375	0.372

Table S125. Confusion matrices across n (Cohort 9) (Classification).

n	GBM		RF		LB	
0.5	580	669	552	652	90	78
	255	729	283	746	745	1320
1.0	761	784	627	577	151	114
	175	513	309	720	785	1183
1.5	655	508	626	488	384	320
	378	692	407	712	649	880
2.0	799	528	798	531	833	705
	345	561	346	558	311	384
3.0	721	300	636	279	1209	763
	611	601	696	622	123	138
4.0	861	340	884	364	1301	722
	537	495	514	471	97	113
5.0	807	263	838	289	1354	706
	629	534	598	508	82	91

Table S126. Evaluation metrics across n (Cohort 9) (Classification).

Method	n	Accuracy	Error Rate	Precision	Recall	Specificity	F1	Kappa
GBM	0.5	0.586	0.414	0.464	0.695	0.521	0.557	0.586
	1.0	0.571	0.429	0.493	0.813	0.396	0.613	0.571
	1.5	0.603	0.397	0.563	0.634	0.577	0.597	0.603
	2.0	0.609	0.391	0.602	0.698	0.515	0.647	0.609
	3.0	0.592	0.408	0.706	0.541	0.667	0.613	0.592
	4.0	0.607	0.393	0.717	0.616	0.593	0.663	0.607
	5.0	0.601	0.399	0.754	0.562	0.670	0.644	0.601
RF	0.5	0.581	0.419	0.458	0.661	0.534	0.541	0.581
	1.0	0.603	0.397	0.521	0.670	0.555	0.586	0.603
	1.5	0.599	0.401	0.562	0.606	0.593	0.583	0.599
	2.0	0.607	0.393	0.600	0.698	0.512	0.645	0.607
	3.0	0.563	0.437	0.695	0.477	0.690	0.566	0.563
	4.0	0.607	0.393	0.708	0.632	0.564	0.668	0.607
	5.0	0.603	0.397	0.744	0.584	0.637	0.654	0.603
LB	0.5	0.631	0.369	0.536	0.108	0.944	0.179	0.631
	1.0	0.597	0.403	0.570	0.161	0.912	0.251	0.597
	1.5	0.566	0.434	0.545	0.372	0.733	0.442	0.566
	2.0	0.545	0.455	0.542	0.728	0.353	0.621	0.545
	3.0	0.603	0.397	0.613	0.908	0.153	0.732	0.603
	4.0	0.633	0.367	0.643	0.931	0.135	0.761	0.633
	5.0	0.647	0.353	0.657	0.943	0.114	0.775	0.647

Table S127. Confusion matrices across n (Cohort 10) (Classification).

n	GBM		RF		LB	
0.5	135	184	139	231	207	492
	109	438	105	391	37	130
1.0	152	138	205	241	61	43
	161	415	108	312	252	510
1.5	207	150	235	189	49	47
	179	330	151	291	337	433
2.0	216	115	247	143	237	182
	222	313	191	285	201	246
3.0	237	79	353	165	503	298
	298	252	182	166	32	33
4.0	293	80	270	91	74	16
	304	189	327	178	523	253
5.0	255	60	408	138	21	4
	370	181	217	103	604	237

Table S128. Evaluation metrics across n (Cohort 10) (Classification).

Method	n	Accuracy	Error Rate	Precision	Recall	Specificity	F1	Kappa
GBM	0.5	0.662	0.338	0.423	0.553	0.704	0.480	0.662
	1.0	0.655	0.345	0.524	0.486	0.750	0.504	0.655
	1.5	0.620	0.380	0.580	0.536	0.688	0.557	0.620
	2.0	0.611	0.389	0.653	0.493	0.731	0.562	0.611
	3.0	0.565	0.435	0.750	0.443	0.761	0.557	0.565
	4.0	0.557	0.443	0.786	0.491	0.703	0.604	0.557
	5.0	0.503	0.497	0.810	0.408	0.751	0.543	0.503
RF	0.5	0.612	0.388	0.376	0.570	0.629	0.453	0.612
	1.0	0.597	0.403	0.460	0.655	0.564	0.540	0.597
	1.5	0.607	0.393	0.554	0.609	0.606	0.580	0.607
	2.0	0.614	0.386	0.633	0.564	0.666	0.597	0.614
	3.0	0.599	0.401	0.681	0.660	0.502	0.670	0.599
	4.0	0.517	0.483	0.748	0.452	0.662	0.564	0.517
	5.0	0.590	0.410	0.747	0.653	0.427	0.697	0.590
LB	0.5	0.389	0.611	0.296	0.848	0.209	0.439	0.389
	1.0	0.659	0.341	0.587	0.195	0.922	0.293	0.659
	1.5	0.557	0.443	0.510	0.127	0.902	0.203	0.557
	2.0	0.558	0.442	0.566	0.541	0.575	0.553	0.558
	3.0	0.619	0.381	0.628	0.940	0.100	0.753	0.619
	4.0	0.378	0.622	0.822	0.124	0.941	0.215	0.378
	5.0	0.298	0.702	0.840	0.034	0.983	0.065	0.298

Table S129. Confusion matrices across n (Cohort 11) (Classification).

n	GBM		RF		LB	
0.5	73	283	70	278	80	339
	41	432	44	437	34	376
1.0	127	355	95	233	102	317
	25	322	57	444	50	360
1.5	160	313	148	292	144	394
	38	318	50	339	54	237
2.0	197	323	153	216	201	385
	35	274	79	381	31	212
3.0	230	222	231	226	270	350
	76	301	75	297	36	173
4.0	278	154	303	189	245	139
	106	291	81	256	139	306
5.0	321	133	332	143	276	136
	112	263	101	253	157	260

Table S130. Evaluation metrics across n (Cohort 11) (Classification).

Method	n	Accuracy	Error Rate	Precision	Recall	Specificity	F1	Kappa
GBM	0.5	0.609	0.391	0.205	0.640	0.604	0.311	0.609
	1.0	0.542	0.458	0.263	0.836	0.476	0.401	0.542
	1.5	0.577	0.423	0.338	0.808	0.504	0.477	0.577
	2.0	0.568	0.432	0.379	0.849	0.459	0.524	0.568
	3.0	0.641	0.359	0.509	0.752	0.576	0.607	0.641
	4.0	0.686	0.314	0.644	0.724	0.654	0.681	0.686
	5.0	0.704	0.296	0.707	0.741	0.664	0.724	0.704
RF	0.5	0.612	0.388	0.201	0.614	0.611	0.303	0.612
	1.0	0.650	0.350	0.290	0.625	0.656	0.396	0.650
	1.5	0.587	0.413	0.336	0.747	0.537	0.464	0.587
	2.0	0.644	0.356	0.415	0.659	0.638	0.509	0.644
	3.0	0.637	0.363	0.505	0.755	0.568	0.606	0.637
	4.0	0.674	0.326	0.616	0.789	0.575	0.692	0.674
	5.0	0.706	0.294	0.699	0.767	0.639	0.731	0.706
LB	0.5	0.550	0.450	0.191	0.702	0.526	0.300	0.550
	1.0	0.557	0.443	0.243	0.671	0.532	0.357	0.557
	1.5	0.460	0.540	0.268	0.727	0.376	0.391	0.460
	2.0	0.498	0.502	0.343	0.866	0.355	0.491	0.498
	3.0	0.534	0.466	0.435	0.882	0.331	0.583	0.534
	4.0	0.665	0.335	0.638	0.638	0.688	0.638	0.665
	5.0	0.647	0.353	0.670	0.637	0.657	0.653	0.647

L Results for Cohort-Level vs. Individual-Level Time-to-Transplant Curves for Re-Transplants: A Robustness Check for S1 Appendix E

As a robustness check for the analyses in S1 Appendix E, we compare the results obtained from using cohort-level time-to-transplant curves versus those from individual-level curves under the recursive approach. The results for the cohort-level curves are presented on the left, while those for the individual-level curves are displayed on the right.

We use traditional survival analysis to estimate time-to-transplant curves at the individual level. This is because competing risks models provide survival curves only at the cohort level, not at the individual level.

In survival analysis, competing events, such as death before transplant or becoming ineligible for a transplant, are treated as censored data. That is, for these patients, the event of interest (receiving a transplant) is considered as possibly occurring in the future, beyond the study period. This approach ignores the fact that for those patients, the event of interest (transplant) will never occur due to the competing event (e.g., death).

When we use traditional survival analysis (e.g. RSF, DeepSurv, Cox) instead of a competing risks model to estimate the probability of receiving a transplant, we observe higher estimated probabilities of transplant because

- In competing risks setting, each competing event reduces the number of individuals at risk for the event of interest. However, traditional survival analysis methods do not adjust the risk set accordingly. This leads to a bias in estimating the probability of the event of interest, as it assumes a larger pool of individuals who could potentially experience the event than actually exists. Hence, they tend to overestimate the probability of the event of interest.

- Making individual-level estimations allow us to focus on "right tail". In a competing risks model, cohort-level transplant probability estimates average outcomes across all patients, including those with low and high likelihoods of receiving a transplant. In contrast, individual-level estimations highlight patients in the right tail, those with the highest likelihood of transplantation. These individuals are more likely to receive a transplant and have better survival outcomes, leading to higher estimated life expectancies. Consequently, traditional survival models, which focus on individual-level predictions, emphasize this high-likelihood subgroup and identify more potential candidates for trials.

L.1 With Random Survival Forest Model

Table S131. The fraction of the labeled patients - Base case (Cohort-level).

n	C1	C2	C3	C4
0.5	0.7	0.0	1.7	0.2
1.0	1.3	0.2	3.4	1.4
1.5	2.8	1.2	7.0	6.4
2.0	7.0	5.0	14.3	20.4
3.0	24.7	30.5	35.7	60.6

Table S132. The fraction of the labeled patients - Base case (Individual-level).

n	C1	C2	C3	C4
0.5	1.4	0.0	3.4	0.7
1.0	2.5	0.3	5.8	3.4
1.5	4.7	1.9	10.2	9.7
2.0	10.6	7.0	19.4	26.9
3.0	31.5	35.6	42.8	66.8

Table S133. The fraction of the incentivized patients - Keeping inactive (Cohort-level).

n	C1	C2	C3	C4
0.5	0.7	0.0	1.7	0.2
1.0	1.4	0.2	3.8	1.7
1.5	3.3	1.2	7.6	6.9
2.0	7.8	5.4	15.7	22.1
3.0	26.8	31.7	38.1	61.7

Table S134. The fraction of the incentivized patients - Keeping inactive (Individual-level).

n	C1	C2	C3	C4
0.5	1.6	0.0	3.6	1.2
1.0	3.1	0.8	6.9	3.7
1.5	5.8	2.7	12.5	11.7
2.0	12.6	8.7	23.4	31.1
3.0	36.8	39.9	49.1	71.9

Table S135. The fraction of the incentivized patients - Prior living donor (Cohort-level).

n	C1	C2	C3	C4
0.5	3.1	0.5	7.0	3.2
1.0	4.8	1.8	10.6	7.1
1.5	9.0	4.6	17.3	18.0
2.0	16.9	12.5	29.7	38.3
3.0	41.4	43.3	54.1	77.3

Table S136. The fraction of the incentivized patients - Prior living donor (Individual-level).

n	C1	C2	C3	C4
0.5	4.4	1.1	9.1	4.1
1.0	7.2	2.4	13.6	9.6
1.5	11.7	5.8	21.9	22.7
2.0	21.1	15.0	33.6	43.8
3.0	46.1	46.4	57.6	80.5

L.2 With DeepSurv Model

Table S137. The fraction of the labeled patients - Base case (Cohort-level).

n	C1	C2	C3	C4
0.5	1.9	0.1	4.4	0.0
1.0	3.3	0.8	7.6	1.0
1.5	6.3	3.0	12.9	5.9
2.0	13.3	9.2	22.5	18.8
3.0	35.3	37.2	46.2	66.5

Table S138. The fraction of the labeled patients - Base case (Individual-level).

n	C1	C2	C3	C4
0.5	3.5	0.4	7.1	0.5
1.0	5.7	1.1	11.6	2.9
1.5	10.4	3.9	17.7	9.2
2.0	18.0	10.7	28.2	23.3
3.0	41.4	40.1	52.7	68.7

Table S139. The fraction of the incentivized patients - Keeping inactive (Cohort-level).

n	C1	C2	C3	C4
0.5	2.0	0.1	4.7	0.0
1.0	3.5	0.9	8.0	1.1
1.5	7.0	3.2	14.1	6.8
2.0	14.6	9.5	24.0	19.8
3.0	37.3	38.4	49.0	67.2

Table S140. The fraction of the incentivized patients - Keeping inactive (Individual-level).

n	C1	C2	C3	C4
0.5	3.9	0.5	7.7	0.7
1.0	6.5	1.5	12.9	4.0
1.5	12.1	4.9	20.4	11.3
2.0	20.8	12.2	31.6	27.0
3.0	45.9	43.7	57.6	71.8

Table S141. The fraction of the incentivized patients - Prior living donor (Cohort-level).

n	C1	C2	C3	C4
0.5	6.2	1.2	12.0	3.0
1.0	10.0	2.9	18.5	7.8
1.5	16.1	7.6	26.1	16.7
2.0	25.4	16.0	38.5	34.5
3.0	49.3	48.9	64.0	78.6

Table S142. The fraction of the incentivized patients - Prior living donor (Individual-level).

n	C1	C2	C3	C4
0.5	7.6	1.6	14.8	4.9
1.0	11.8	3.9	20.3	10.5
1.5	17.8	8.7	28.1	20.5
2.0	27.9	18.6	40.6	38.4
3.0	51.4	52.0	65.4	81.0

L.3 With Cox Model

Table S143. The fraction of the labeled patients - Base case (Cohort-level).

n	C1	C2	C3	C4
0.5	1.2	0.2	3.3	0.2
1.0	2.4	0.7	6.1	1.7
1.5	5.0	3.0	11.3	7.1
2.0	10.4	9.0	21.7	20.1
3.0	29.1	36.9	45.7	59.2

Table S144. The fraction of the labeled patients - Base case (Individual-level).

n	C1	C2	C3	C4
0.5	2.0	0.2	5.9	0.6
1.0	3.7	1.0	9.1	3.0
1.5	7.4	3.8	15.3	8.6
2.0	13.9	10.2	26.9	23.2
3.0	35.5	39.8	52.0	63.4

Table S145. The fraction of the incentivized patients - Keeping inactive (Cohort-level).

n	C1	C2	C3	C4
0.5	1.3	0.2	3.4	0.2
1.0	2.4	0.8	6.4	1.7
1.5	5.3	3.3	11.9	7.6
2.0	11.1	9.1	22.8	20.9
3.0	30.5	38.0	48.3	60.5

Table S146. The fraction of the incentivized patients - Keeping inactive (Individual-level).

n	C1	C2	C3	C4
0.5	2.3	0.3	6.5	0.6
1.0	4.5	1.3	10.3	3.6
1.5	8.9	4.2	17.7	10.0
2.0	16.4	11.6	29.9	25.3
3.0	40.0	43.1	56.4	67.9

Table S147. The fraction of the incentivized patients - Prior living donor (Cohort-level).

n	C1	C2	C3	C4
0.5	3.6	0.6	9.2	1.5
1.0	6.0	1.8	13.2	4.7
1.5	10.3	5.2	21.6	12.2
2.0	18.3	13.1	34.2	29.4
3.0	42.0	44.5	59.6	71.5

Table S148. The fraction of the incentivized patients - Prior living donor (Individual-level).

n	C1	C2	C3	C4
0.5	5.7	0.9	12.4	2.0
1.0	8.7	2.7	16.8	6.1
1.5	13.9	6.2	25.5	15
2.0	23.3	14.8	38.4	32.8
3.0	46.0	46.3	62.5	73.7
